# Hexanuclear Ln_6_L_6_ Complex Formation by Using an Unsymmetric Ligand

**DOI:** 10.1002/chem.202302497

**Published:** 2023-10-31

**Authors:** Daniel J. Bell, Tongtong Zhang, Niklas Geue, Ciarán J. Rogers, Perdita E. Barran, Alice M. Bowen, Louise S. Natrajan, Imogen A. Riddell

**Affiliations:** ^1^ Department of Chemistry University of Manchester Oxford Road Manchester M13 9PL UK; ^2^ Michael Barber Centre for Collaborative Mass Spectrometry Department of Chemistry The University of Manchester 131 Princess Street Manchester M17DN UK; ^3^ National Research Facility for Electron Paramagnetic Resonance Photon Science Institute The University of Manchester Oxford Road Manchester M13 9PL UK

**Keywords:** lanthanide, luminescence, polynuclear, self-assembly, unsymmetric ligand

## Abstract

Multinuclear, self‐assembled lanthanide complexes present clear opportunities as sensors and imaging agents. Despite the widely acknowledged potential of this class of supramolecule, synthetic and characterization challenges continue to limit systematic studies into their self‐assembly restricting the number and variety of lanthanide architectures reported relative to their transition metal counterparts. Here we present the first study evaluating the effect of ligand backbone symmetry on multinuclear lanthanide complex self‐assembly. Replacement of a symmetric ethylene linker with an unsymmetric amide at the center of a homoditopic ligand governs formation of an unusual Ln_6_L_6_ complex with coordinatively unsaturated metal centers. The choice of triflate as a counterion, and the effect of ionic radii are shown to be critical for formation of the Ln_6_L_6_ complex. The atypical Ln_6_L_6_ architecture is characterized using a combination of mass spectrometry, luminescence, DOSY NMR and EPR spectroscopy measurements. Luminescence experiments support clear differences between comparable Eu_6_L_6_ and Eu_2_L_3_ complexes, with relatively short luminescent lifetimes and low quantum yields observed for the Eu_6_L_6_ structure indicative of non‐radiative decay processes. Synthesis of the Gd_6_L_6_ analogue allows three distinct Gd⋯Gd distance measurements to be extracted using homo‐RIDME EPR experiments.

## Graphical abstract

**Reduced symmetry ligand directs formation of hexanuclear lanthanide complex**: Lanthanide ionic radius and counterions play a role in self‐assembly of a hexagonal Ln_6_L_6_ complex. Structural assignment was achieved by NMR, mass spectrometry and homo‐RIDME EPR experiments that identified three unique Gd⋯Gd distances. The Eu_6_L_6_ complex shows distinct photophysical features from the Eu_2_L_3_ complex generated with a related symmetric ligand.

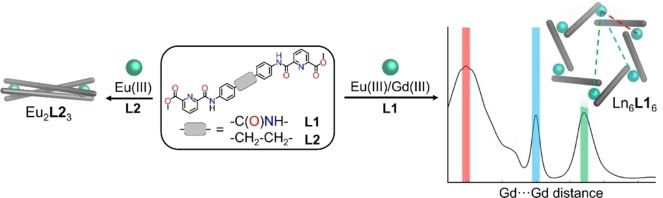

## Introduction

In recent years significant advances in the synthesis and characterization of self‐assembling lanthanide multinuclear architectures^[1]^ have enabled the potential applications of these complexes in imaging,^[2]^ magnetism^[3]^ and sensing^[4]^ to begin to be realized. In particular lanthanide complexes exhibit clear advantages over their transition metal counterparts as they are frequently luminescent,[^4a^, ^5^] are able to incorporate ancillary ligands that do not bridge between multiple ions,^[6]^ and exhibit fewer restrictions on the coordination number and geometry at the metal sites.[^1a^, ^7^]

Challenges of rationally designing self‐assembling lanthanide complexes and characterizing the often paramagnetic complexes do however continue to limit the number and variety of lanthanide architectures published. In particular, the effect of ligand symmetry on lanthanide complex formation has remained underexplored despite a growing body of work demonstrating that incorporation of reduced symmetry components within transition metal–organic assemblies^[8]^ enables the formation of reduced symmetry binding pockets with the capacity to bind complex guests.^[9]^ In addition, reducing ligand symmetry facilitates the incorporation of more functional groups within a ligand of a given size. Unsymmetric ligands^[10]^ are defined in two classes: i) those incorporating a symmetric backbone and differing in their binding sites (also known as heteroditopic), or ii) those which have equivalent binding sites but an unsymmetric backbone. For lanthanide complexes, research into the formation of helicate structures generated with heteroditopic ligands has enabled controlled self‐assembly of bimetallic systems with useful magnetic and imaging properties.^[11]^ The overall architectures formed with the heteroditopic ligands, typically Ln_2_L_3_ helicates, are however not observed to vary from the structures obtained with the parent homoditopic ligands.

**Figure 1:**
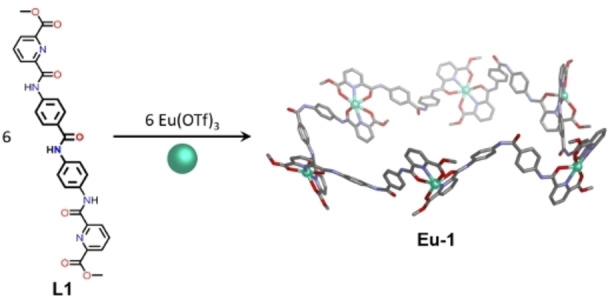
Synthesis of Eu_6_L_6_ circular helicate (**Eu**–**1**) from **L1** and europium(III) triflate. The highest symmetry isomer of **Eu**–**1** is shown for simplicity, however, NMR analysis indicates a mixture of isomers coexist in solution (Supporting Information S4.2). The Eu_6_L_6_ model was generated using *Avogadro* ^[12]^ and does not include counterions or solvent molecules.

To our knowledge no studies with supramolecular lanthanide complexes have been reported where the removal of symmetry within the spacer backbone has been investigated. Herein we report the formation of an unexpected Ln_6_L_6_ complex when an unsymmetric ligand (**L1**; Figure 1) is employed in self‐assembly reactions with lanthanide triflates of appropriate ionic radii. By contrast, utilizing a symmetrical ligand (**L2**) of comparable length with equivalent binding sites generates well‐recognized Ln_2_L_3_ and Ln_4_L_6_ complexes. The hexanuclear architectures are characterized using a combination of NMR, ion mobility mass spectrometry, luminescence and EPR techniques, and their luminescence properties differ from those observed with the Ln_2_L_3_ and Ln_4_L_6_ complexes due to their differing coordination environments.

## Results and Discussion

A new unsymmetric *bis*tridentate ligand (**L1**; Figure 1) was synthesized via the amide condensation of 4,4’‐diaminobenzanilide and the acid chloride of dipicolinate methyl ester. Ligand **L1** incorporates a central amide moiety that reduces the overall symmetry of the molecule compared with classical homoditopic ligands, which commonly display *C*_2_‐symmetry. The central amide moiety thus introduces the possibility for isomer formation in multinuclear species, where the ligands may be arranged in a head‐to‐head or a head‐to‐tail configuration.^[11]^ The amide functionality also provides potential opportunities for hydrogen bonding^[13]^ to guest molecules or adjacent ligands. Based on previously reported work we hypothesize that three equivalents of ligand could be combined with two equivalents of lanthanide metal salt to generate common supramolecular architectures including Ln_2_L_3_ helicates^[14]^ and Ln_4_L_6_ tetrahedra.[^4a^, ^15^] Initial mass spectrometry studies supported formation of a multinuclear species with a 1 : 1 ratio of Eu(III) metal ions to ligand, inconsistent with our initial hypothesis that triple helicate or tetrahedral structures would form. Subsequent characterization supported the formation of a Ln_6_L_6_ circular helicate complex (**1**; Figure 1) in the presence of Eu(III) ions and revealed the effect of lanthanide ionic radius on the outcome of the self‐assembly reactions.

### Characterisation of unexpected Eu_6_L_6_ complex

**Figure 2:**
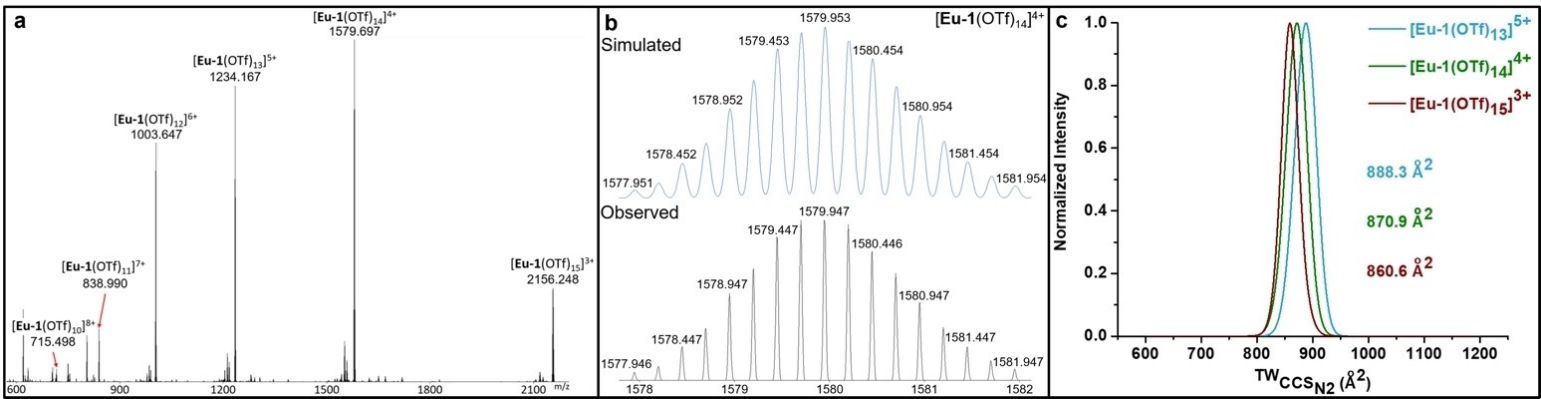
Mass spectra for complex **Eu**–**1**: a) ESI mass spectrum; b) simulated and experimentally observed isotope pattern for [**Eu**–**1**(OTf)_14_]^4+^; and c) IM–MS data for three **Eu**–**1** cations. For each ion, one ^TW^CCS_N2_ distribution from one data set was fitted to a Gaussian distribution. The ionic diameter calculated from the ^TW^CCS_N2_ measurement is consistent with modelling and DOSY experimental data.

Self‐assembly reactions performed in acetonitrile at 333 K with two equivalents of europium(III) triflate and three equivalents of ligand generated a complex, broadened ^1^H NMR spectrum consistent with the coordination of paramagnetic europium ions to **L1**. By contrast, the mass spectrum clearly displayed intense peaks for a single multinuclear complex (**Eu**–**1**) with a Eu_6_L_6_ formula, and ten to fifteen triflate counterions (Figure 2a).

Repeating the self‐assembly reaction with a 1 : 1 Eu : **L1** stoichiometry, reflecting the dominant species observed by mass spectrometry, enabled a better defined ^1^H NMR profile to be obtained (Figure S10). The number of resonances in the ^1^H NMR spectrum is suggestive of isomer formation, with isomers possible due to *cis*/*trans* isomerisation of the amide bond as well as head‐to‐tail coordination isomers^[11]^ arising from the variable orientation the unsymmetric ligand **L1** (Supporting Information S4.2). ^1^H DOSY NMR analysis of this mixture at 6.02 mM Eu(III) concentration supported formation of a single species with a hydrodynamic radius of 1.52 nm, whilst ^1^H DOSY NMR spectra collected at higher Eu(III) concentrations suggested a larger hydrodynamic radii (2.97 nm) indicative of aggregate formation, most likely a dimer. By comparison, DOSY analysis of the ligand in the absence of metal ions gave a hydrodynamic radius of the ligand as 0.62 nm (Figure S3).

We next evaluated the role of triflate counterions in the self‐assembly reaction. Low temperature ^19^F NMR spectra (Figure S12) revealed multiple fluorine environments suggestive of coordination of triflate to the europium metal centers. Relative integration of the ^19^F NMR signals for free and bound triflate supports three triflate ions occupying a unique chemical environment. Self‐assembly reactions performed in CD_3_CN with EuCl_3_ ⋅ 6H_2_O or Eu(NO_3_)_3_ ⋅ 5H_2_O in place of Eu(OTf)_3_ yielded only insoluble products. Complexation reactions of **L1** and Eu(OTf)_3_ in the presence of dodecaisopropylbambus[6]uril which is known to bind triflate counterions,^[16]^ also failed to yield soluble products. Together these results support an active role for triflate in the formation of **Eu**‐**‐1**.^[17]^ We therefore hypothesize that up to three counterions coordinate alongside acetonitrile solvent molecules to fulfill the coordination sphere requirements of the europium(III) ions within the complex. Attempts to observed bound ^13^CD_3_CN, were inconclusive with no signals consistent with bound solvent observable by ^13^C NMR spectroscopy (Figure S18); we attribute this to signal broadening upon coordination to the paramagnetic Eu(III) center.

Further support for the proposed Eu_6_L_6_ circular helicate structure was obtained using ion mobility mass spectrometry (IM–MS), which measures the mass and structure of an analyte in the same experiment.^[18]^ Structural information is provided in form of collisional cross sections (CCS), which correspond to the size and shape of the analyte as well as to the interactions with a neutral buffer gas (here we use nitrogen). Analysis of the different charge states corresponding to complex **Eu**–**1** indicated that the sequential loss of counterions did not significantly alter the ^TW^CCS_N2_ of the cation (Figure 2c), which suggests a minor impact of the triflate counterions on the overall structure.

Mass to charge peaks corresponding to the cation with three or less triflate ions, which we postulate are coordinated directly to the metal center, were not observed under the conditions of the experiment. ^TW^CCS_N2_ values of **Eu**–**1** were converted to an ionic diameter of 3.33 nm, based on the assumption of a hard sphere model,^[19]^ which was in good agreement with the hydrodynamic radius calculated by ^1^H DOSY NMR spectroscopy (Table S12). Closer examination of the mass spectra also revealed evidence for the formation of aggregates consistent with concentration dependent changes in the hydrodynamic radii observed during DOSY NMR analysis.

Structural models of the proposed circular helicate complex with *cis* and *trans* amide configurations, as well as a linear Eu_6_L_6_ structure were generated using *Avogadro* ^[12]^ (Figure S51). The maximum dimension for each model was measured at 3.8, 4.0 and 6.3 nm for the circular helicate with *cis* amide linkages, *trans* amide linkages and the linear structure, respectively. For both helicate models, the maximal diameter of the model was slightly larger than the experimentally determined diameters obtained by DOSY NMR (3.04 nm) and ion mobility mass spectrometry (3.33 nm), indicating that in solution the helicate may exist in a more closely packed configuration (Table S12). Formation of a catenated structure ((Eu_3_L_3_)_2_) could be ruled out on the basis of the collision induced dissociation studies (Figure S48) which indicated fragments of varying sizes were routinely produced and did not show preferential formation of a Eu_3_L_3_ fragment.^[20]^ Despite exhaustive attempts to isolate crystals of a Ln_6_L_6_ complex no suitable conditions were found, this we attribute to the presence of a mixture of isomeric species (Supporting Information S4.2) in solution as well as labile Ln−L bonds which were readily disrupted by many of the solvents introduced during attempts to isolate the complex.

Luminescence studies were also undertaken on the reaction mixture in CD_3_CN, enabling the determination of the lifetime and quantum yield of Eu_6_L_6_. Ligand sensitized europium(III) luminescence (*λ*_exc_=330 nm, *λ*_em_=617 nm) afforded a typical emission spectrum with four discernable bands corresponding to the (^5^D_0_→^7^F_J_ *J*=0−4 transitions). The luminescence lifetime recorded at the emission maximum (617 nm) enabled measurement of the luminescence lifetime (*τ*) as 305 μs and the quantum yield (*Φ*) was determined as 0.9 %. These comparatively low values support our hypothesis that the dipicolinic acid moieties do not fully saturate the lanthanide coordination sphere, and counterions and solvent, which allow for non‐radiative decay pathways, are included within the metal coordination sphere.^[21]^

### Effect of lanthanide salt on supramolecular architecture

Following characterization of the Ln_6_L_6_ structure with europium we sought to explore whether this structure was uniquely formed with europium(III) triflate or if it could be made with alternative lanthanide ions.^[22]^ Mass spectrometric analysis of reaction mixtures generated from 1 : 1 ratios of lanthanide triflates, where Ln=Sm(III), Tb(III) or Gd(III), and **L1** in acetonitrile supported the formation of Ln_6_L_6_ complexes in all cases (Figures S22, S24 and S26). For the Sm(III) reaction mixture DOSY NMR confirmed exclusive formation of a single species with a hydrodynamic radius comparable to that reported for the Eu_6_L_6_ structure under similar conditions (Figure S20). Moreover, pulsed Hahn Echo Detected Field Sweep (EDFS) measurements at 5 K (Figure S53), of the Gd_6_L_6_ complex (**Gd**–**1**) displayed broad signals due to a large distributed zero‐field splitting (ZFS) parameter indicative of Gd(III) coordination to dipicolinic acid moieties.^[23]^

**Figure 3:**
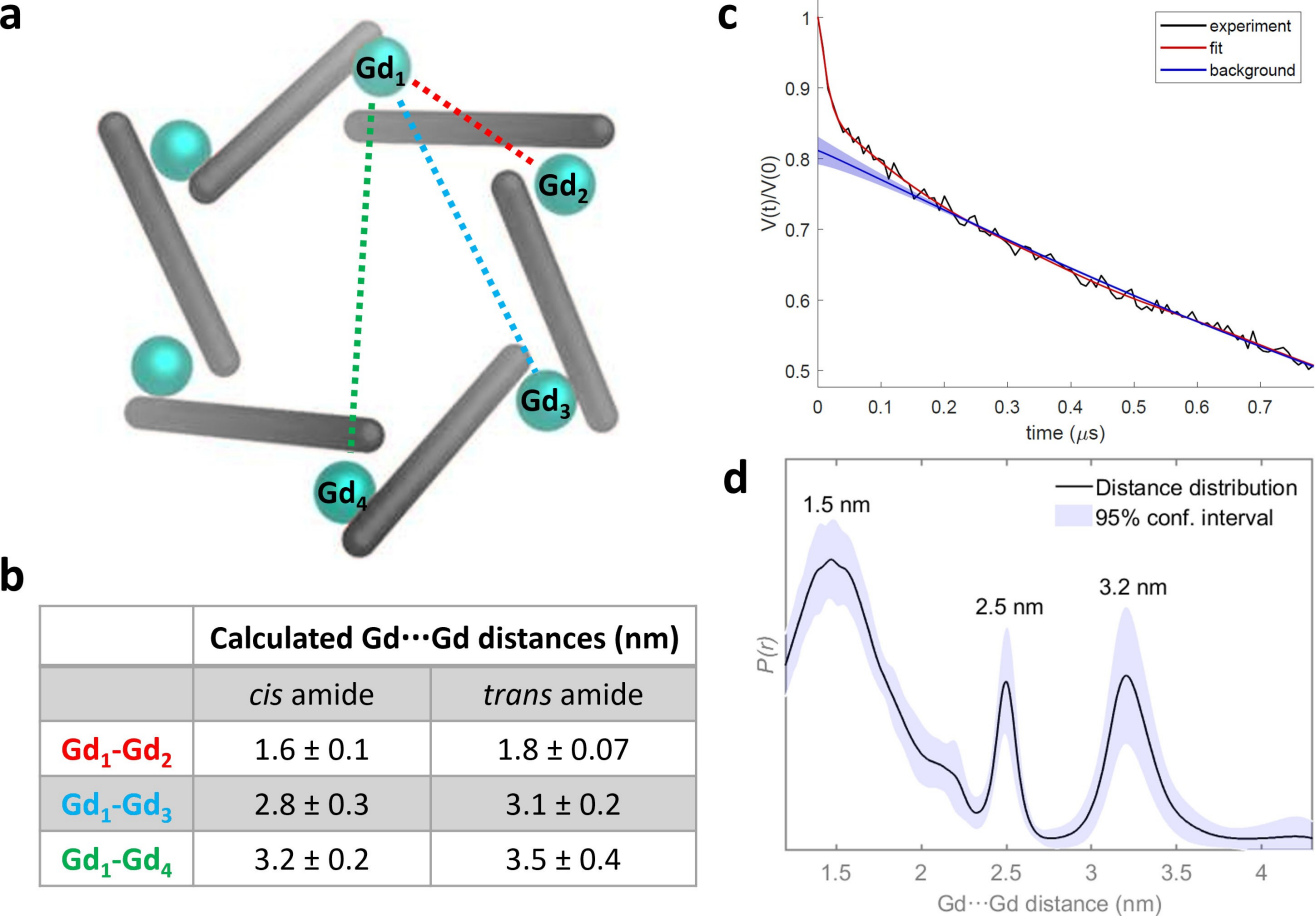
a) Schematic model of M_6_L_6_ circular helicate indicating the three distinct Gd⋯Gd distances (Gd_1_⋯G_d2_, Gd_1_⋯Gd_3_ and Gd_1_⋯Gd_4_); b) tabulated measurements of distances taken from models generated with *Avogadro* ^[12]^ and c) five pulse RIDME trace (black), fit (red) and background (blue) determined by neural network analysis of **Gd**‐**1** after storing at −80 °C, measured at Q‐band (33.62 GHz), at a temperature of 5 K with *T*_mix_=100 μs, d) Corresponding distance distribution with 95 % confidence interval shown as the blue shaded region, with the maxima of each peak of the distribution annotated.

To extract inter‐ spin distance information from the complex, Relaxation Induced Dipolar Modulation Enhancement (RIDME) experiments were performed at Q‐band (33.62 GHz) (Figure S55). This single frequency technique is described in detail elsewhere.^[24]^ Homo‐spin RIDME has been successfully applied for distance determination in Gd(III) containing complexes,^[25]^ but to our knowledge not yet in systems containing more than two Gd(III) centres. The RIDME measurement of the sample in a mixed solution of CD_3_CN : d_8_‐toluene/7 : 3 (200 μM) after being flash frozen in liquid N_2_ and storing at −80 °C gave sharp distances with maxima of 1.5 nm, 2.5 nm and 3.2 nm. These values are consistent with the modelled hexanuclear structure and correlate more closely with the Gd⋯Gd distances in the model where each of the amide bonds is held in a *cis* configuration (Figure 3).

When the lanthanide triflate was changed to lutetium(III) or ytterbium(III), mass spectrometry revealed mixtures of self‐assembled products containing Ln_2_L_3_ and Ln_4_L_6_ supramolecular architectures (Figures S31 and S34). These metal to ligand ratios are commonly observed in metal–organic self‐assembly reactions and correspond to triple helicate and tetrahedral architectures, respectively. Both architectures would be expected to incorporate fully saturated metal ion coordination spheres with three tridentate chelates bound at each metal center, and thus would be differentiated from Ln_6_L_6_ structures by their photophysical properties.

For the mixture generated with lutetium(III) triflate, no evidence for formation of a Lu_6_L_6_ complex was observed under any conditions. The Lu_2_L_3_ and Lu_4_L_6_ complexes were observed to form cleanly by mass spectrometry, whilst ^1^H NMR spectroscopic analysis of the mixture supported multiple ligand environments consistent with formation of constitutional isomers where each ligand resonance was found in several similar chemical shift environments (Figure S30). The DOSY analysis identified two discrete species with hydrodynamic radii of 1.54 and 1.81 nm which are consistent with Lu_2_L_3_ and Lu_4_L_6_ structures, respectively, based on comparison with single‐crystal X‐ray structures^[4a]^ of structurally related complexes found in the literature. By contrast, mass spectrometry analysis of reaction mixtures generated using ytterbium(III) triflate indicated the mixture consisted of three complexes with Yb_2_L_3_, Yb_4_L_6_ and Yb_6_L_6_ metal:ligand ratios. The ^1^H NMR spectrum of a mixture generated from a 1 : 1 combination of Yb(OTf)_3_ and **L** in acetonitrile indicated multiple peaks across the chemical shift range −30 to 25 ppm (Figure S32).

The observation that Yb(III) and Lu(III) are able to generate the predicted self‐assembly products with 2 : 3 M : L stoichiometry in contrast to reactions performed with Sm(III), Tb(III), Gd(III) and Eu(III) can be rationalized when considering the relative nine coordinate ionic radii of the metal ions.^[26]^ Previous reports[^22^, ^27^] have highlighted that the ionic radii of lanthanide ions plays a significant role in determining the outcome of supramolecular self‐assembly reactions. In this study, nona‐coordinated Yb(III) and Lu(III) have the smallest ionic radii (<110 pm) and are able to accommodate three tridentate binding sites for **L1**. The other cations investigated all have notably larger nine coordinate ionic radii (>110 pm)^[26]^ and either support formation of a Ln_6_L_6_ complex, or in the case for La(III) and Nd(III) which have the largest ionic radii, generate featureless spectra and/or precipitate inconsistent with formation of discrete polynuclear species. Reactions with Y(OTf)_3_, which has an intermediate ionic radius larger than Yb(III) but smaller than Tb(III), also failed to generate discrete complexes (Figure S35).

### Role of amide linkage in ligand

**Figure 4:**
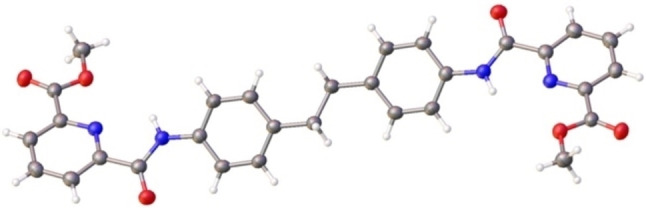
Single crystal X‐ray structure^[28]^ of **L2** incorporating a central −CH_2_CH_2_− linkage in place of the amide (−CONH−) within **L1**.

We next evaluated the outcome of europium self‐assembly reactions with a structurally related ligand (**L2**; Figure 4)) which incorporates a symmetrical ethylene linkage in place of the amide in **L1**.

Ligand **L2** was prepared following a similar synthetic protocol used in the preparation of **L1** and utilized in self‐assembly reactions with europium(III) triflate in a 1 : 1 and 2 : 3 metal: ligand ratio. Following self‐assembly, ^1^H NMR spectroscopic analysis indicated broadened NMR resonances consistent with coordination of the ligand to the paramagnetic metal centre in both reactions. Closer analysis of the ^1^H NMR spectra revealed significant differences between the two samples, with more features being observed in the 2 : 3 metal: ligand ratio reactions. Only one fluorine environment was observed by ^19^F NMR spectroscopy in both reaction mixtures consistent with bulk triflate anions. Mass spectrometry data for both reaction mixtures also supported different compositions. At 1 : 1 metal: ligand ratios, signals corresponding to Eu**L2** and Eu_2_ **L2**_2_ complexes were identified, whilst 2 : 3 metal: ligand ratios resulted in observation of Eu_2_ **L2**_3_ and Eu_2_ **L2**_2_ complexes under comparable measurement conditions. Luminescence measurements of the reaction mixture generated with two equivalents of europium(III) triflate and three equivalents of **L2** supported exclusion of solvent molecules from the inner coordination sphere of the lanthanide ions. Furthermore, significantly longer luminescence lifetimes (1.43 ms) and improved quantum yields of 11 % (versus the 0.9 % recorded for **Eu**–**1**) were recorded for reaction mixtures generated with europium(III) triflate and **L2**.

The structures observed by mass spectrometry for reactions with **L2** all correspond to low nuclearity ions, indicating that the amide linkage within **L1** is required for formation of the hexanuclear Ln_6_L_6_ complex. We thus propose that formation of the Ln_6_L_6_ structure may be governed by the increased rigidity of **L1** versus **L2** which together with the functional groups in the ligand disfavours formation of a close packed Ln_2_L_3_ helicate.

## Conclusions

Exclusive formation of a hexanuclear Ln_6_L_6_ structure is demonstrated with europium(III), samarium(III), terbium(III) and gadolinium(III) triflate salts. The cationic radius is one determinant of the architecture whilst inclusion of the triflate counterion is also shown to be essential for formation of this structure. Replacement of the central amide moiety in ligand **L1** with a symmetric ethylene bridge generates a second ligand (**L2**) of comparable span to **L1**. Despite the similar ligand parameters and shared metal coordination sites, ligand **L2** does not support formation of the Ln_6_L_6_ complex indicating that the central moiety influences the outcome of the self‐assembled structure. An improved understanding the parameters which govern lanthanide based self‐assembly is essential if the full potential of these multinuclear architectures is to be realized. Here, we show how the change in architectural type determined by the cation, anion and ligand can dictate formation of a Eu_6_L_6_ structure with open coordination sites which detrimentally impacts the luminescence properties of the complex but offers the opportunity for appropriately chosen guest molecules to interact with the supramolecular architecture; work towards this is currently ongoing.

## Experimental Section

**General Ln_6_L_6_ self**‐**assembly procedure**: **L1** (1 equiv.) and Ln(OTf)_3_ (1 equiv.) were dissolved in CD_3_CN (0.5 mL), resulting in a pale‐yellow solution. The solution was sealed in a J‐Young NMR tube, and three vacuum/N_2_ fill cycles were applied to degas the solution, before being heated (333 K, 24 hr).

**Eu_6_L1_6_** (**6.02 mM Eu concentration**): ^19^F NMR(470 MHz, 298 K, CD_3_CN): −79.54 (Int=5), −75.76 (Int=1) ppm. DOSY diffusion coefficient (CD_3_CN, 298 K): 4.309×10^−10^ m^2^ s^−1^. Accurate mass *m*/*z*: [Eu_2_ **L1**_2_.(OTf)_2_]^4+^=427.514 (−6.316 ppm), [Eu_2_ **L1**_2_ . (OTf)_3_]^3+^=619.002 (−5.816 ppm), [Eu_6_ **L1**_6_ . (OTf)_10_]^8+^=715.247(−5.173 ppm), [Eu_2_ **L1**_3_ . (OTf)_3_]^3+^=804.056 (−4.726 ppm), [Eu_6_ **L1**_6_.(OTf)_11_]^7+^=838.990 (−5.006 ppm), [Eu_6_ **L1**_6_ . (OTf)_12_]^6+^=1003.980 (−5.478 ppm), [Eu_6_ **L1**_6_ . (OTf)_13_]^5+^=1234.167(−4.537 ppm), [Eu_6_ **L1**_6_ . (OTf)_14_]^4+^=1579.697 (−4.178 ppm), [Eu_6_ **L1**_6_ . (OTf)_15_]^3+^=2156.248 (−3.525 ppm).

**EuL_2_/Eu_2_L2_2_**: **L2**(5.0 mg, 9.28 μmol, 1 equiv.) and Eu(OTf)_3_ (5.56 mg, 9.28 μmol, 1 equiv.) were dissolved in CD_3_CN (0.5 mL), resulting in a pale‐yellow solution. The solution was sealed in a J‐Young NMR tube and subject to three vacuum/N_2_ fill cycles to degas the solution, before being heated (333 K, 24 hr). Accurate mass m/z: [Eu**L2**.(OTf)]^2+^=420.027 (−5.476 ppm), [Eu**L2** . (OTf)_2_]^+^=989.005 (−5.460 ppm), [Eu_2_ **L2**_2_ . (OTf)_4_]^2+^=989.005 (−5.763 ppm)Da.

**Eu_2_L2_3_/Eu_2_L2_2_**: **L2**(5.0 mg, 9.28 μmol, 3 equiv.) and Eu(OTf)_3_ (3.71 mg, 6.19 μmol, 2 equiv.) were dissolved in CD_3_CN (0.5 mL), resulting in a pale‐yellow solution. The solution was sealed in a J‐Young NMR tube and subject to three vacuum/N_2_ fill cycles before being heated (333 K, 24 hr).^19^F (470 MHz, 298 K, CD_3_CN): −79.27 ppm. Accurate mass m/z: [Eu_2_ **L2**_3_ . (OTf)]^5+^=413.468 (−4.595 ppm), [Eu**L2** . (OTf)]^2+^=420.027 (−5.476 ppm), [**L2**+H]^+^=539.190 (−5.749 ppm), [Eu_2_ **L2**_3_ . (OTf)_2_]^4+^=554.073 (−4.332 ppm), [Eu**L2**_2_ . (OTf)]^2+^=689.119 (−4.644 ppm), [Eu_2_ **L2**_3_ . (OTf)_3_]^3+^=789.08 (−5.449 ppm), [Eu**L2** . (OTf)_2_]^+^=989.005 (−5.460 ppm), [Eu_2_ **L2**_2_ . (OTf)_2_]^2+^=989.005 (−5.763 ppm), [Eu_2_ **L2**_3_ . (OTf)_4_]^2+^=1258.098 (−4.690 ppm), [Eu**L2**_2_ . (OTf)_2_]^+^=1527.190 (−4.125 ppm).

Deposition Number(s) 2291650 (for **L2**) contain(s) the supplementary crystallographic data for this paper. These data are provided free of charge by the joint Cambridge Crystallographic Data Centre and Fachinformationszentrum Karlsruhe Access Structures service.

## Conflict of interest

The authors declare no conflict of interest.

1

## Supporting information

As a service to our authors and readers, this journal provides supporting information supplied by the authors. Such materials are peer reviewed and may be re‐organized for online delivery, but are not copy‐edited or typeset. Technical support issues arising from supporting information (other than missing files) should be addressed to the authors.

Supporting Information

## Data Availability

The data that support the findings of this study are available in the supplementary material of this article.

## References

[1a] D. J. Bell , L. S. Natrajan , I. A. Riddell , Coord. Chem. Rev. 2022, 472, 214786;

[1b] X.-Z. Li , C.-B. Tian , Q.-F. Sun , Chem. Rev. 2022, 122, 6374–6458. 35133796 10.1021/acs.chemrev.1c00602

[2a] Z. Wang , L. He , B. Liu , L.-P. Zhou , L.-X. Cai , S.-J. Hu , X.-Z. Li , Z. Li , T. Chen , X. Li , Q.-F. Sun , J. Am. Chem. Soc. 2020, 142, 16409–16419; 32882131 10.1021/jacs.0c07514

[2b] C. D. B. Vandevyver , A.-S. Chauvin , S. Comby , J.-C. G. Bünzli , Chem. Commun. 2007, 1716–1718. 10.1039/b701482a 17457418

[3a] A. Malviya , H. S. Jena , A. K. Mondal , S. Konar , Eur. J. Inorg. Chem. 2015, 2015, 2901–2907;

[3b] J. Lu , V. Montigaud , O. Cador , J. Wu , L. Zhao , X.-L. Li , M. Guo , B. Le Guennic , J. Tang , Inorg. Chem. 2019, 58, 11903–11911; 31192594 10.1021/acs.inorgchem.9b01068

[3c] Y. Zhang , B. Ali , J. Wu , M. Guo , Y. Yu , Z. Liu , J. Tang , Inorg. Chem. 2019, 58, 3167–3174. 30776223 10.1021/acs.inorgchem.8b03249

[4a] X.-Z. Li , L.-P. Zhou , L.-L. Yan , D.-Q. Yuan , C.-S. Lin , Q.-F. Sun , J. Am. Chem. Soc. 2017, 139, 8237–8244; 28558197 10.1021/jacs.7b02764

[4b] X.-Z. Li , L.-P. Zhou , S.-J. Hu , L.-X. Cai , X.-Q. Guo , Z. Wang , Q.-F. Sun , Chem. Commun. 2020, 56, 4416–4419. 10.1039/d0cc00936a 32195500

[5] H.-Y. Wong , W.-S. Lo , K.-H. Yim , G.-L. Law , Chem 2019, 5, 3058–3095.

[6a] D. Guo , C.-Y. Duan , F. Lu , Y. Hasegawa , Q.-J. Meng , S. Yanagida , Chem. Commun. 2004, 1486–1487; 10.1039/b403519d 15216343

[6b] Y. Zhou , H. Li , T. Zhu , T. Gao , P. Yan , J. Am. Chem. Soc. 2019, 141, 19634–19643. 31747264 10.1021/jacs.9b07178

[7] J.-C. G. Bünzli , J. Coord. Chem. 2014, 67, 3706–3733.

[8] J. E. M. Lewis , A. Tarzia , A. J. P. White , K. E. Jelfs , Chem. Sci. 2020, 11, 677–683. 10.1039/c9sc05534g PMC8146399 34123040

[9] J. E. M. Lewis , J. D. Crowley , ChemPlusChem 2020, 85, 815–827. 32364332 10.1002/cplu.202000153

[10] D. Tripathy , N. B. Debata , K. C. Naik , H. S. Sahoo , Coord. Chem. Rev. 2022, 456, 214396.

[11] T. B. Jensen , R. Scopelliti , J.-C. G. Bünzli , Inorg. Chem. 2006, 45, 7806–7814. 16961372 10.1021/ic0608501

[12a] Avogadro: an open-source molecular builder and visualization tool. Version 1.2.0. http://avogadro.cc/;

[12b] M. D. Hanwell , D. E. Curtis , D. C. Lonie , T. Vandermeersch , E. Zurek , G. R. Hutchison , J. Cheminformatics 2012, 4, 17. 10.1186/1758-2946-4-17 PMC3542060 22889332

[13a] Y. Jiao , H.-y. He , J.-Q. Yin , L. Zhou , C. He , C.-Y. Duan , Inorg. Chem. Commun. 2016, 73, 129–133;

[13b] J. Zhang , C. He , C. Duan , Inorg. Chem. Commun. 2015, 54, 41–44;

[13c] S. Yi , V. Brega , B. Captain , A. E. Kaifer , Chem. Commun. 2012, 48, 10295–10297; 10.1039/c2cc35095e 22983087

[13d] D. Yang , L. K. S. von Krbek , L. Yu , T. K. Ronson , J. D. Thoburn , J. P. Carpenter , J. L. Greenfield , D. J. Howe , B. Wu , J. R. Nitschke , Angew. Chem. Int. Ed. 2021, 60, 4485–4490. 10.1002/anie.202014568 33217126

[14] M. Elhabiri , J. Hamacek , J. -Claude , G. Bünzli , A.-M. Albrecht-Gary , Eur. J. Inorg. Chem. 2004, 2004, 51–62.

[15a] K.-H. Yim , C.-T. Yeung , M. R. Probert , W. T. K. Chan , L. E. Mackenzie , R. Pal , W.-T. Wong , G.-L. Law , Commun. Chem. 2021, 4, 116; 36697590 10.1038/s42004-021-00553-8 PMC9814731

[15b] C.-T. Yeung , K.-H. Yim , H.-Y. Wong , R. Pal , W.-S. Lo , S.-C. Yan , M. Yee-Man Wong , D. Yufit , D. E. Smiles , L. J. McCormick , S. J. Teat , D. K. Shuh , W.-T. Wong , G.-L. Law , Nat. Commun. 2017, 8, 1128. 29066720 10.1038/s41467-017-01025-1 PMC5783948

[16] L. Jašíková , M. Rodrigues , J. Lapešová , T. Lízal , V. Šindelář , J. Roithová , Faraday Discuss. 2019, 220, 58–70. 31503271 10.1039/c9fd00038k PMC8609304

[17] J.-F. Lemonnier , L. Guénée , G. Bernardinelli , J.-F. Vigier , B. Bocquet , C. Piguet , Inorg. Chem. 2010, 49, 1252–1265. 20050599 10.1021/ic902314f

[18a] V. Gabelica , A. A. Shvartsburg , C. Afonso , P. Barran , J. L. P. Benesch , C. Bleiholder , M. T. Bowers , A. Bilbao , M. F. Bush , J. L. Campbell , I. D. G. Campuzano , T. Causon , B. H. Clowers , C. S. Creaser , E. De Pauw , J. Far , F. Fernandez-Lima , J. C. Fjeldsted , K. Giles , M. Groessl , C. J. Hogan Jr , S. Hann , H. I. Kim , R. T. Kurulugama , J. C. May , J. A. McLean , K. Pagel , K. Richardson , M. E. Ridgeway , F. Rosu , F. Sobott , K. Thalassinos , S. J. Valentine , T. Wyttenbach , Mass Spectrom. Rev. 2019, 38, 291–320; 30707468 10.1002/mas.21585 PMC6618043

[18b] E. Christofi , P. Barran , Chem. Rev. 2023, 123, 2902–2949; 36827511 10.1021/acs.chemrev.2c00600 PMC10037255

[18c] N. Geue , R. E. P. Winpenny , P. E. Barran , Chem. Soc. Rev. 2022, 51, 8–27. 34817479 10.1039/d0cs01550d

[19a] P. Bonakdarzadeh , F. Topić , E. Kalenius , S. Bhowmik , S. Sato , M. Groessl , R. Knochenmuss , K. Rissanen , Inorg. Chem. 2015, 54, 6055–6061; 26039343 10.1021/acs.inorgchem.5b01082

[19b] A. Kiesilä , L. Kivijärvi , N. K. Beyeh , J. O. Moilanen , M. Groessl , T. Rothe , S. Götz , F. Topić , K. Rissanen , A. Lützen , E. Kalenius , Angew. Chem. Int. Ed. 2017, 56, 10942–10946. 10.1002/anie.201704054 28665506

[20] A. Kruve , K. Caprice , R. Lavendomme , J. M. Wollschläger , S. Schoder , H. V. Schröder , J. R. Nitschke , F. B. L. Cougnon , C. A. Schalley , Angew. Chem. Int. Ed. 2019, 58, 11324–11328. 10.1002/anie.201904541 31173448

[21] S. W. Magennis , S. Parsons , Z. Pikramenou , Chem. Eur. J. 2002, 8, 5761–5771. 12693058 10.1002/1521-3765(20021216)8:24<5761::AID-CHEM5761>3.0.CO;2-H

[22] K.-H. Yim , C.-T. Yeung , H.-Y. Wong , G.-L. Law , Inorg. Chem. Front. 2021, 8, 2952–2964.

[23] M. Gordon-Grossman , I. Kaminker , Y. Gofman , Y. Shai , D. Goldfarb , Phys. Chem. Chem. Phys. 2011, 13, 10771–10780. 21552622 10.1039/c1cp00011j

[24] S. Milikisyants , F. Scarpelli , M. G. Finiguerra , M. Ubbink , M. Huber , J. Magn. Reson. 2009, 201, 48–56. 19758831 10.1016/j.jmr.2009.08.008

[25a] S. Razzaghi , M. Qi , A. I. Nalepa , A. Godt , G. Jeschke , A. Savitsky , M. Yulikov , J. Phys. Chem. Lett. 2014, 5, 3970–3975; 26276479 10.1021/jz502129t

[25b] A. Collauto , V. Frydman , M. D. Lee , E. H. Abdelkader , A. Feintuch , J. D. Swarbrick , B. Graham , G. Otting , D. Goldfarb , Phys. Chem. Chem. Phys. 2016, 18, 19037–19049; 27355583 10.1039/c6cp03299k

[25c] M. Azarkh , A. Bieber , M. Qi , J. W. A. Fischer , M. Yulikov , A. Godt , M. Drescher , J. Phys. Chem. Lett. 2019, 10, 1477–1481. 30864799 10.1021/acs.jpclett.9b00340 PMC6625747

[26] R. D. Shannon , Acta Cryst. A32 1976, 751–767.

[27a] G. Li , X. Zhao , Q. Han , L. Wang , W. Liu , Dalton Trans. 2020, 49, 10120–10126; 32662479 10.1039/d0dt01711f

[27b] A. McRobbie , A. R. Sarwar , S. Yeninas , H. Nowell , M. L. Baker , D. Allan , M. Luban , C. A. Muryn , R. G. Pritchard , R. Prozorov , G. A. Timco , F. Tuna , G. F. S. Whitehead , R. E. P. Winpenny , Chem. Commun. 2011, 47, 6251–6253. 10.1039/c1cc11516b 21552574

[28] Deposition Number 2291650 (for **L2**), contains the supplementary crystallographic data for this paper. These data are provided free of charge by the joint Cambridge Crystallographic Data Centre and Fachinformationszentrum Karlsruhe Structures service.

