# Supplementary material for: Hexanuclear Ln6L6 Complex Formation by Using an Unsymmetric Ligand
Source: Chemistry. 2023 Oct 31;29(71):e202302497. doi: 10.1002/chem.202302497 (PMC10946940; doi:10.1002/chem.202302497)
Supplement: Supplementary file 1 — Supporting Information [file CHEM-29-0-s001.pdf]

Chemistry–A European Journal

Supporting Information

Hexanuclear Ln_6L_6 Complex Formation by Using an Unsymmetric Ligand

Daniel J. Bell, Tongtong Zhang, Niklas Geue, Ciarán J. Rogers, Perdita E. Barran, Alice M. Bowen, Louise S. Natrajan,* and Imogen A. Riddell*

Table of Contents

S1 Synthesis and Characterisation *2*

S1.1. Materials and Methods 2

S1.2. Ligand Synthesis 3

S1.3. Self-Assembly Reactions with Unsymmetrical Ligand L1 9

S1.4. Self-Assembly Reactions with Symmetrical Ligand L2 28

S2 X-Ray Crystallography *32*

S3 Ion Mobility Mass Spectrometry *34*

S3.1. Ion mobility mass spectrometry for CCS determination 34

S3.2. Collision induced dissociation (CID) study 36

S4 Proposed structure of Eu-1 .... *41*

S4.1. Modelling of Eu-1 41

S4.2. Potential head to tail isomer formation with unsymmetric ligand L2 42

S5 EPR Measurements *42*

S6 References *46*

S1 Synthesis and Characterisation

S1.1. Materials and Methods

Unless otherwise specified, all starting materials were purchased from commercial sources and used as supplied. 6-(Methoxycarbonyl)picolinic acid was synthesised via a modified literature procedure.[1] NMR spectra were recorded on a Bruker DPX500 MHz, or a Bruker Avance III 400MHz. Chemical shifts (δ) for ^{1}H NMR spectra are reported in parts per million (ppm) and are reported relative to the solvent residual peak. DOSY NMR spectra were recorded on a Bruker Avance II+ 500 with a Bruker PA BBI 500S2 H-BB-D-05 Z 5mm probe. High-resolution mass spectra were acquired using a Thermo Orbitrap Exactive Plus Extended Mass Range. IM-MS measurements were carried out using a Waters SYNAPT XS High Resolution Mass Spectrometer. All steady state emission and excitation spectra were recorded on an Edinburgh Instrument FP920 Phosphorescence Lifetime Spectrometer (Edinburgh Instruments, Livingston, Scotland) equipped with a 450 watt steady state xenon lamp, a 5 watt microsecond pulsed xenon flashlamp, (with single 300 mm focal length excitation and emission monochromators in Czerny Turner configuration[2]), and a red sensitive photomultiplier in peltier (air cooled) housing (Hamamatsu R928P). All spectra are reported corrected for the detector response and excitation source using the correction files supplied in the software. Lifetime data were recorded following excitation with the microsecond flashlamp using multichannel scaling. Lifetimes were obtained by tail fit on the data obtained and quality of fit judged by minimisation of reduced chi-squared and residuals squared. Absolute quantum yields were measured using an integration sphere (Edinburgh Instruments) and determined according to the manufacturer's instructions. ATR-FTIR spectroscopy was carried out in the solid phase using a Bruker Alpha FT-IR spectrometer using 4 cm^{-1} resolution and 24 scans, with data was collected with OPUS 7.2 software. DOSY experiments were performed with a maximum gradient strength was 56 G/cm. The standard Bruker pulse program, dstebpg3s pulse program[3], gradient strength of 56 G/cm, gradient recovery delay of 200 μs and a 12-point gradient between 5-90% gradient strength (5, 27.5, 38.6, 47.2, 54.4, 60.8, 66.6, 71.9, 76.8, 81.4, 85.8 and 90) and all data was collected at 298 K. The dataset was processed using GNAT[4] and the hydrodynamic radii were calculated using the Stokes-Einstein equation:

$$r = \frac{k_B T}{6\pi\eta D}$$

k_B = Boltzmann constant, T = temperature, η = viscosity of solvent, D = diffusion coefficient

S1.2. Ligand Synthesis

Ligand 1, L1: 6-(Methoxycarbonyl)picolinic acid (0.500 g, 2.50 mmol) was heated to reflux temperature at 353 K in $SOCl_2$ (4 mL) and DMF (3 drops) for 1 hour. The solvent was removed under reduced pressure, yielding a yellowy white powder. 4,4'-diaminobenzanilide (0.227 g, 1.00 mmol) was added and suspended in anhydrous DCM (15 mL). The suspension was cooled to 273 K, and triethylamine (NEt_3) (1.0 mL) was added dropwise. The reaction was stirred for 1 hour at 273 K and subsequently for 16 hours at room temperature during which time precipitation had occurred. The reaction was filtered, and the precipitate was washed with DCM (5 mL) and hexane (5 mL) and dried under vacuum suction, yielding a pale yellow powder (0.424 g, 0.767 mmol, 76.7%). ^{1}H (500 MHz, 298 K, DMSO-d_6): δ = 10.73 (s, 2H, H_h), 10.44 (s, 2H, H_k), 10.25 (s, 2H, H_e), 8.35 (ddd, $^4J_{HH}$ = 2.15, $^3J_{HH}$ = 8.35, $^2J_{HH}$ = 18.95 Hz, 2H, H_d, H_l), 8.28 (m, J = 1.5, 2.95, 9.05 Hz, 4H, H_b, H_c, H_m, H_n), 8.03 (ap s, 4H, H_f, H_g), 7.84 (dd, $^4J_{HH}$ = 2.75, $^3J_{HH}$ 8.75 Hz, 2H, H_i), 7.80 (dd, $^4J_{HH}$ = 2.75, $^3J_{HH}$ = 8.75 Hz, 2H, H_j), 3.99 (s, 3H, Ha), 3.98 (s, 3H, H_o) ppm. ^{13}C (125 MHz, 298 K, DMSO-d_6): δ = 165.2 (C_b, C_x), 163.0 (C_m), 162.3 (C_h, C_r), 151.1 (C_g), 150.9 (C_s), 147.1 (C_c), 147.0 (C_w), 141.4 (C_i), 140.3 (C_e), 140.2 (C_u), 136.0 (C_n), 134.1 (C_q), 130.7 (C_l), 129.0 (C_k), 128.1 (C_d), 127.9 (C_v), 126.4 (C_f), 126.1 (C_t), 121.2 (C_p), 121.1 (C_o), 120.1 (C_j), 53.3 (C_a), 53.3 (C_y) ppm. DOSY diffusion coefficient (DMSO-d_6, 298 K): 1.663 x 10^{-10} m^2s^{-1}. [ESI-MS$^+$]: $[M + Na]^+$, calculated = 576.15, observed = 576.2. ATR-FTIR (cm^{-1}): 3443 (N-H, asymmetric stretch), 3295 (N-H, symmetric stretch), 1725 (C=O), 1669 (C=O), 1583 (C=C, aromatic), 1512 (C=C, ring vibration), 1437 (C-O, stretch), 1406 (C-O, stretch), 1296 (C-N, stretch), 1078 (C-N, stretch), 838 (N-H, bend), 745 (N-H, rocking), 685 (C-H, aromatic), 650 (C-H, aromatic).

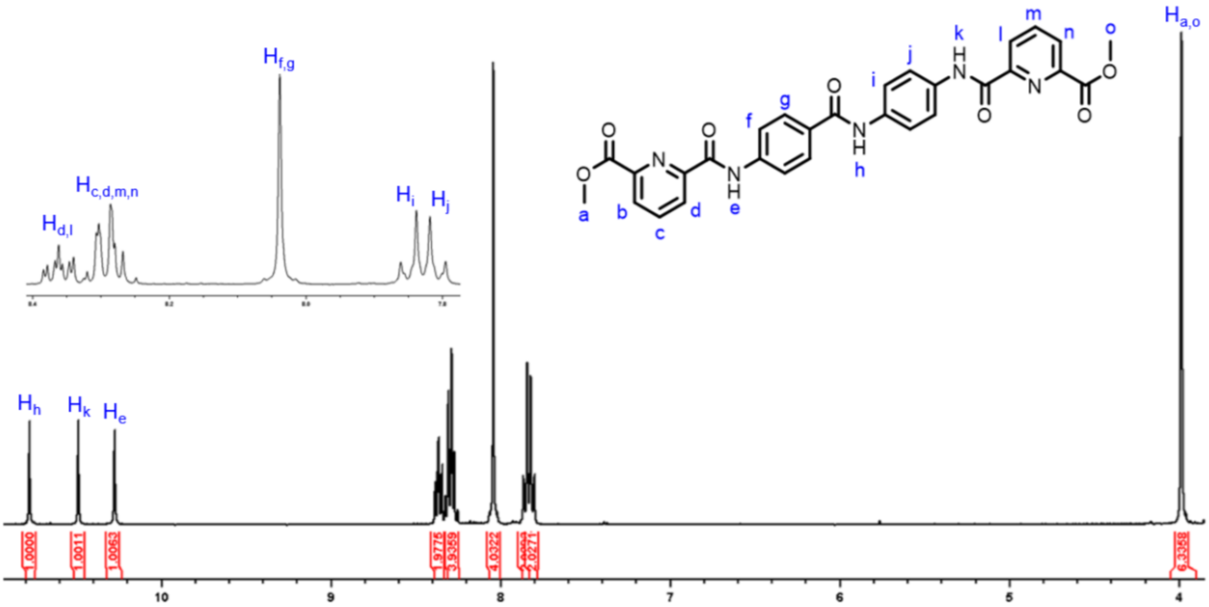

Figure S1. ^{1}H NMR spectrum (500 MHz, 293 K, DMSO-d_6) of **L1** between 4 – 11 ppm, with an insert of the aromatic region highlighting peak multiplicities.

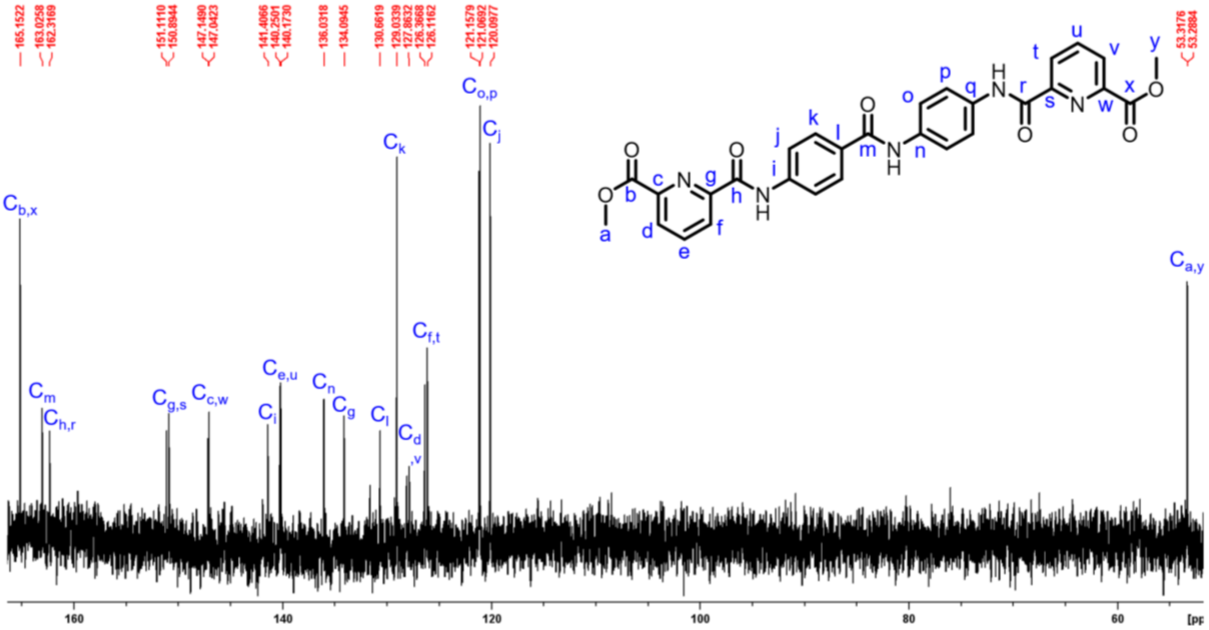

Figure S2. Assigned ^{13}C NMR spectrum (125 MHz, 293 K, DMSO-d_6) of **L1**.

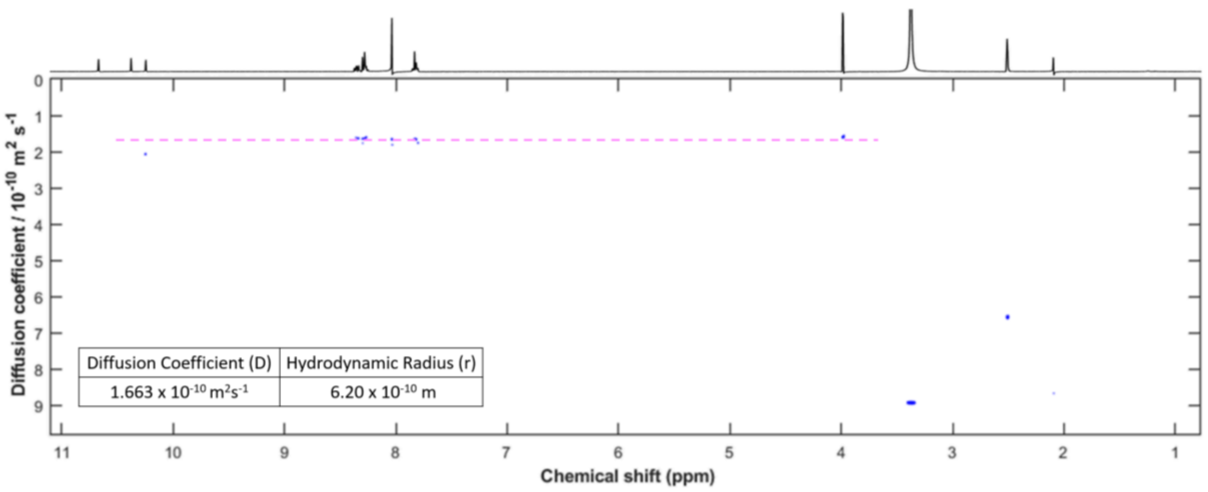

Figure S3. ^{1}H DOSY NMR spectrum (298 K, DMSO-d_6) of **L1**.

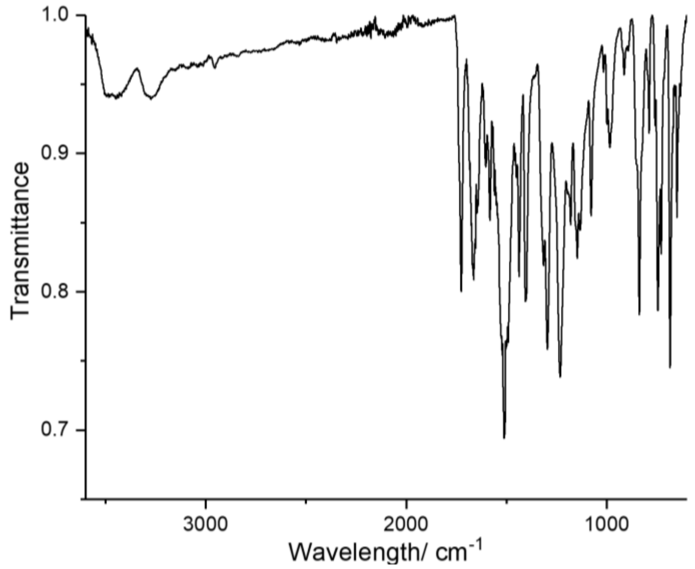

Figure S4. ATR-FTIR spectra of **L1**. Characteristic peaks observed at 3443 – 3295 cm^{-1} (N-H stretches), 1725 and 1669 cm^{-1} (C=O), 1437 cm^{-1} (C-O, stretch), 1296 and 1078 cm^{-1} (C-N, stretch).

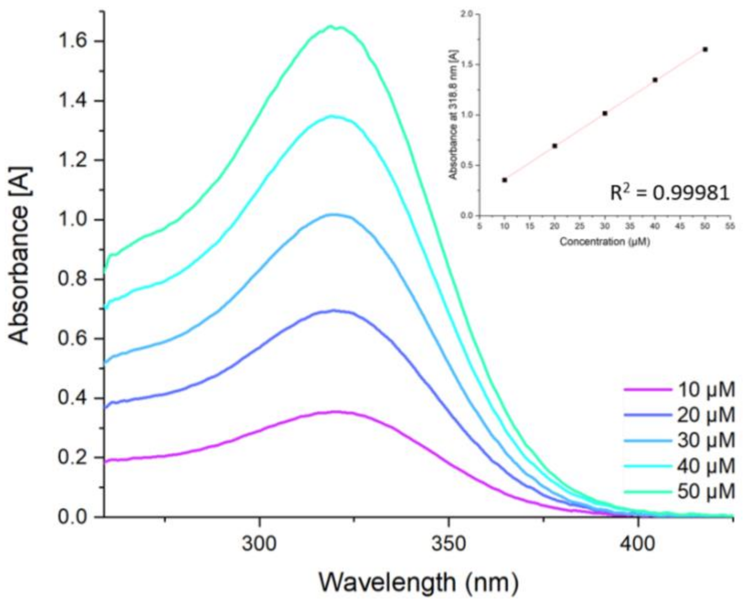

Figure S5. Absorption spectra of **L1** at varying concentrations (10 – 50 µM) in DMSO, with insert showing the R^2 value at the absorbance maxima (318.8 nm).

Ligand 2, L2: 6-(Methoxycarbonyl)picolinic acid (0.300 g, 1.50 mmol) was heated to reflux temperature at 353 K in $SOCl_2$ (3 mL) and DMF (3 drops) for 1 hour. The solvent was then removed under reduced pressure to yield a yellowy white powder. 4,4′-Ethylenedianiline (0.096 g, 0.45 mmol) was added and suspended in anhydrous DCM (20 mL). The solution was cooled to 273 K, and NEt_3 (0.7 mL) was added dropwise, changing the colour of the solution from brown to orange. Stirring at 273 K was maintained for 1 hour. The solution was then allowed to warm to room temperature and was stirred for 96 hours, resulting in a dulling of the orange solution towards brown. The solution was then washed with saturated $NaHCO_3$ solution (20 mL) and brine (20 mL), dried over $MgSO_4$ and filtered. The solvent was removed under reduced pressure yielding a light brown powder (0.124 g, 0.231 mmol, 38.6 %). ^{1}H (400 MHz, 298 K, $CDCl_3$): δ = 9.94 (s, 2H, H_e), 8.41 (d, 2H, $^3J_{HH}$ = 7.70 Hz, H_d), 8.19 (d, 2H, $^3J_{HH}$ = 7.70 Hz, H_b), 7.99 (t, 2H, $^3J_{HH}$ = 7.75 Hz, H_c), 7.64 (d, 4H, $^3J_{HH}$ = 8.35 Hz, H_f), 7.12 (d, 4H, $^3J_{HH}$ = 8.30 Hz, H_g), 3.98 (s, 6H, H_a), 2.86 (s, 4H, H_h) ppm. ^{13}C (126 MHz, 298 K, $CDCl_3$): δ = 164.9 (C_b), 161.1 (C_h), 150.3 (C_g), 146.4 (C_c), 138.8 (C_e), 138.0 (C_i), 135.4 (C_l), 129.1 (C_k), 127.4 (C_d), 125.6 (C_f), 120.1 (C_j), 53.0 (C_a), 37.4 (C_m) ppm. [ESI-MS$^+$]: Observed mass: $[M + Na]^+$ = 561.2 Da, calculated mass: $[M + Na]^+$ = 561.55 Da. ATR-FTIR (cm^{-1}): 3423 (N-H, asymmetric stretch), 3295 (N-H, symmetric stretch), 1720 (C=O), 1675 (C=O), 1585 (C=C, aromatic), 1523 (C=C, ring vibration), 1437 (C-O, stretch), 1409 (C-O, stretch), 1290 (C-N, stretch), 1072 (C-N, stretch), 835 (N-H, bend), 745 (N-H, rocking), 646 (C-H, aromatic).

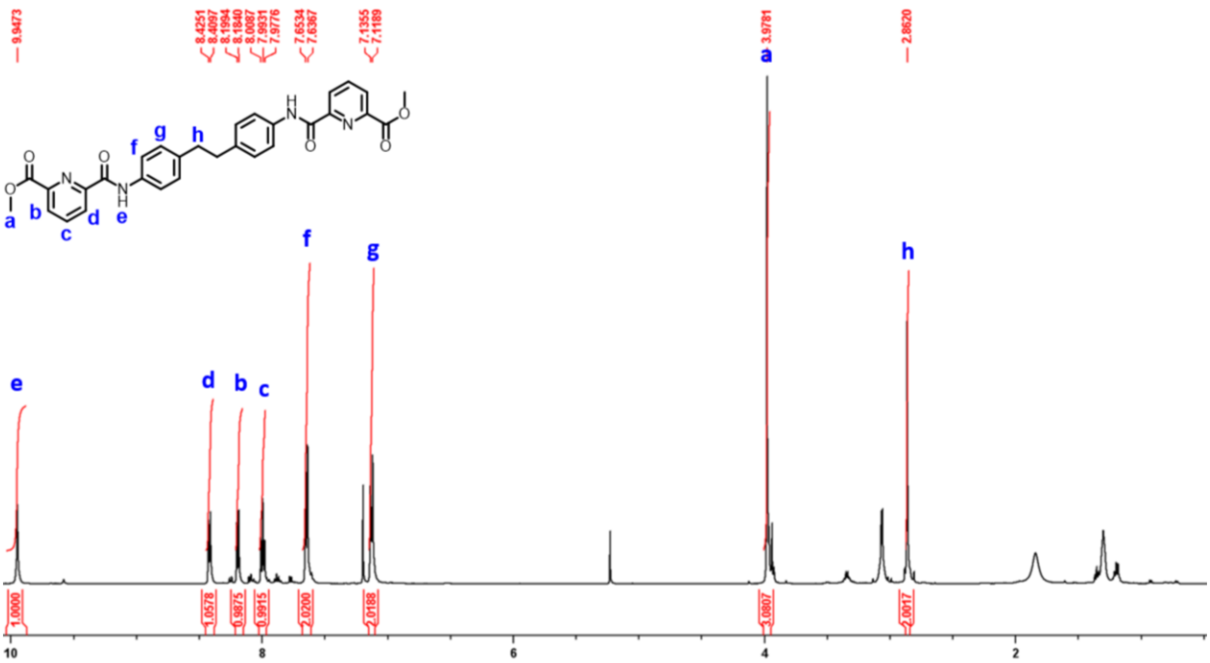

Figure S6. ^{1}H NMR spectrum (400 MHz, 293 K, $CDCl_3$) of **L2**.

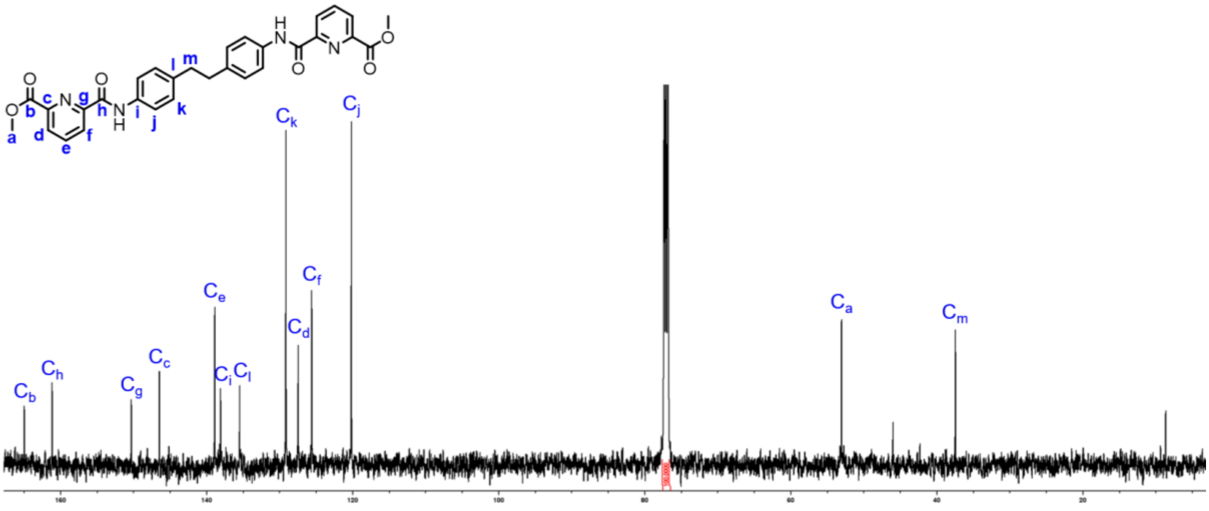

Figure S7. ^{13}C NMR spectrum (125 MHz, 293 K, $CDCl_3$) of **L2**.

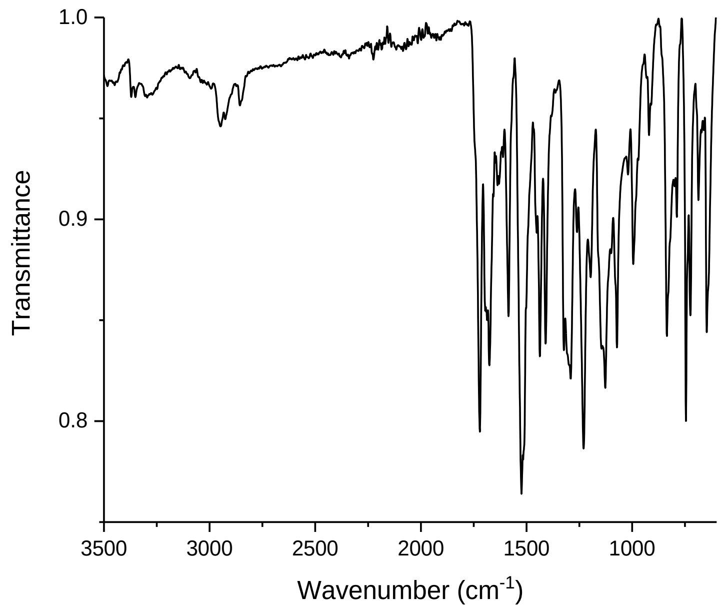

Figure S8. ATR-FTIR spectrum of **L2** between 500 – 4000 cm^{-1}. Characteristic peaks observed at 3423 – 3295 cm^{-1} (N-H stretches), 1720 cm^{-1} and 1675 cm^{-1} (C=O), 1437 cm^{-1} (C-O, stretch), 1290 cm^{-1} and 1072 cm^{-1} (C-N, stretch).

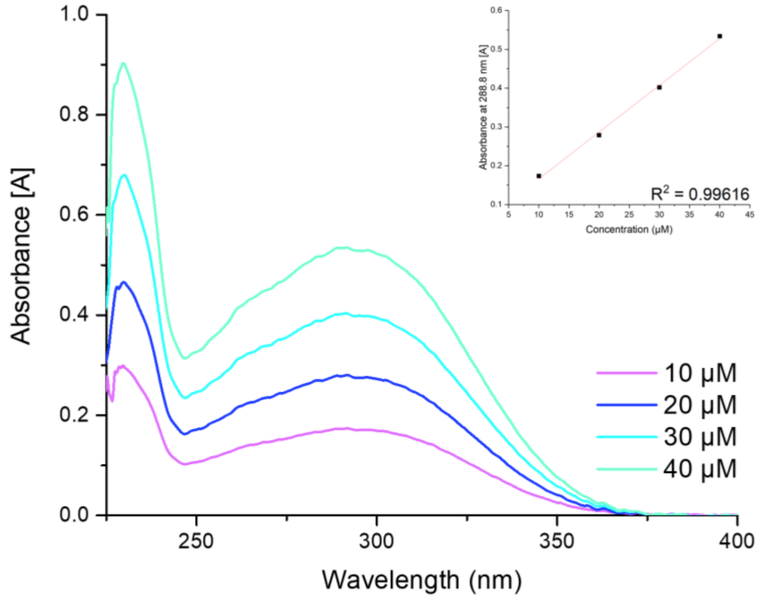

Figure S9. UV-vis absorption spectra of **L2** at varying concentrations (10 – 40 μM) in $CHCl_3$, with insert showing the R^2 value at the absorbance maxima (288.8 nm).

S1.3. Self-Assembly Reactions with Unsymmetrical Ligand L1

18.06 mM Eu. **L1** (5.0 mg, 9.03 μmol, 1 eq.) and $Eu(OTf)_3$ (5.41 mg, 9.03 μmol, 1 eq.) were dissolved in CD_3CN (0.5 mL), resulting in a pale-yellow solution, which was added to a J-Young NMR tube. The tube was sealed, and three vacuum/N_2 fill cycles were applied to degas the solution, before being heated (333 K, 24 hr). DOSY diffusion coefficient (CD_3CN, 298 K): 2.10×10^{-10} m^2s^{-1}.

6.02 mM Eu. **L1** (1.67 mg, 3.01 μmol, 1 eq.) and $Eu(OTf)_3$ (1.80 mg, 3.01 μmol, 1 eq.) were dissolved in CD_3CN (0.5 mL), resulting in a pale-yellow solution, which was added to a J-Young NMR tube. The tube was sealed, and three vacuum/N_2 fill cycles were applied to degas the solution, before being heated (333 K, 24 hr). DOSY diffusion coefficient (CD_3CN, 298 K): 4.309×10^{-10} m^2s^{-1}. ^{19}F (470 MHz, 298 K, CD_3CN): -79.54 (Int = 5), -75.76 (Int = 1) ppm. Accurate mass m/z: [**Eu-1**$(OTf)_{10}$]$^{8+}$ = 715.247, [Eu_2**L1**$_3(OTf)_3$]$^{3+}$ = 804.056, [**Eu-1**$(OTf)_{11}$]$^{7+}$ = 838.990, [**Eu-1**$(OTf)_{12}$]$^{6+}$ = 1003.980, [**Eu-1**$(OTf)_{13}$]$^{5+}$ = 1234.167, [**Eu-1**$(OTf)_{14}$]$^{4+}$ = 1579.697, [**Eu-1**$(OTf)_{15}$]$^{3+}$ = 2156.248.

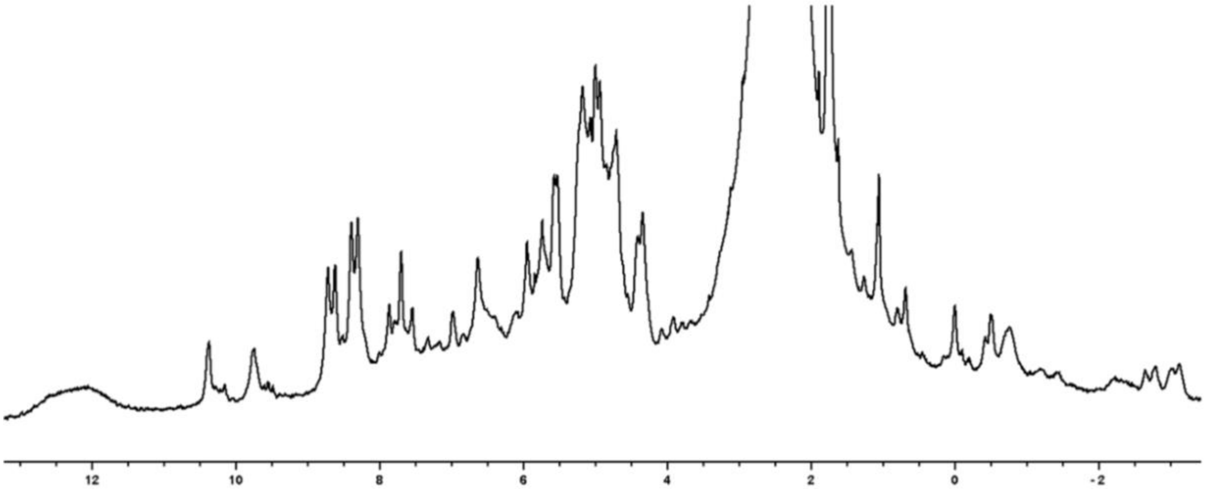

Figure S10. ^1H NMR spectrum (400 MHz, 293 K, CD_3CN) of the complexation products of [**Eu-1**$(OTf)_{18}$].

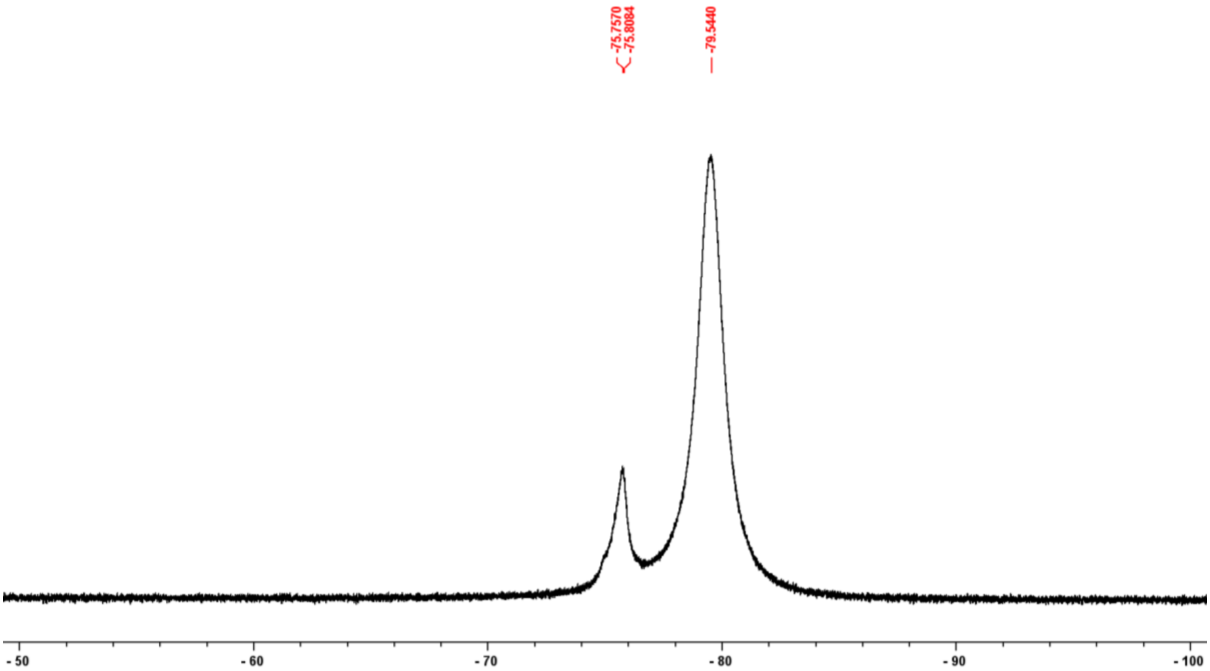

Figure S11. ^{19}F NMR spectrum (470 MHz, 298 K, CD_3CN) of [**Eu-1**$(OTf)_{18}$].

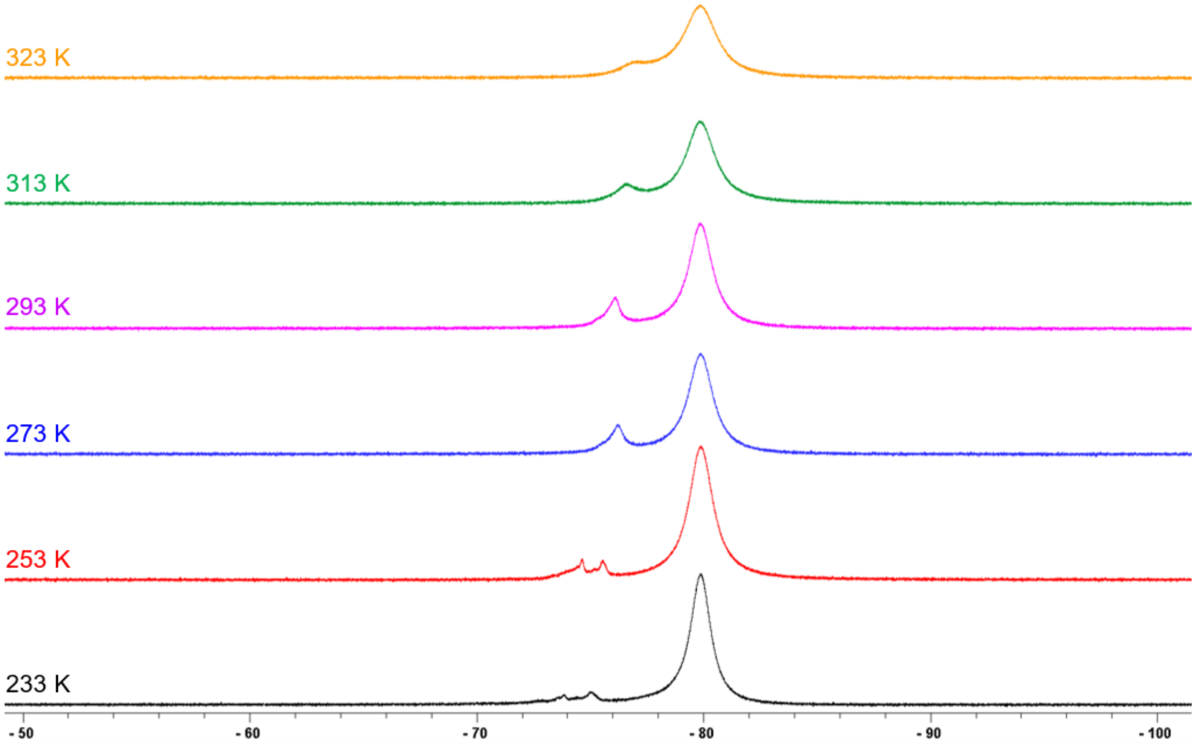

Figure S12. Variable temperature ^{19}F NMR spectra (470 MHz, CD_3CN) of [**Eu-1**$(OTf)_{18}$] between 233 K and 323 K.

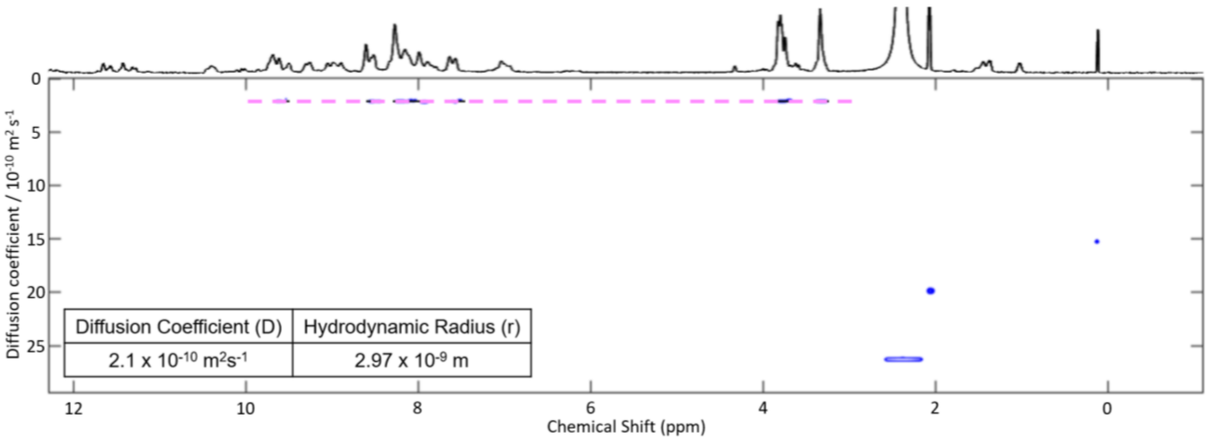

Figure S13. ^{1}H DOSY NMR spectrum (298 K, CD_3CN) of [**Eu-1**$(OTf)_{18}$] ([Eu^{3+}] = 18.06 mM).

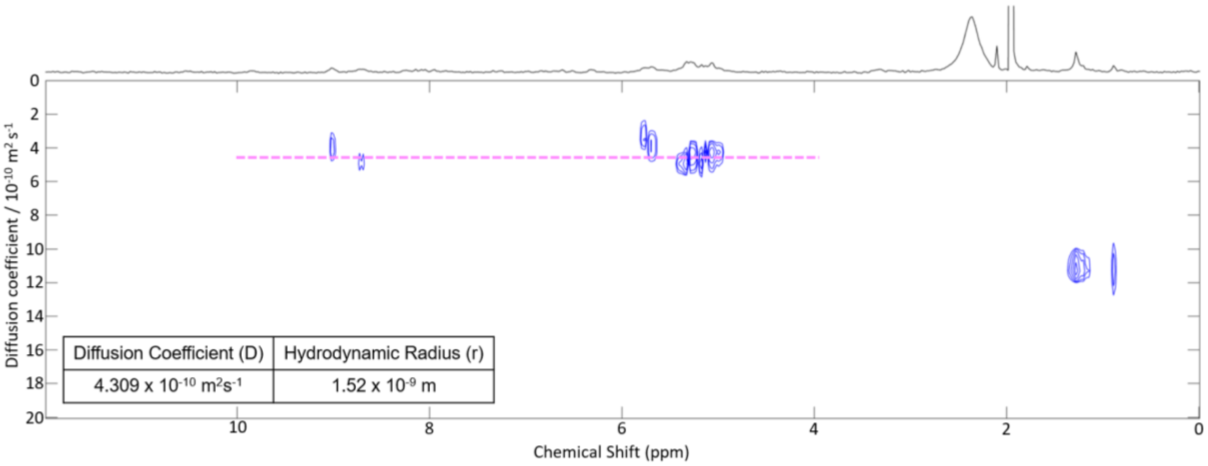

Figure S14. ^{1}H DOSY NMR spectrum (298 K, CD_3CN) of [**Eu-1**$(OTf)_{18}$] ([Eu^{3+}] = 6.02 mM).

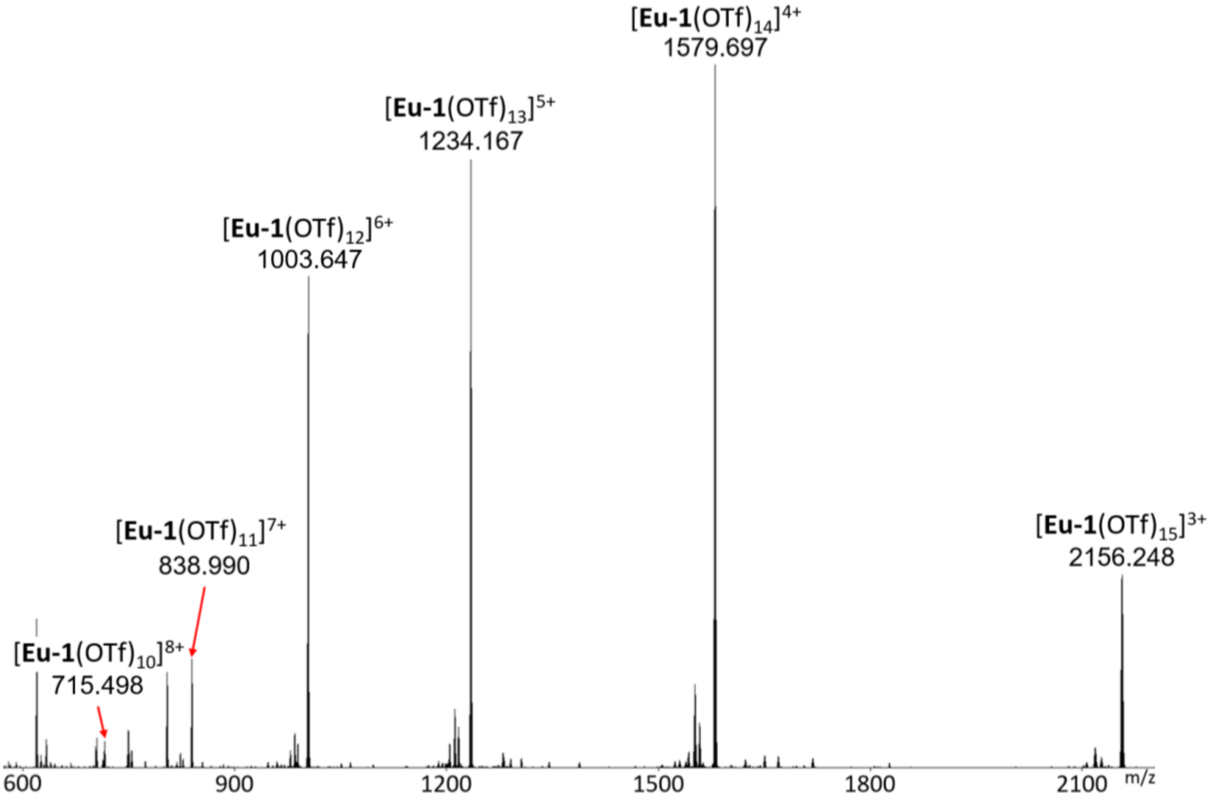

Figure S15. HR-ESI mass spectrum of the complexation products of **L1** and $Eu(OTf)_3$ in CD_3CN, highlighting the range of peaks corresponding to **Eu-1** species at different charges.

Table S1. Comparison between the observed and the simulated monoisotopic m/z peaks for different ions of **Eu-1**.

Species	Formula	Simulated m/z	Observed m/z
[**Eu-1**(OTf)$_{10}$]$^{8+}$	$[C_{184}H_{138}Eu_6F_{30}N_{30}O_{72}S_{10}]^{8+}$	715.500	715.498
[**Eu-1**(OTf)$_{11}$]$^{7+}$	$[C_{185}H_{138}Eu_6F_{33}N_{30}O_{75}S_{11}]^{7+}$	838.994	838.99
[**Eu-1**(OTf)$_{12}$]$^{6+}$	$[C_{186}H_{138}Eu_6F_{36}N_{30}O_{78}S_{12}]^{6+}$	1003.651	1003.647
[**Eu-1**(OTf)$_{13}$]$^{5+}$	$[C_{187}H_{138}Eu_6F_{39}N_{30}O_{81}S_{13}]^{5+}$	1234.172	1234.167
[**Eu-1**(OTf)$_{14}$]$^{4+}$	$[C_{188}H_{138}Eu_6F_{42}N_{30}O_{84}S_{14}]^{4+}$	1579.953	1579.947
[**Eu-1**(OTf)$_{15}$]$^{3+}$	$[C_{189}H_{138}Eu_6F_{45}N_{30}O_{87}S_{15}]^{3+}$	2156.255	2156.248

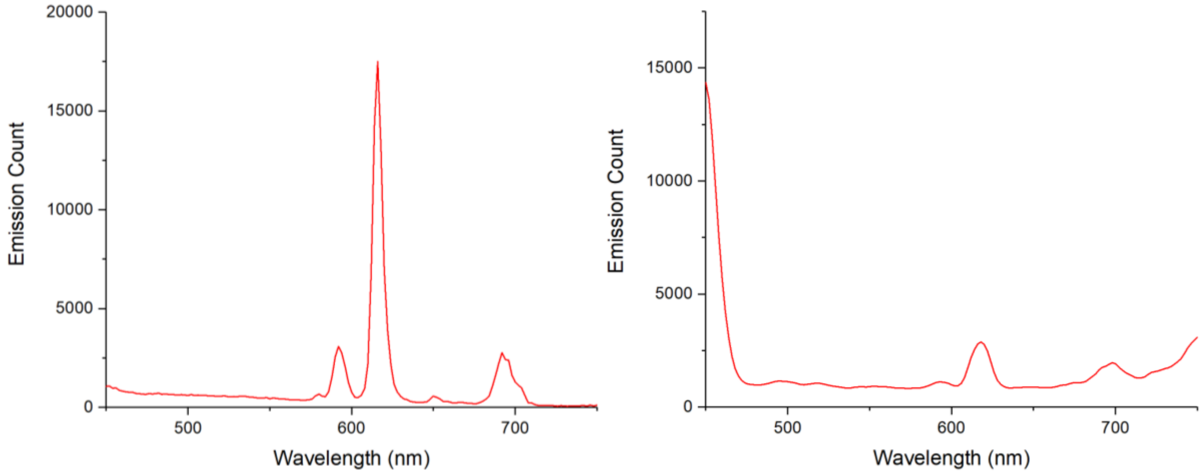

Fig S16. Corrected luminescence spectra of [**Eu-1**(OTf)$_{18}$], measured in CD_3CN, excited via the ligand at 330 nm (left) and direct intra f-f excitation at 397 nm (right).

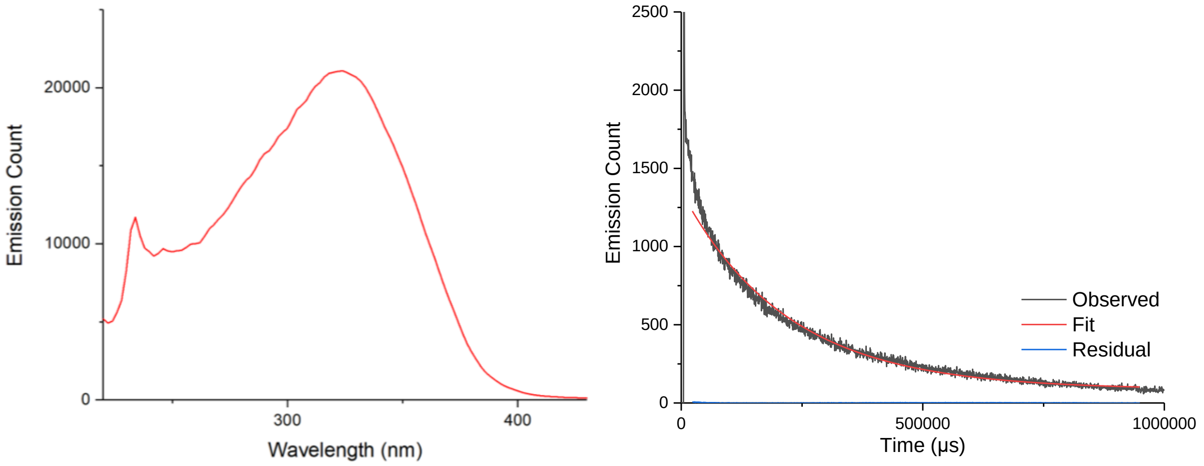

Figure S17. Corrected excitation spectrum of [**Eu-1**(OTf)$_{18}$] in CD_3CN measured at 617 nm ($^5D_0 \rightarrow {}^7F_2$) (left) and the fitted lifetime plot (λ_{exc} = 330 nm and λ_{em} = 617 nm) (right).

^{13}C-labelled MeCN Study. L1 (5.0 mg, 9.03 μmol, 1 eq.) and $Eu(OTf)_3$ (5.41 mg, 9.03 μmol, 1 eq.) were dissolved in CD_3CN (0.45 mL) and MeCN-^{13}C (0.05 mL), resulting in a pale-yellow solution, which was added to a J-Young NMR tube. The tube was sealed, and three vacuum/N_2 fill cycles were applied to degas the solution, before being heated (33 K, 24 hr).

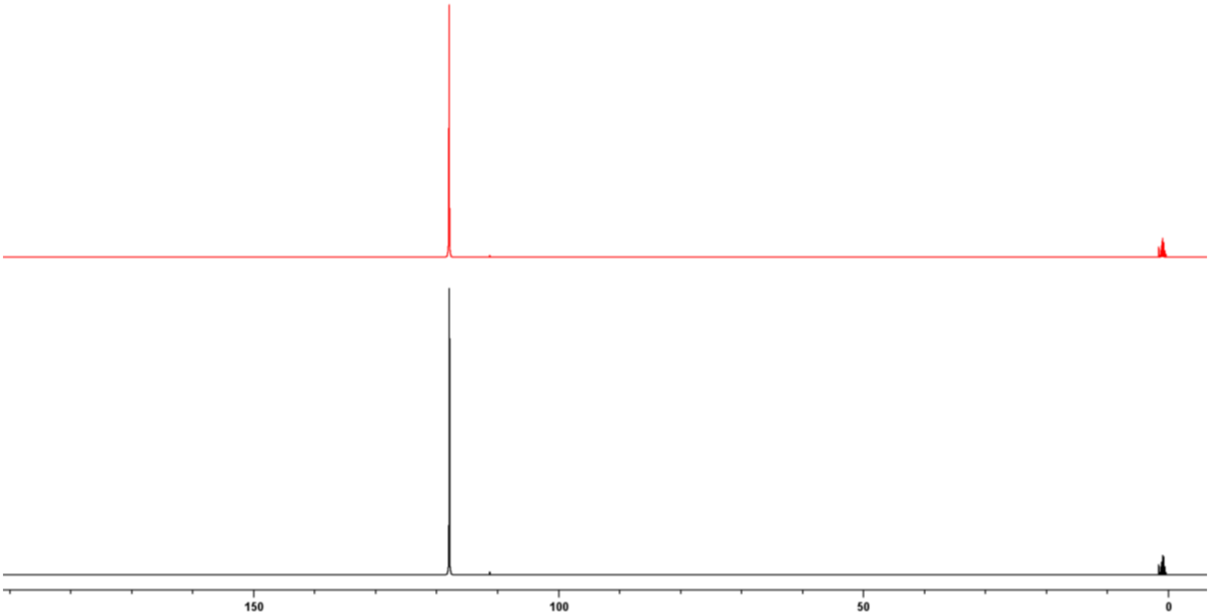

Figure S18. ^{13}C NMR spectra of **Eu-1**$(OTf)_{18}$ in 95:5 CD_3CN:MeCN-^{13}C solution (red), and 95:5 CD_3CN:MeCN-^{13}C solution only (black). No signals consistent with bound solvent were observable by ^{13}C NMR spectroscopy; we attribute this to signal broadening upon coordination to the paramagnetic Eu(III) centre.

18.06 mM Sm. L1 (5.0 mg, 9.03 µmol, 1 eq.) and $Sm(OTf)_3$ (5.38 mg, 9.03 µmol, 1 eq.) were dissolved in CD_3CN (0.5 mL), resulting in a pale-yellow solution, which was added to a J-Young NMR tube. The tube was sealed, and three vacuum/N_2 fill cycles were applied to degas the solution, before being heated (333 K, 24 hr). DOSY diffusion coefficient (CD_3CN, 298 K): 2.2 x 10^{-10} m^2s^{-1}. ^{19}F (470 MHz, 298 K, CD_3CN): -79.36 ppm. Accurate mass m/z: $[Sm_2$**L1**$_3(OTf)_3]^{3+}$ = 802.722, [**Sm-1**$(OTf)_{11}]^{7+}$ = 837.703, [**Sm-1**$(OTf)_{12}]^{6+}$ = 1001.979, [**Sm-1**$(OTf)_{13}]^{5+}$ = 1232.366, [**Sm-1**$(OTf)_{14}]^{4+}$ = 1577.695, [**Sm-1**$(OTf)_{15}]^{3+}$ = 2152.912.

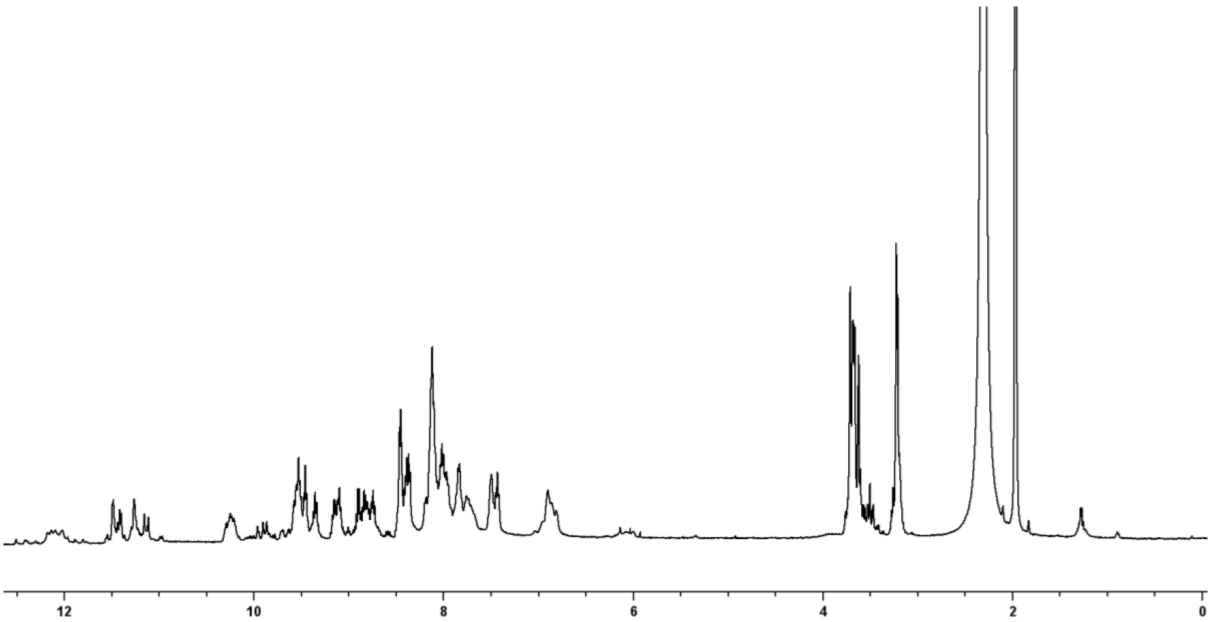

Figure S19. ^{1}H NMR spectrum (400 MHz, 293 K, CD_3CN) of [**Sm-1**$(OTf)_{18}$].

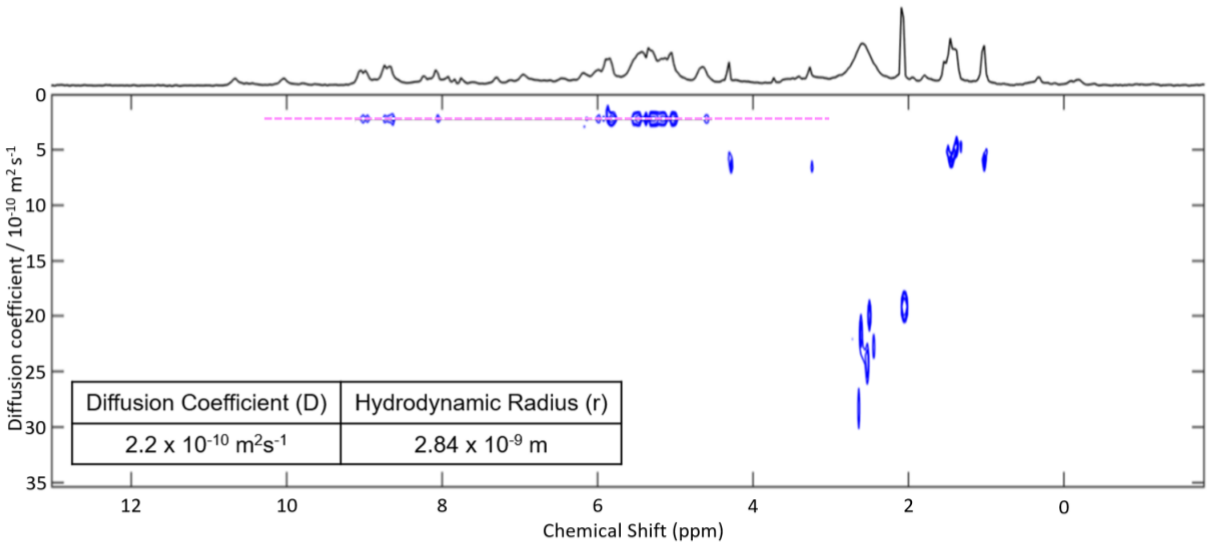

Figure S20. ^{1}H DOSY NMR spectrum (298 K, CD_3CN) of [**Sm-1**$(OTf)_{18}$] ([Sm^{3+}] = 18.06 mM).

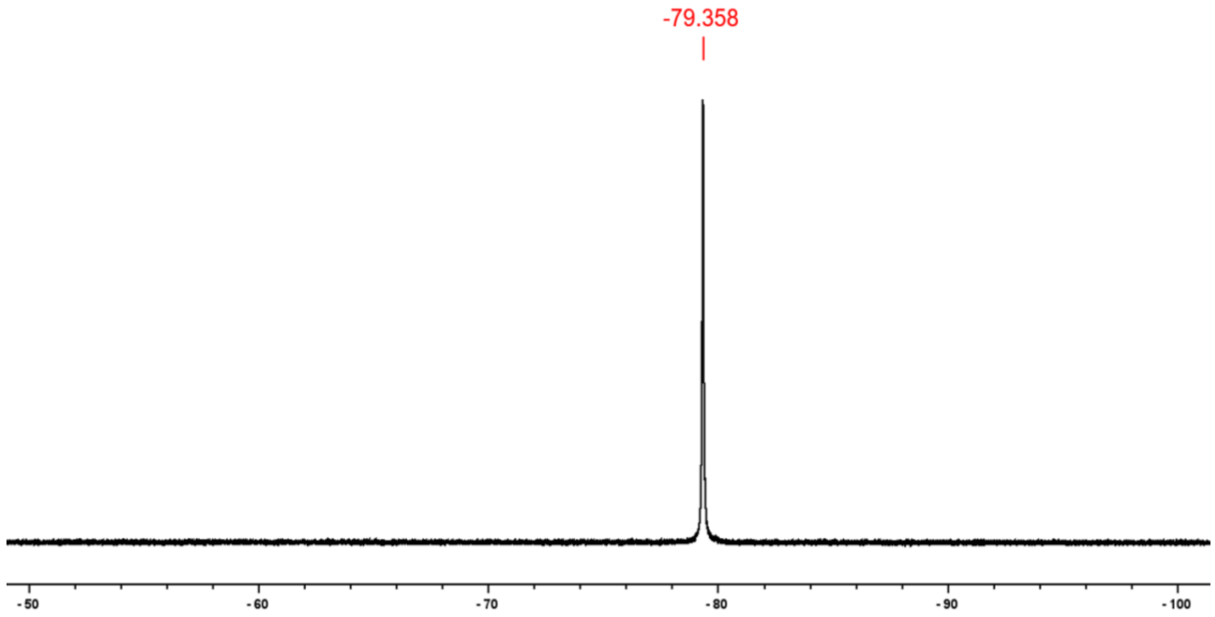

Figure S21. ^{19}F NMR spectrum (470 MHz, 298 K, CD_3CN) of [**Sm-1**$(OTf)_{18}$].

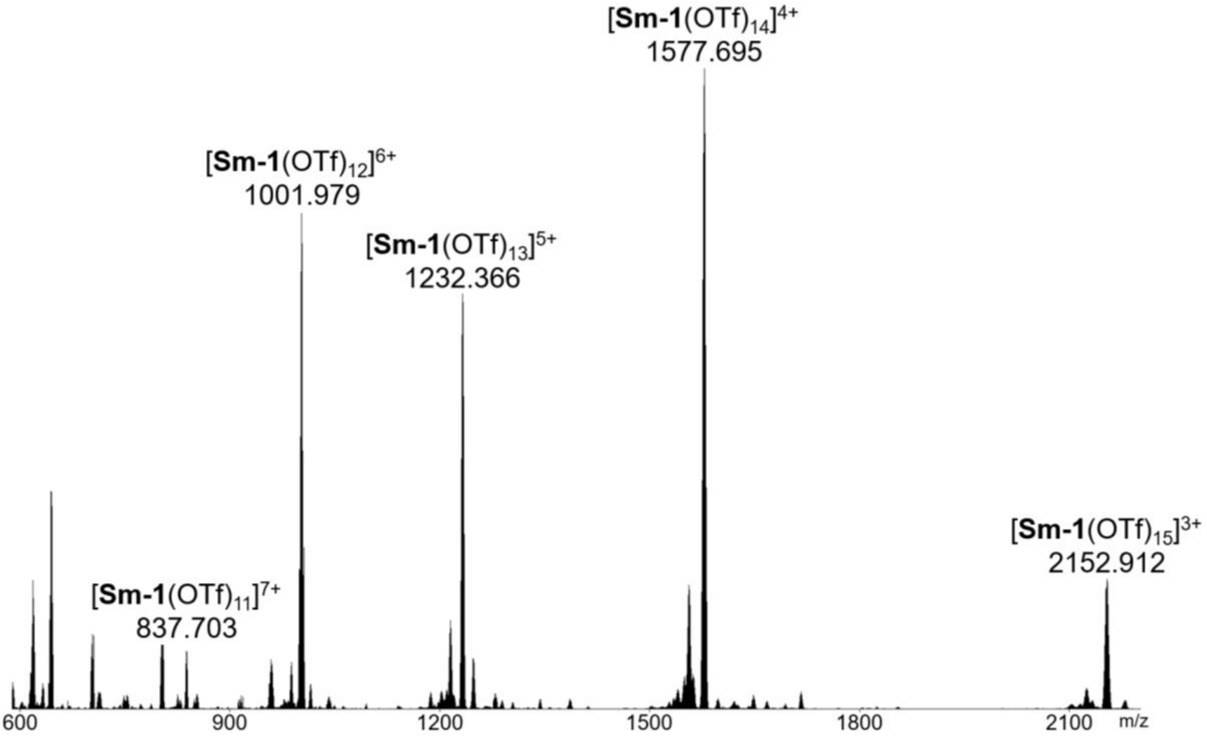

Figure S22. HR-ESI mass spectrum of the complexation products of **L1** and $Sm(OTf)_3$ in CD_3CN, highlighting the range of peaks corresponding to **Sm-1** species at different charges.

Table S2. Comparison between the observed and the simulated monoisotopic m/z peaks for different ions of **Sm-1**.

Species	Formula	Simulated m/z	Observed m/z
[**Sm-1**(OTf)$_{11}$]$^{7+}$	[$C_{185}H_{138}F_{33}N_{30}O_{75}S_{11}Sm_6$]$^{7+}$	837.706	837.703
[**Sm-1**(OTf)$_{12}$]$^{6+}$	[$C_{186}H_{138}F_{36}N_{30}O_{78}S_{12}Sm_6$]$^{6+}$	1001.982	1001.979
[**Sm-1**(OTf)$_{13}$]$^{5+}$	[$C_{187}H_{138}F_{39}N_{30}O_{81}S_{13}Sm_6$]$^{5+}$	1232.369	1232.366
[**Sm-1**(OTf)$_{14}$]$^{4+}$	[$C_{188}H_{138}F_{42}N_{30}O_{84}S_{14}Sm_6$]$^{4+}$	1577.700	1577.695
[**Sm-1**(OTf)$_{15}$]$^{3+}$	[$C_{189}H_{138}F_{45}N_{30}O_{87}S_{15}Sm_6$]$^{3+}$	2152.917	2152.912

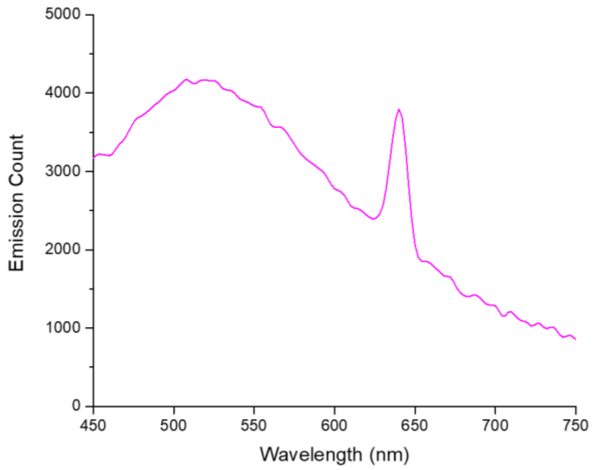

Fig S23. Luminescence spectrum of [**Sm-1**(OTf)$_{18}$] excited via the ligand at 330 nm in CD_3CN. Note that only the ($^4G_{5/2} \rightarrow {}^6H_{9/2}$) transition at 645 nm is observable above the residual ligand phosphorescence (broad feature).

18.06 mM Gd. L1 (5.0 mg, 9.03 µmol, 1 eq.) and $Gd(OTf)_3$ (5.46 mg, 9.03 µmol, 1 eq.) were dissolved in CD_3CN (0.5 mL), resulting in a pale-yellow solution, which was added to a J-Young NMR tube. The tube was sealed, and three vacuum/N_2 fill cycles were applied to degas the solution, before being heated (333 K, 24 hr). Accurate mass m/z: [**Gd-1**(OTf)$_{11}$]$^{7+}$ = 843.423, [**Gd-1**(OTf)$_{12}$]$^{6+}$ = 1008.985, [**Gd-1**(OTf)$_{13}$]$^{5+}$ = 1240.373, [**Gd-1**(OTf)$_{14}$]$^{4+}$ = 1587.704, [**Gd-1**(OTf)$_{15}$]$^{3+}$ = 2166.925.

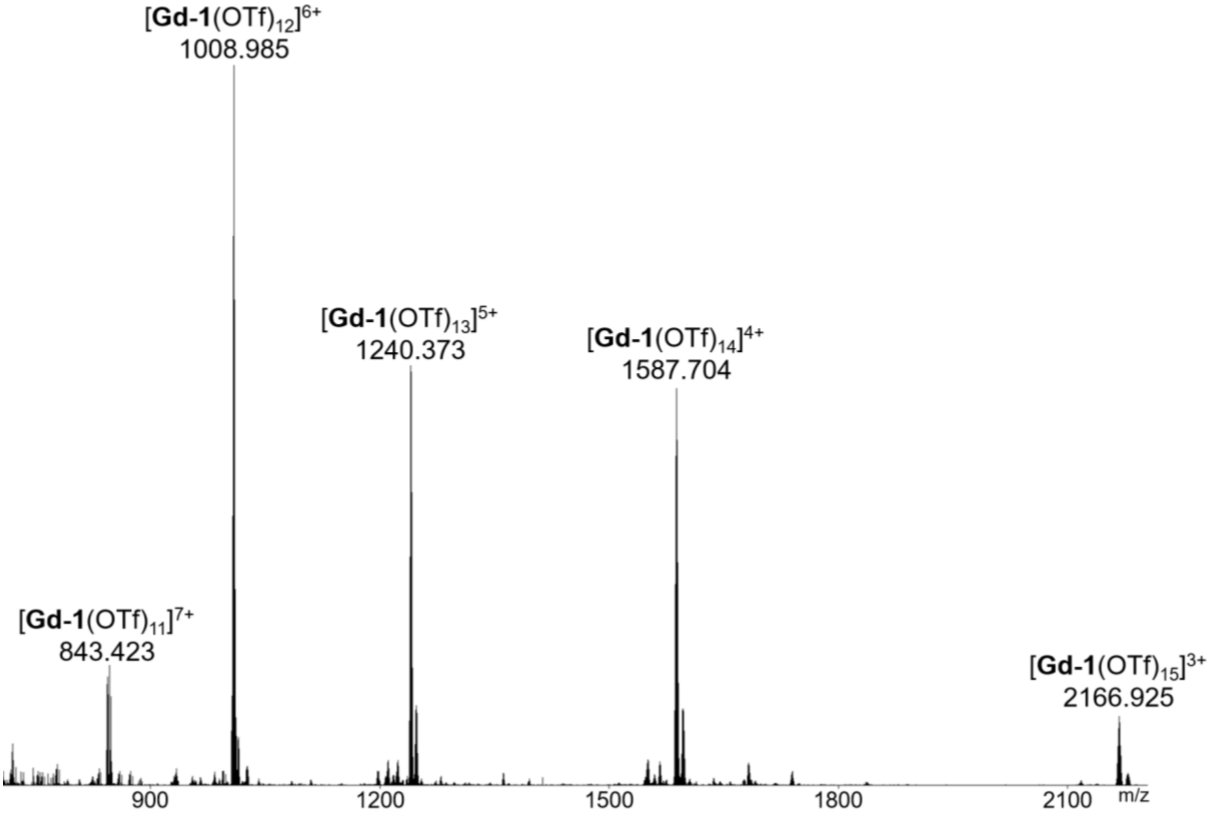

Figure S24. HR-ESI mass spectrum of the complexation products of **L1** and $Gd(OTf)_3$ in CD_3CN, highlighting the range of peaks corresponding to **Gd-1** species at different charges.

Table S3. Comparison between the observed and the simulated monoisotopic m/z peaks for different ions of **Gd-1**.

Species	Formula	Simulated m/z	Observed m/z
[**Gd-1**$(OTf)_{11}]^{7+}$	$[C_{185}H_{138}F_{33}Gd_6N_{30}O_{75}S_{11}]^{7+}$	843.425	843.423
[**Gd-1**$(OTf)_{12}]^{6+}$	$[C_{186}H_{138}F_{36}Gd_6N_{30}O_{78}S_{12}]^{6+}$	1008.988	1008.985
[**Gd-1**$(OTf)_{13}]^{5+}$	$[C_{187}H_{138}F_{39}Gd_6N_{30}O_{81}S_{13}]^{5+}$	1240.376	1240.373
[**Gd-1**$(OTf)_{14}]^{4+}$	$[C_{188}H_{138}F_{42}Gd_6N_{30}O_{84}S_{14}]^{4+}$	1587.708	1587.704
[**Gd-1**$(OTf)_{15}]^{3+}$	$[C_{189}H_{138}F_{45}Gd_6N_{30}O_{87}S_{15}]^{3+}$	2166.929	2166.925

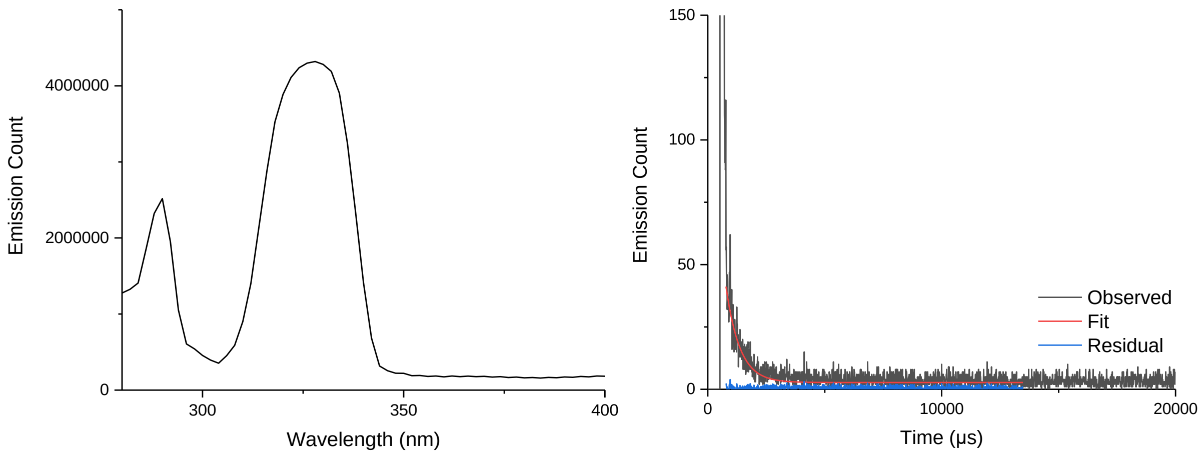

Figure S25. Corrected excitation spectrum of [**Gd-1**(OTf)$_{18}$] in CD_3CN at 77 K measured at 585 nm (left) and the fitted lifetime plot (λ_{exc} = 375 nm and λ_{em} = 617nm) (right).

18.06 mM Tb. **L1** (5.0 mg, 9.03 μmol, 1 eq.) and Tb(OTf)$_3$ (5.48 mg, 9.03 μmol, 1 eq.) were dissolved in CD_3CN (0.5 mL), resulting in a pale-yellow solution, which was added to a J-Young NMR tube. The tube was sealed, and three vacuum/N_2 fill cycles were applied to degas the solution, before being heated (333 K, 24 hr). Accurate mass m/z: [Tb$_2$**L1**$_3$(OTf)$_3$]$^{3+}$ = 803.399, [**Tb-1**(OTf)$_{12}$]$^{6+}$ = 1009.993, [**Tb-1**(OTf)$_{13}$]$^{5+}$ = 1242.383, [**Tb-1**(OTf)$_{14}$]$^{4+}$ = 1590.217, [**Tb-1**(OTf)$_{15}$]$^{3+}$ = 2169.945.

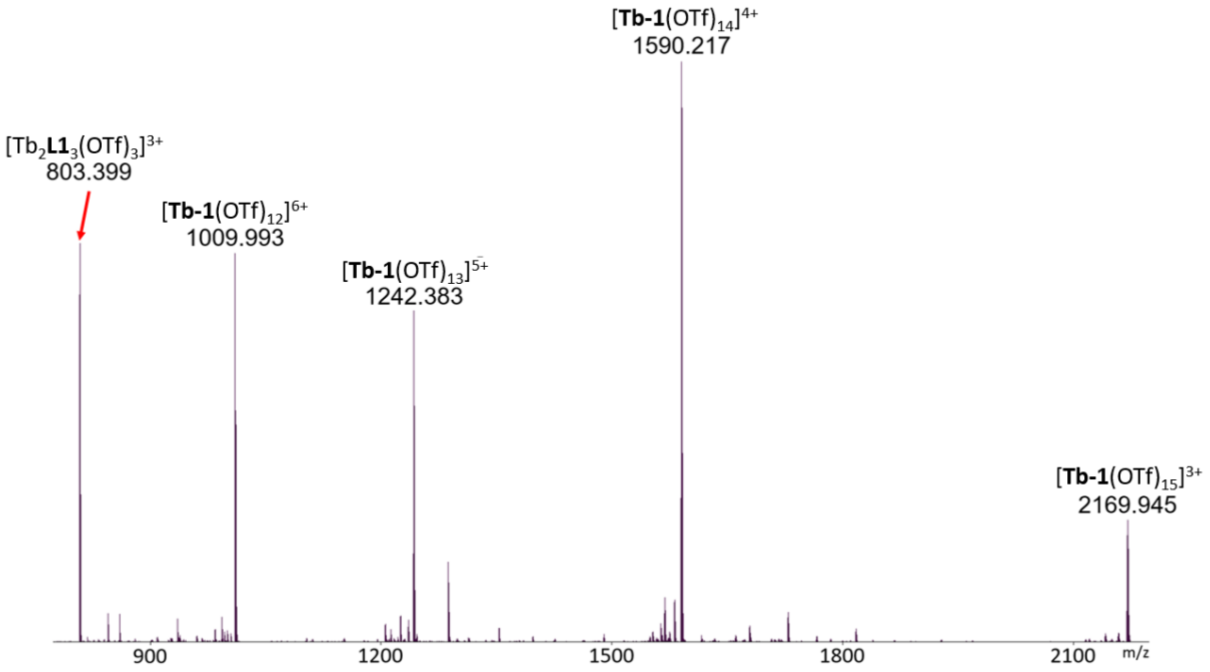

Figure S26. HR-ESI mass spectrum of the complexation products of **L1** and Tb(OTf)$_3$ in CD_3CN, highlighting the range of peaks corresponding to **Tb-1** species at different charges.

Table S4. Comparison between the observed and the simulated monoisotopic m/z peaks for different ions of **Tb-1**.

Species	Formula	Simulated m/z	Observed m/z
[**Tb-1**(OTf)$_{12}$]$^{6+}$	[$C_{186}H_{138}F_{36}N_{30}O_{78}S_{12}Tb_6$]$^{6+}$	1009.989	1009.993
[**Tb-1**(OTf)$_{13}$]$^{5+}$	[$C_{187}H_{138}F_{39}N_{30}O_{81}S_{13}Tb_6$]$^{5+}$	1242.378	1242.383
[**Tb-1**(OTf)$_{14}$]$^{4+}$	[$C_{188}H_{138}F_{42}N_{30}O_{84}S_{14}Tb_6$]$^{4+}$	1590.210	1590.217
[**Tb-1**(OTf)$_{15}$]$^{3+}$	[$C_{189}H_{138}F_{45}N_{30}O_{87}S_{15}Tb_6$]$^{3+}$	2169.938	2169.945

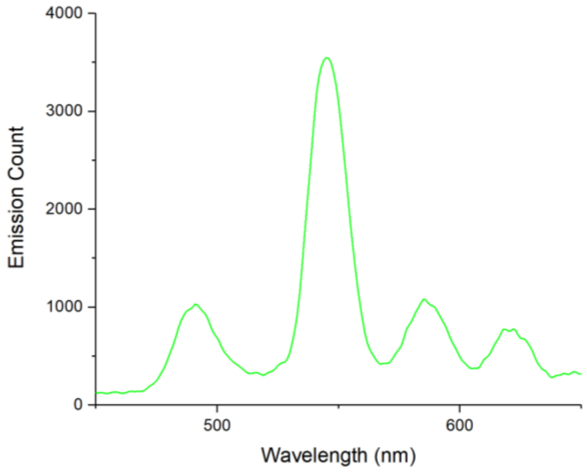

Fig S27. Corrected luminescence spectra of [**Tb-1**(OTf)$_{18}$], measured in CD_3CN, excited via the ligand at 330 nm.

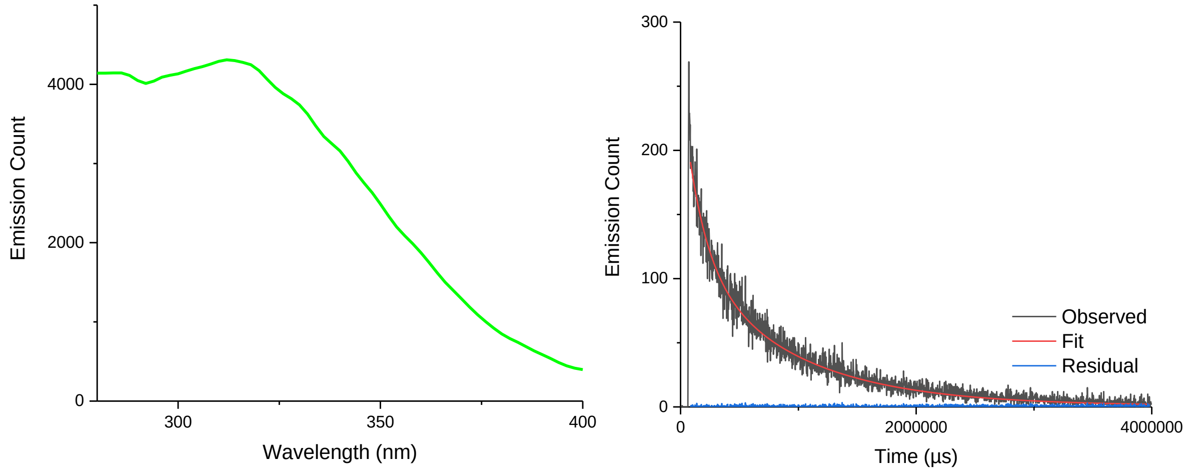

Figure S28. Corrected esxcitation spectrum of [**Tb-1**(OTf)$_{18}$] in CD_3CN measured at 545 nm ($^5D_4 \rightarrow {}^7F_5$) (left) and the fitted lifetime plot (λ_{exc} = 320 nm and λ_{em} = 545 nm) (right).

12.04 mM Lu. L1 (5.0 mg, 9.03 µmol, 3 eq.) and $Lu(OTf)_3$ (3.75 mg, 6.02 µmol, 2 eq.) were dissolved in CD_3CN (0.5 mL), resulting in a pale-yellow solution, which was added to a J-Young NMR tube. The tube was sealed, and three vacuum/N_2 fill cycles were applied to degas

the solution, before being heated (333 K, 24 hr). ^{1}H (500 MHz, 298 K, CD_3CN): 4.03 (m, Int = 3), 7.54 (m, Int = 4), 8.66 (m, Int = 3), 11.00 (m, Int = 1) ppm. DOSY diffusion coefficient (CD_3CN, 298 K): 3.61 x 10^{-10} m^2s^{-1} and 4.23 x 10^{-10}. Accurate mass m/z: $[Lu_2\mathbf{L1}_3]^{6+}$ = 335.059, $[Lu_2\mathbf{L1}_2(\mathbf{L1}^-)]^{5+}$ = 401.868, $[Lu_2\mathbf{L1}_3(OTf)]^{5+}$ = 431.861, $[Lu_2\mathbf{L1}_3(OTf)_2]^{4+}$ = 577.065, $[Lu_2\mathbf{L1}_3(\mathbf{L1}^-)(OTf)_2]^{3+}$ = 769.084, $[Lu_2\mathbf{L1}_3(OTf)_3]^{3+}$ = 819.070, $[Lu_4\mathbf{L1}_6(OTf)_7]^{5+}$ = 1012.874, $[Lu_2\mathbf{L1}_3(OTf)_4]^{2+}$ = 1303.081, $[Lu_4\mathbf{L1}_6(OTf)_8]^{4+}$ = 1303.081, $[Lu_4\mathbf{L1}_6(OTf)_9]^{3+}$ = 1787.426.

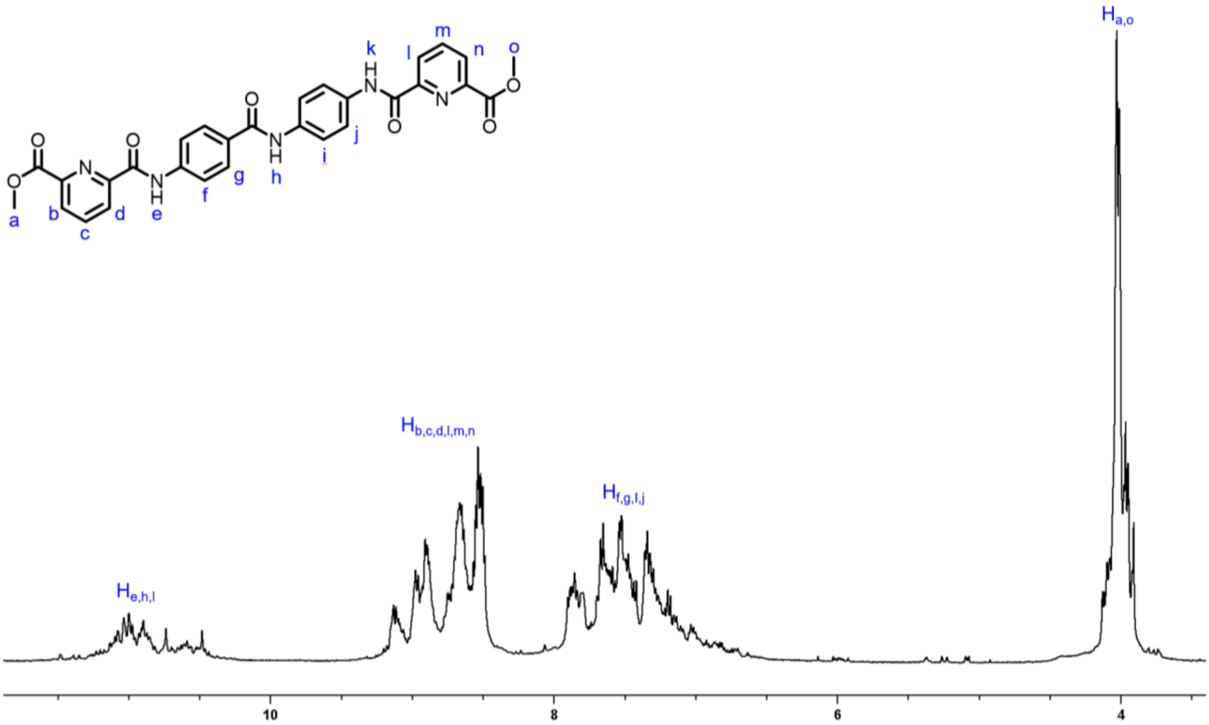

Figure S29. ^{1}H NMR spectrum (500 MHz, 298 K, CD_3CN) of the complexation products of **L1** and $Lu(OTf)_3$.

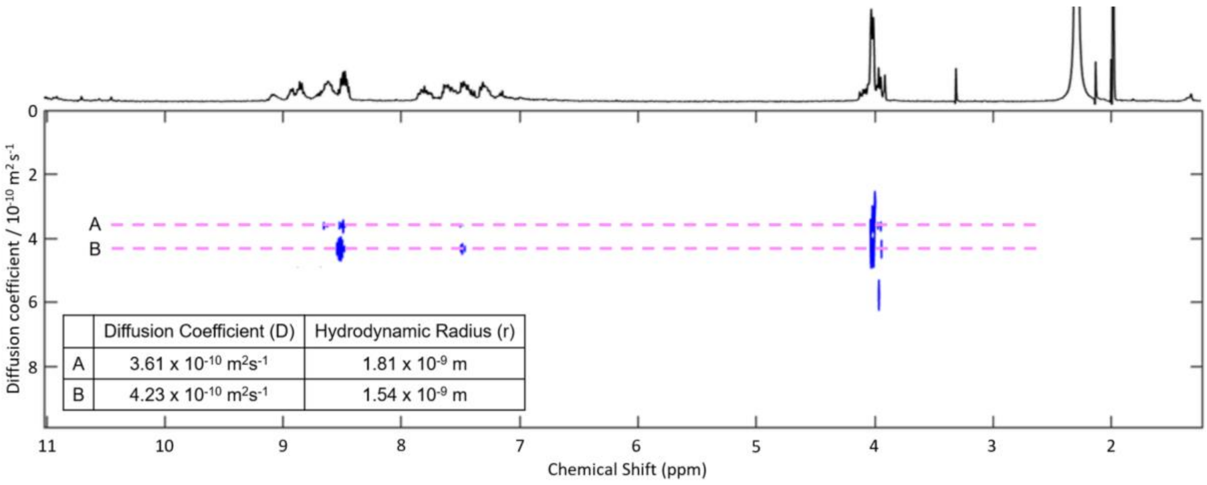

Figure S30. ^{1}H DOSY NMR spectrum (298 K, CD_3CN) of the complexation products of **L1** and $Lu(OTf)_3$ ($[Lu^{3+}]$ = 0.012 M).

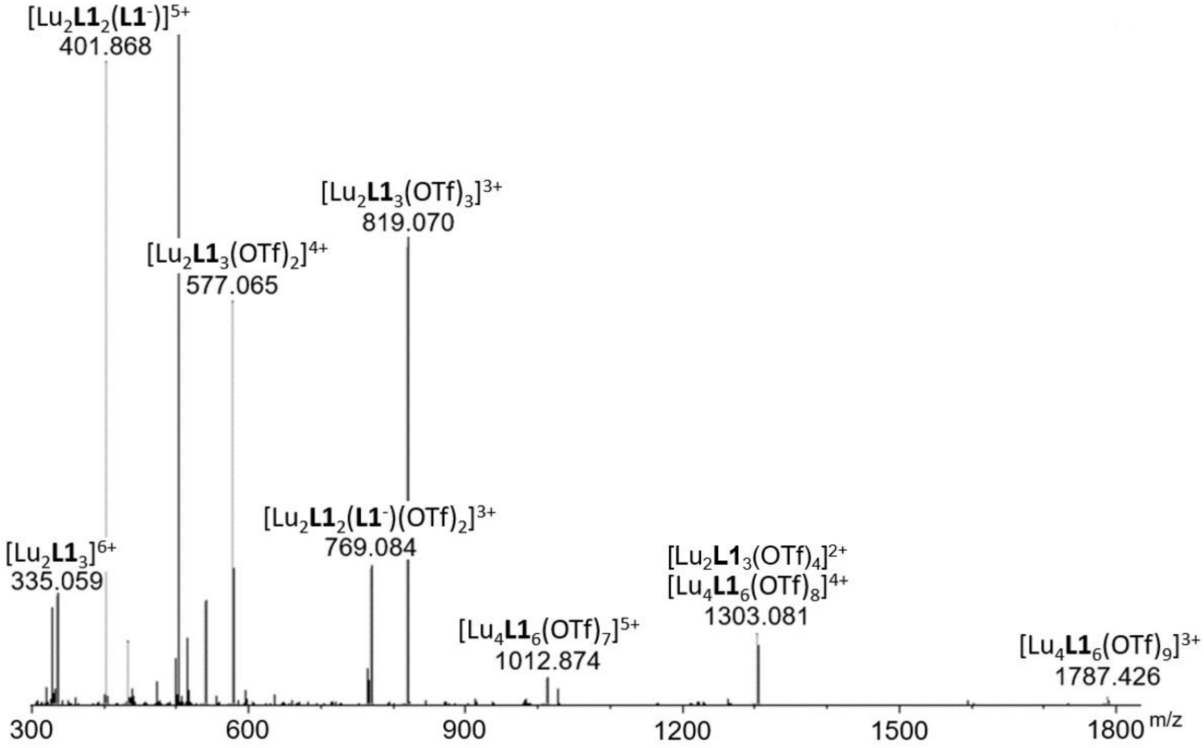

Figure S31. HR-ESI mass spectrum of the complexation products of **L1** and $Lu(OTf)_3$ in CD_3CN, highlighting peaks corresponding to Lu.**L1** complexation species.

Table S5. Comparison between the observed and the simulated monoisotopic m/z peaks for complexation products of **L1** and Lu(OTf)$_3$.

Species	Formula	Simulated m/z	Observed m/z
[Lu$_2$**L1**$_3$]$^{6+}$	[$C_{87}H_{69}Lu_2N_{15}O_{21}$]$^{6+}$	335.0601	335.0587
[Lu$_2$**L1**$_3$(OTf)$_2$]$^{4+}$	[$C_{89}H_{69}F_6Lu_2N_{15}O_{27}S_2$]$^{4+}$	577.0664	577.0646
[Lu$_2$**L1**$_3$(OTf)$_3$]$^{3+}$	[$C_{90}H_{69}F_9Lu_2N_{15}O_{30}S_3$]$^{3+}$	819.0727	819.0699
[Lu$_2$**L1**$_3$(OTf)$_4$]$^{2+}$	[$C_{91}H_{69}F_{12}Lu_2N_{15}O_{33}S_4$]$^{2+}$	1303.0850	1303.0813
[Lu$_4$**L1**$_6$(OTf)$_7$]$^{5+}$	[$C_{181}H_{138}F_{21}Lu_4N_{30}O_{63}S_7$]$^{5+}$	1012.6775	1012.6743
[Lu$_4$**L1**$_6$(OTf)$_8$]$^{4+}$	[$C_{182}H_{138}F_{24}Lu_4N_{30}O_{66}S_8$]$^{4+}$	1303.0850	1303.0813
[Lu$_4$**L1**$_6$(OTf)$_9$]$^{3+}$	[$C_{183}H_{138}F_{27}Lu_4N_{30}O_{69}S_9$]$^{3+}$	1787.4313	1787.4263
[Lu$_2$**L1**$_2$(**L1**$^-$)]$^{5+}$	[$C_{87}H_{68}Lu_2N_{15}O_{21}$]$^{5+}$	401.8706	401.8681
[Lu$_2$**L1**$_2$(**L1**$^-$)(OTf)$_2$]$^{3+}$	[$C_{89}H_{68}F_6Lu_2N_{15}O_{27}S_2$]$^{3+}$	769.0861	769.0838

12.04 mM Yb. L1 (5.0 mg, 9.03 μmol, 3 eq.) and Yb(OTf)$_3$ (3.73 mg, 6.02 μmol, 2 eq.) were dissolved in CD_3CN (0.5 mL), resulting in a pale-yellow solution, which was added to a J-Young NMR tube. The tube was sealed, and three vacuum/N_2 fill cycles were applied to degas the solution, before being heated (333 K, 24 hr). ^{19}F (470 MHz, 298 K, CD_3CN): -79.08 ppm. Accurate mass m/z: [Yb$_2$**L1**$_3$(OTf)$_2$]$^{4+}$ = 576.064, [Yb$_2$**L1**$_2$(**L1**$^-$)(OTf)$_2$]$^{3+}$ = 768.083, [Yb$_2$**L1**$_3$(OTf)$_3$]$^{3+}$ = 818.069, [Yb$_4$**L1**$_6$(OTf)$_6$]$^{6+}$ = 818.069 [Yb$_4$**L1**$_6$(OTf)$_7$]$^{5+}$ = 1011.274, [**Yb-1**(OTf)$_{12}$]$^{6+}$ = 1024.999, [**Yb-1**(OTf)$_{13}$]$^{5+}$ = 1259.598, [Yb$_2$**L1**$_3$(OTf)$_4$]$^{2+}$ = 1301.581, [Yb$_4$**L1**$_6$(OTf)$_8$]$^{4+}$ = 1301.581, [**Yb-1**(OTf)$_{14}$]$^{4+}$ = 1611.474, [Yb$_4$**L1**$_6$(OTf)$_9$]$^{3+}$ = 1785.092, [**Yb-1**(OTf)$_{15}$]$^{3+}$ = 2198.285.

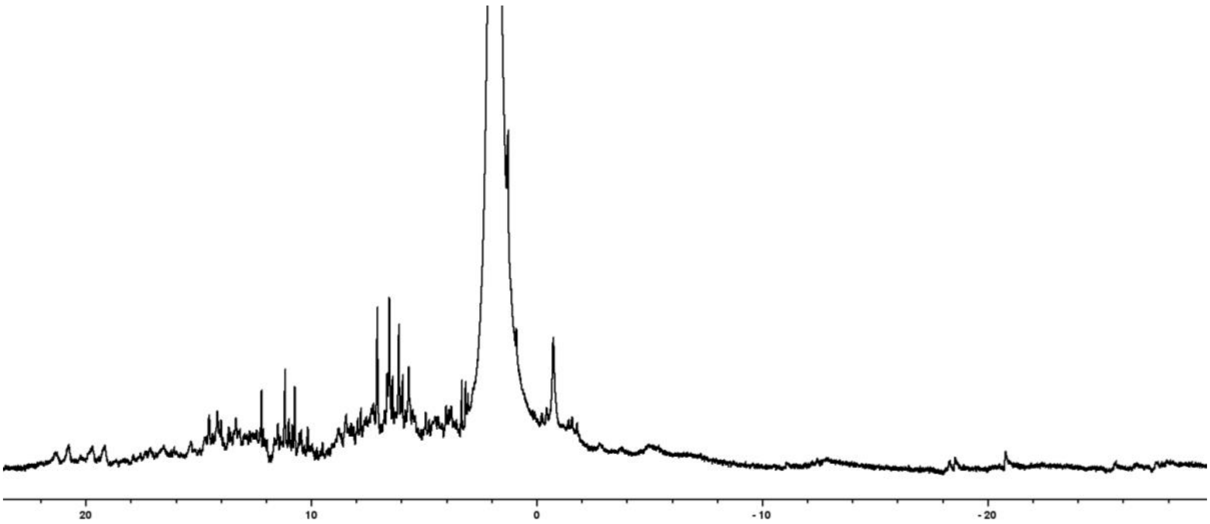

Figure S32. ^{1}H NMR spectrum (500 MHz, 298 K, CD_3CN) of the complexation products of **L1** and Yb(OTf)$_3$.

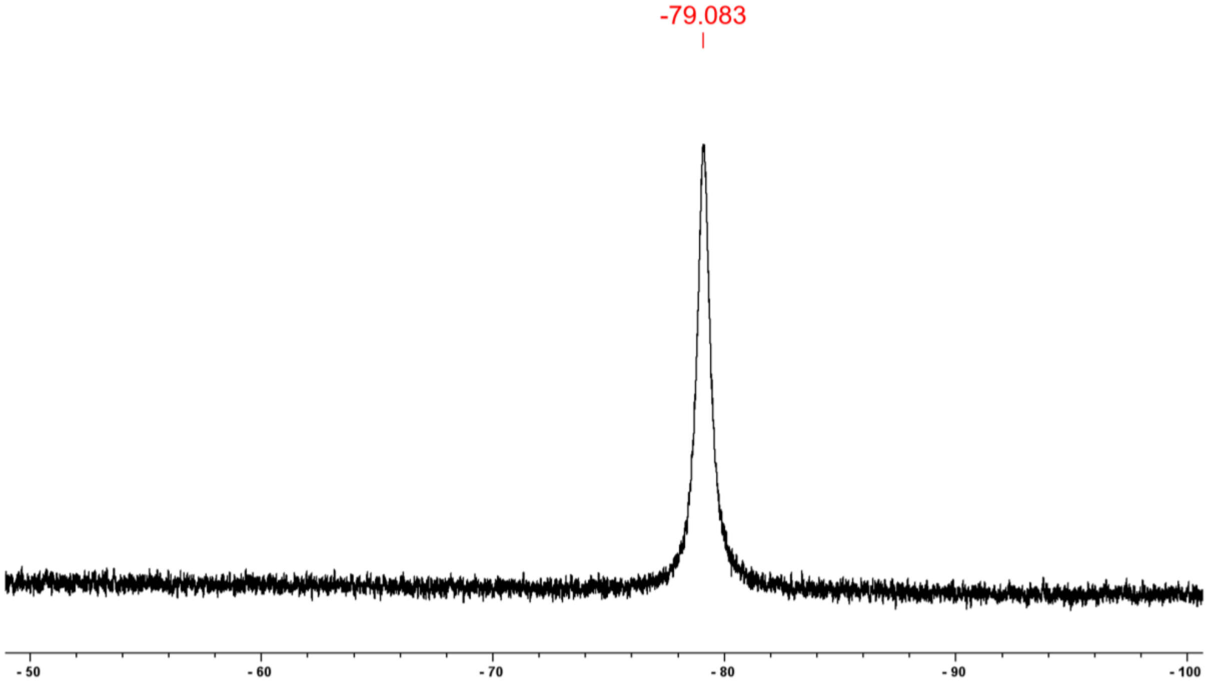

Figure S33. ^{19}F NMR spectrum (470 MHz, 298 K, CD_3CN) of the complexation of **L1** with $Yb(OTf)_3$.

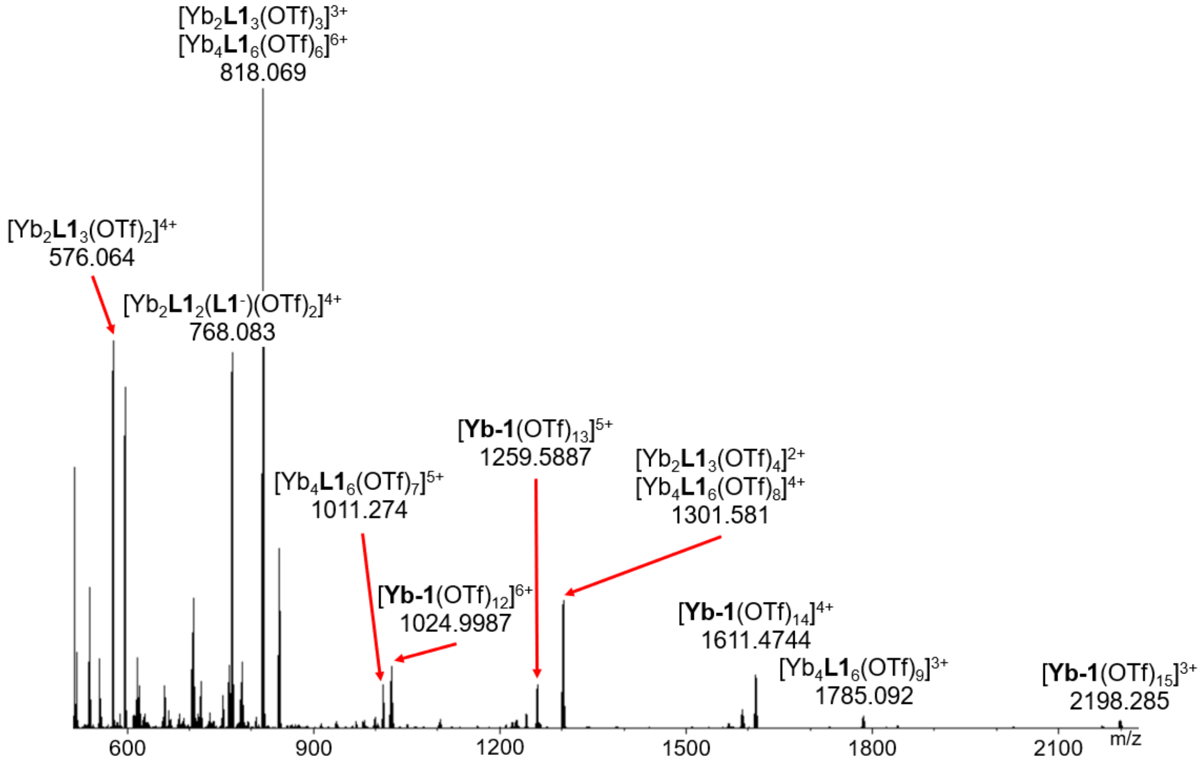

Figure S34. HR-ESI mass spectrum of the complexation products of **L1** and $Yb(OTf)_3$ in CD_3CN, highlighting peaks corresponding to Yb.**L1** complexation species.

Table S6. Comparison between the observed and the simulated monoisotopic m/z peaks for complexation products of **L1** and $Yb(OTf)_3$.

Species	Formula	Simulated m/z	Observed m/z
$[Yb_2\mathbf{L1}_3(OTf)_2]^{4+}$	$[C_{89}H_{69}F_6N_{15}O_{27}S_2Yb_2]^{4+}$	576.0649	576.064
$[Yb_2\mathbf{L1}_3(OTf)_3]^{3+}$	$[C_{90}H_{69}F_9N_{15}O_{30}S_3Yb_2]^{3+}$	818.071	818.069
$[Yb_2\mathbf{L1}_3(OTf)_4]^{2+}$	$[C_{91}H_{69}F_{12}N_{15}O_{33}S_4Yb_2]^{2+}$	1301.582	1301.581
$[Yb_4\mathbf{L1}_6(OTf)_6]^{6+}$	$[C_{180}H_{138}F_{18}N_{30}O_{60}S_6Yb_4]^{6+}$	818.071	818.069
$[Yb_4\mathbf{L1}_6(OTf)_7]^{5+}$	$[C_{181}H_{138}F_{21}N_{30}O_{63}S_7Yb_4]^{5+}$	1011.2755	1011.274
$[Yb_4\mathbf{L1}_6(OTf)_8]^{4+}$	$[C_{182}H_{138}F_{24}N_{30}O_{66}S_8Yb_4]^{4+}$	1301.582	1301.581
$[Yb_4\mathbf{L1}_6(OTf)_9]^{3+}$	$[C_{183}H_{138}F_{27}N_{30}O_{69}S_9Yb_4]^{3+}$	1785.0935	1785.092
$[\mathbf{Yb\text{-}1}(OTf)_{12}]^{6+}$	$[C_{186}H_{138}F_{36}N_{30}O_{78}S_{12}Yb_6]^{6+}$	1025.0021	1024.9987
$[\mathbf{Yb\text{-}1}(OTf)_{13}]^{5+}$	$[C_{187}H_{138}F_{39}N_{30}O_{81}S_{13}Yb_6]^{5+}$	1259.5934	1259.598
$[\mathbf{Yb\text{-}1}(OTf)_{14}]^{4+}$	$[C_{188}H_{138}F_{42}N_{30}O_{84}S_{14}Yb_6]^{4+}$	1611.4795	1611.4744
$[\mathbf{Yb\text{-}1}(OTf)_{15}]^{3+}$	$[C_{189}H_{138}F_{45}N_{30}O_{87}S_{15}Yb_6]^{3+}$	2198.2902	2198.285
$[Yb_2\mathbf{L1}_2(\mathbf{L1}^-)(OTf)_2]^{3+}$	$[C_{89}H_{68}F_6N_{15}O_{27}S_2Yb_2]^{3+}$	768.084	768.083

16.78 mM Y(2:3 Y: L1 stoichiometry). L1 (6.8 mg, 12.28 μmol, 3 eq.) and $Y(OTf)_3$ (4.5 mg, 8.39 μmol, 2 eq.) were dissolved in CD_3CN (0.5 mL), resulting in a pale-yellow solution, which was added to a J-Young NMR tube. The tube was sealed, and three vacuum/N_2 fill cycles were applied to degas the solution, before being heated (333 K, 24 hr).

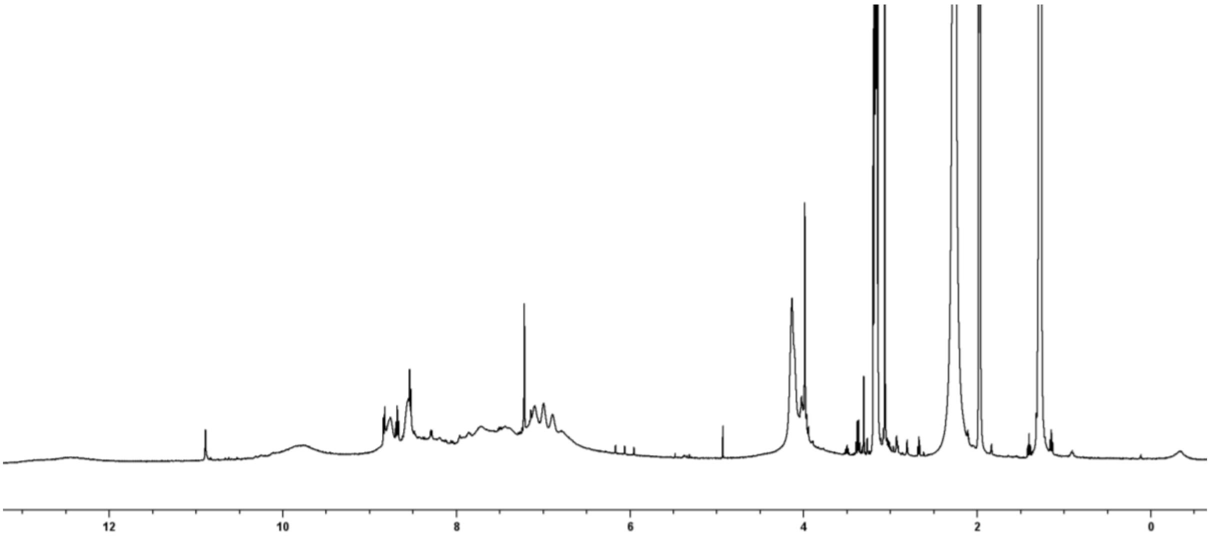

Figure S35. ^{1}H NMR spectrum (500 MHz, 298 K, CD_3CN) of the complexation products of **L1** and $Y(OTf)_3$ in a 2:3 Y: **L1** stoichiometry.

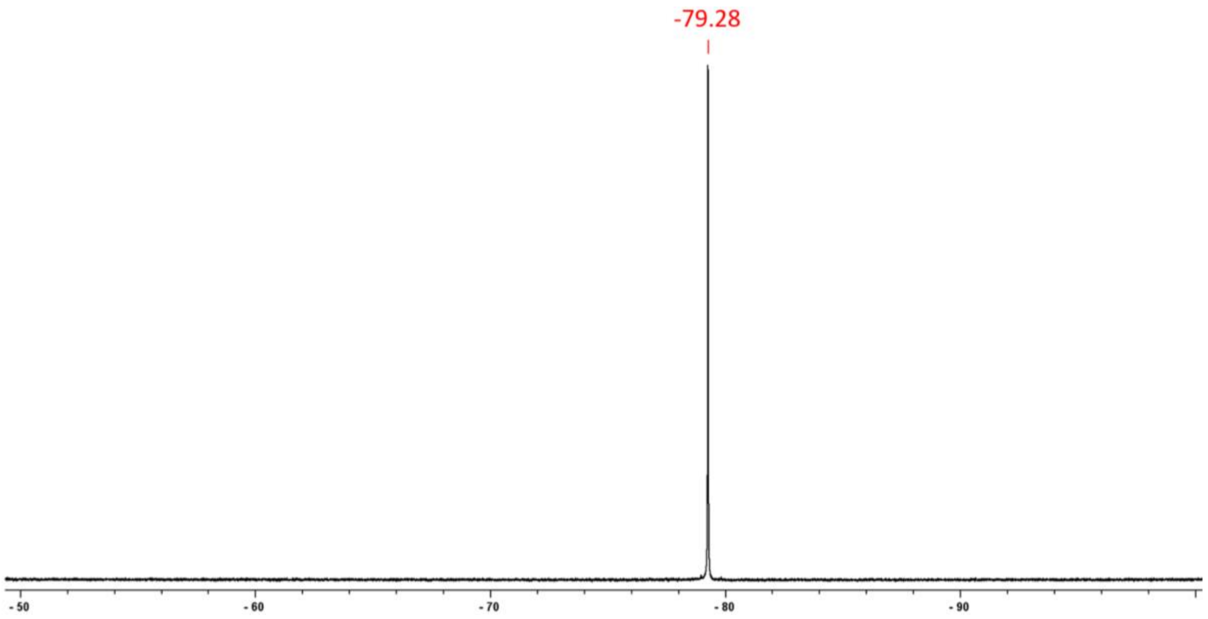

Figure S36. ^{19}F NMR spectrum (470 MHz, 298 K, CD_3CN) of the complexation of **L1** with $Y(OTf)_3$ in a 2:3 Y: **L1** stoichiometry.

18.06 mM Y. (1:1 Y: L1 stoichiometry). L1 (5.0 mg, 9.03.µmol, 1 eq.) and $Y(OTf)_3$ (5.0 mg, 9.03 µmol, 1 eq.) were dissolved in CD_3CN (0.5 mL), resulting in a pale-yellow solution, which was added to a J-Young NMR tube. The tube was sealed, and three vacuum/N_2 fill cycles were applied to degas the solution, before being heated (333 K, 24 hr).

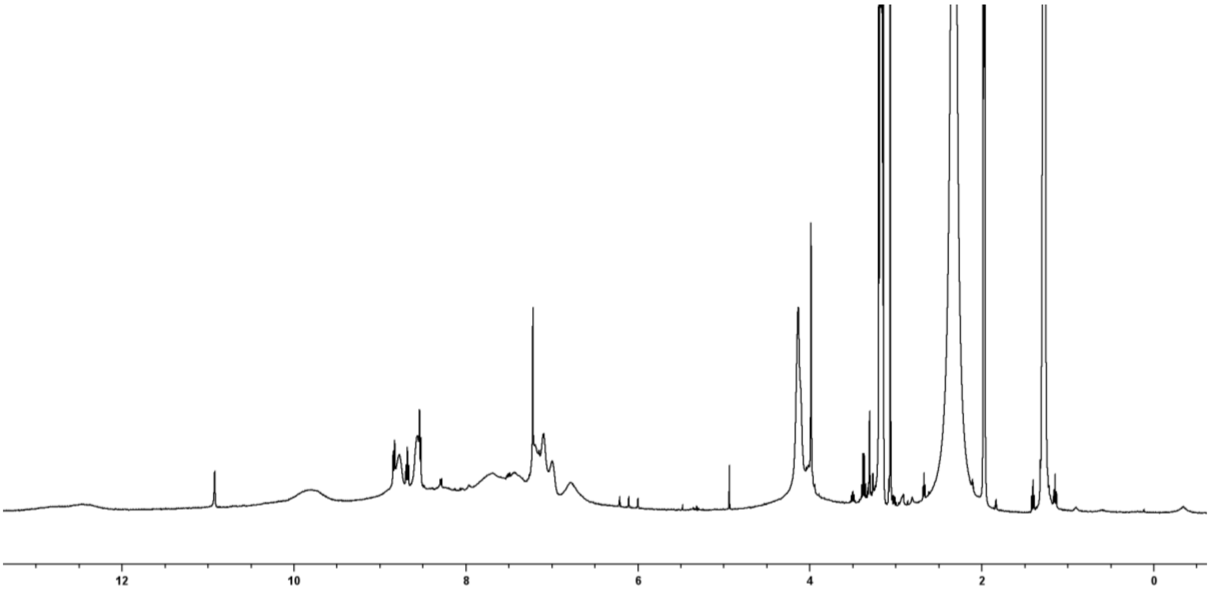

Figure S37. ^{1}H NMR spectrum (500 MHz, 298 K, CD_3CN) of the complexation products of **L1** and $Y(OTf)_3$ in a 1:1 Y: **L1** stoichiometry.

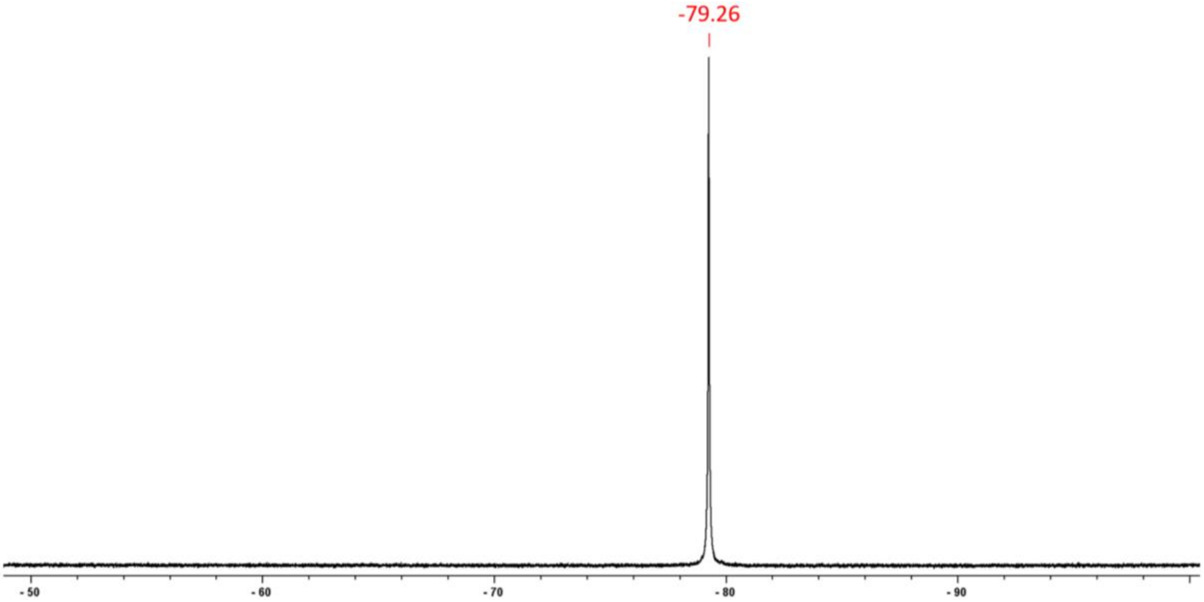

Figure S38. ^{19}F NMR spectrum (470 MHz, 298 K, CD_3CN) of the complexation of **L1** with $Y(OTf)_3$ in a 1:1 Y: **L1** stoichiometry.

S1.4. Self-Assembly Reactions with Symmetrical Ligand L2

12.38 mM Eu, 3:2 L2:Eu stoichiometry. L2 (5.0 mg, 9.28 µmol, 3 eq.) and $Eu(OTf)_3$ (3.71 mg, 6.19 µmol, 2 eq.) were dissolved in CD_3CN (0.5 mL), resulting in a pale-yellow solution, which was added to a J-Young NMR tube. The tube was sealed, and three vacuum/N_2 fill cycles were applied to degas the solution, before being heated (333 K, 24 hr). ^{19}F (470 MHz, 298 K, CD_3CN): -79.27 ppm. Accurate mass m/z: $[Eu_2\mathbf{L2}_3(OTf)]^{5+}$ = 413.468, $[Eu\mathbf{L2}(OTf)]^{2+}$ = 420.027, $[\mathbf{L2}+H]^{+}$ = 539.190, $[Eu_2\mathbf{L2}_3(OTf)_2]^{4+}$ = 554.073, $[Eu\mathbf{L2}_2(OTf)]^{2+}$ = 689.119, $[Eu_2\mathbf{L2}_3(OTf)_3]^{3+}$ = 789.081, $[Eu\mathbf{L2}(OTf)_2]^{+}$ = 989.005, $[Eu_2\mathbf{L2}_2(OTf)_2]^{2+}$ = 989.005, $[Eu_2\mathbf{L2}_3(OTf)_4]^{2+}$ = 1258.098, $[Eu\mathbf{L2}_2(OTf)_2]^{+}$ = 1527.190.

18.56 mM Eu, 1:1 L2:Eu stoichiometry. L2 (5.0 mg, 9.28 µmol, 1 eq.) and $Eu(OTf)_3$ (5.56 mg, 9.28 µmol, 1 eq.) were dissolved in CD_3CN (0.5 mL), resulting in a pale-yellow solution, which was added to a J-Young NMR tube. The tube was sealed, and three vacuum/N_2 fill cycles were applied to degas the solution, before being heated (333 K, 24 hr). Accurate mass m/z: $[Eu\mathbf{L2}(OTf)]^{2+}$ = 420.027, $[Eu\mathbf{L2}(OTf)_2]^{+}$ = 989.005, $[Eu_2\mathbf{L2}_2(OTf)_2]^{2+}$ = 989.005.

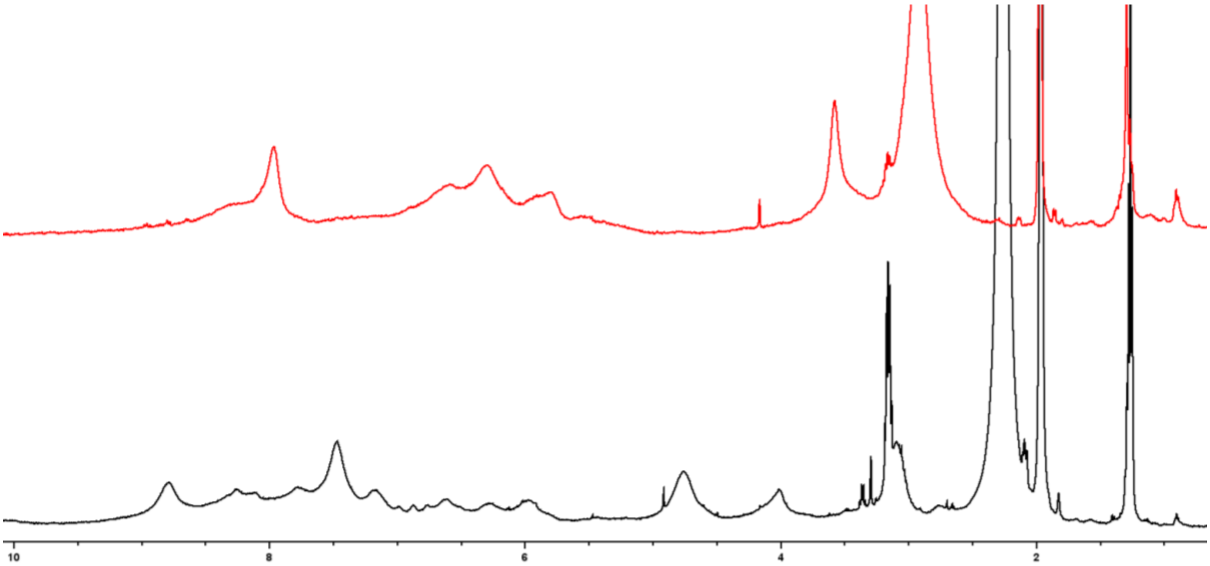

Figure S39. ^{1}H NMR spectra (400 MHz, 298 K, CD_3CN) of the complexation products of $Eu(OTf)_3$ and **L2** at different stoichiometries (2:3 = black, 1:1 = red).

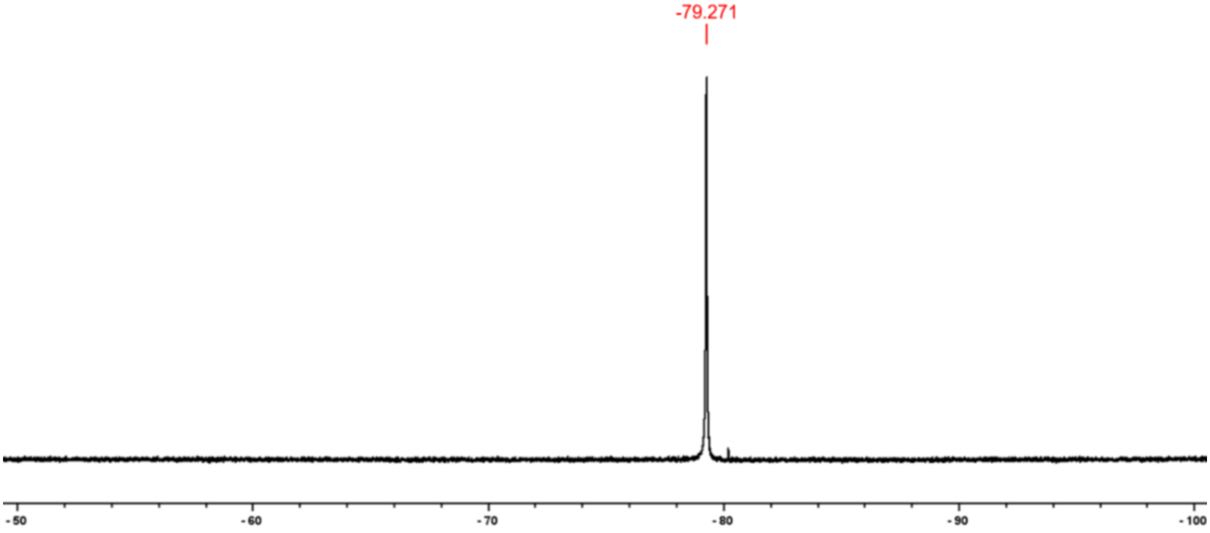

Figure S40. ^{19}F NMR spectrum (470 MHz, 298 K, CD_3CN) of the complexation products of $Eu(OTf)_3$ and **L2** in a 3:2 **L2**:Eu stoichiometry.

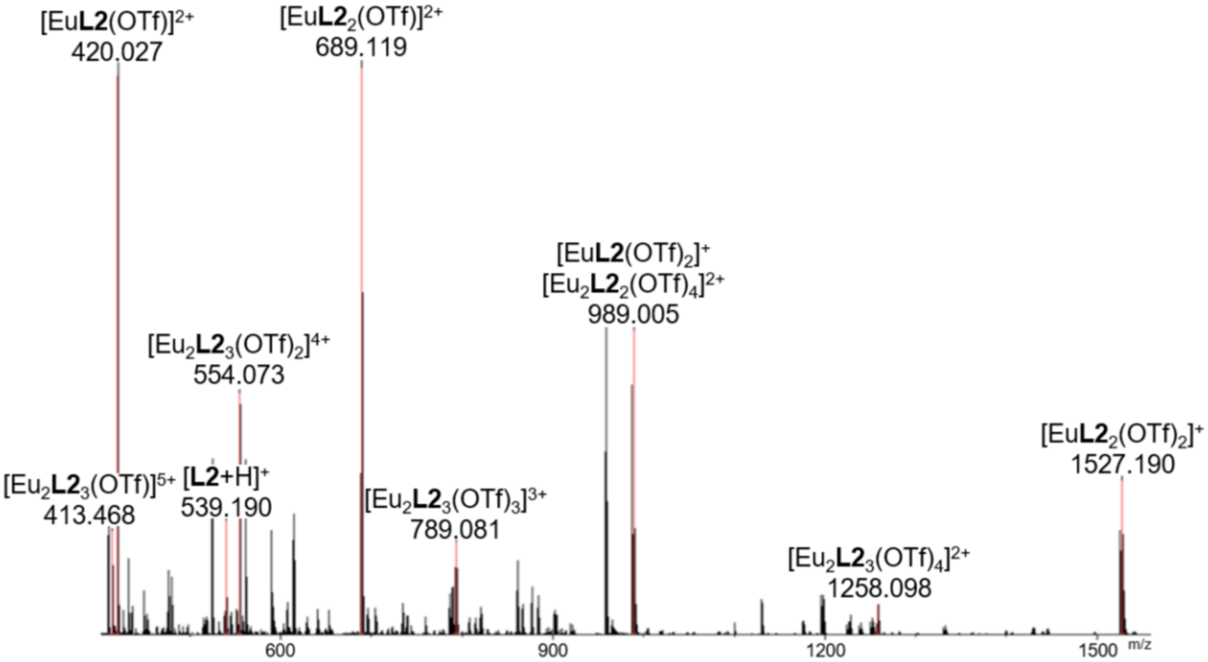

Figure S41. HR-ESI mass spectrum of the complexation products of $Eu(OTf)_3$ and **L2** in 2:3 stoichiometry in CD_3CN.

Table S7. Comparison between the observed and the simulated monoisotopic m/z peaks for complexation products of **L2** and $Eu(OTf)_3$ in a 3:2 **L2**:Eu stoichiometry.

Species	Formula	Simulated m/z	Observed m/z
$[\mathbf{L2} + H]^+$	$[C_{30}H_{27}N_4O_6]^+$	539.193	539.19
$[Eu\mathbf{L2}(OTf)]^{2+}$	$[C_{31}H_{26}EuF_3N_4O_9S]^{2+}$	420.029	420.027
$[Eu\mathbf{L2}(OTf)_2]^+$	$[C_{32}H_{26}EuF_6N_4O_{12}S_2]^+$	989.01	989.005
$[Eu\mathbf{L2}_2(OTf)]^{2+}$	$[C_{61}H_{52}EuF_3N_8O_{15}S]^{2+}$	689.121	689.119
$[Eu_2\mathbf{L2}_2(OTf)_4]^{2+}$	$[C_{64}H_{52}Eu_2F_{12}N_8O_{24}S_4]^{2+}$	989.01	989.005
$[Eu_2\mathbf{L2}_3(OTf)]^{5+}$	$[C_{91}H_{78}Eu_2F_3N_{12}O_{21}S]^{5+}$	413.469	413.468
$[Eu_2\mathbf{L2}_3(OTf)_2]^{4+}$	$[C_{92}H_{78}Eu_2F_6N_{12}O_{24}S_2]^{4+}$	554.075	554.073
$[Eu_2\mathbf{L2}_3(OTf)_3]^{3+}$	$[C_{93}H_{78}Eu_2F_9N_{12}O_{27}S_3]^{3+}$	789.085	789.081
$[Eu_2\mathbf{L2}_3(OTf)_4]^{2+}$	$[C_{94}H_{78}Eu_2F_{12}N_{12}O_{30}S_4]^{2+}$	1258.103	1258.098

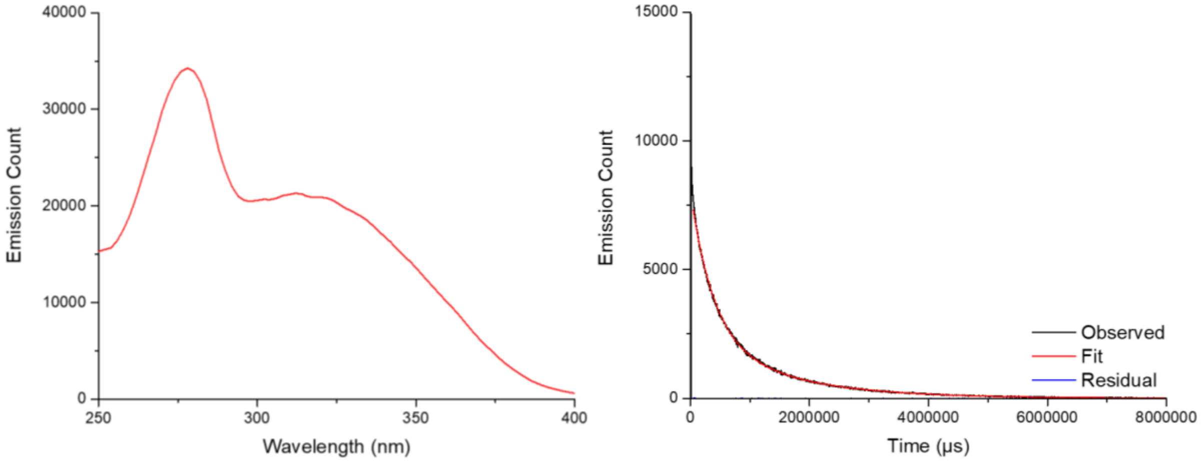

Figure S42. Corrected excitation spectrum of the complexation products of **L2** and $Eu(OTf)_3$ in CD_3CN measured at 617 nm ($^5D_0 \rightarrow {}^7F_2$) (left) and fitted lifetime plot (λ_{exc} = 260 nm and λ_{em} = 617 nm) (right).

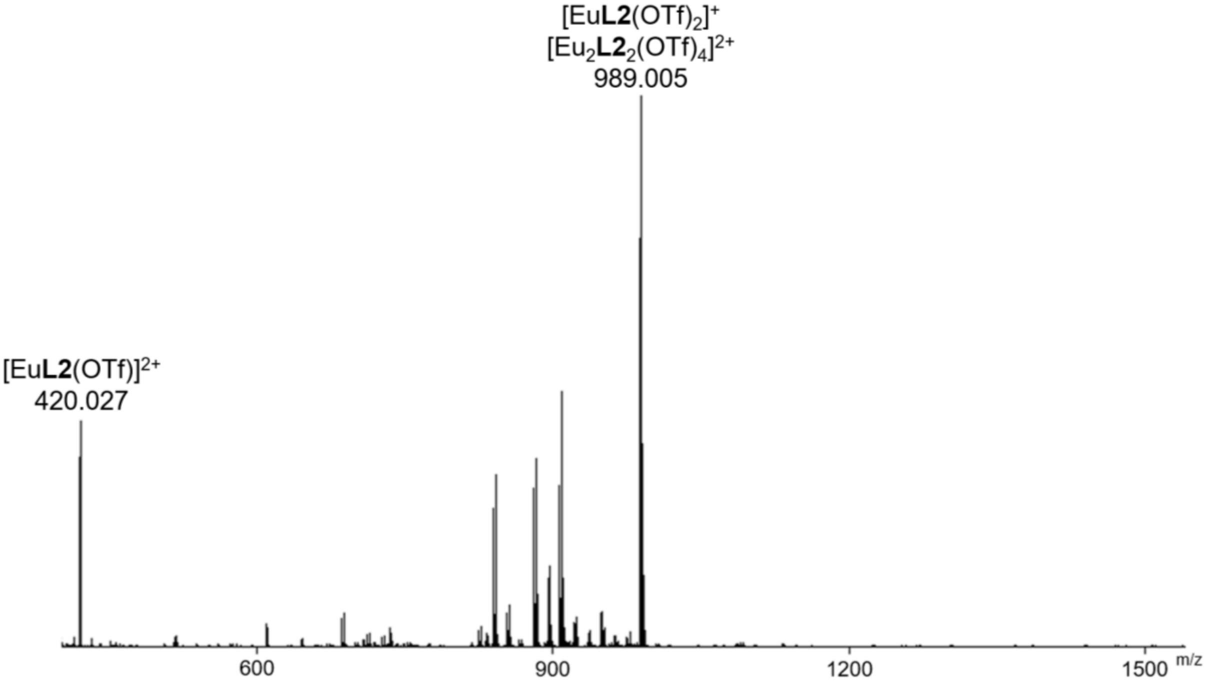

Figure S43. HR-ESI mass spectrum of the complexation products of $Eu(OTf)_3$ and **L2** in 1:1 stoichiometry in CD_3CN.

Table S8. Comparison between the observed and the simulated monoisotopic m/z peaks for complexation products of **L2** and $Eu(OTf)_3$ in a 1:1 **L2**:Eu stoichiometry.

**Species**	**Formula**	**Simulated m/z**	**Observed m/z**
[Eu**L2**(OTf)]$^{2+}$	$[C_{31}H_{26}EuF_3N_4O_9S]^{2+}$	420.029	420.027
[Eu**L2**$(OTf)_2$]$^{+}$	$[C_{32}H_{26}EuF_6N_4O_{12}S_2]^{+}$	989.01	989.005
[Eu_2**L2**$_2$$(OTf)_4$]$^{2+}$	$[C_{64}H_{52}Eu_2F_{12}N_8O_{24}S_4]^{2+}$	989.01	989.005

S2 X-Ray Crystallography

X-Ray data for **L2** were collected at a temperature of 293 K using a synchrotron radiation at the single crystal X-ray diffraction beamline I19 in Diamond Light Source, equipped with a Pilatus 2M detector and an Oxford Cryosystems nitrogen flow gas system. Data were measured using GDA suite of programs. X-Ray data were processed and reduced using CrysAlisPro suite of programmes. The crystal structure was solved and refined against all F^2 values using the SHELXL and Olex 2 suite of programmes.

Crystal data for **L2**:

Identification code	m_iar_db01_009_car_c3_cl1_
Empirical formula	$C_{15}H_{13}N_2O_3$
Formula weight	269.27
Temperature/K	293(2)
Crystal system	monoclinic
Space group	$P2_1/c$
a/ Å	18.8391(8)
b/ Å	6.1722(3)
c/ Å	10.5967(6)
α/ °	90
β/ °	91.035(4)
γ/ °	90
Volume/ Å^3	1231.97(11)
Z	4
ρ_{calc}g/ cm^3	1.452
μ/ mm^{-1}	0.097
F(000)	564.0
Crystal size/mm^3	0.005 x 0.005 x 0.005
Radiation	Synchrotron
2θ range for data collection/°	4.192 to 52.41
Index ranges	$-24 \leq h \leq 24$, $-7 \leq k \leq 7$, $-13 \leq l \leq 13$
Reflections collected	16264
Data/restraints/parameter	2709/60/182
Goodness-of-fit on F2	1.027
Final R indexes [I>=2σ (I)]	R_1 = 0.0519, wR_2 = 0.1284
Final R indexes [all data]	R_1 = 0.0881, wR_2 = 0.1476
Largest diff. peak/hole / e Å^{-3}	0.21/-0.22

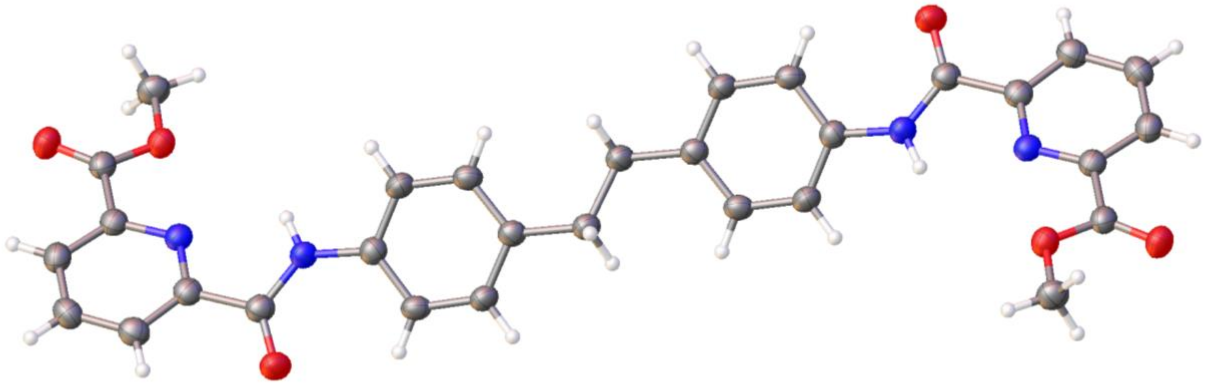

Figure S44. Crystal structure of **L2**, containing two asymmetric unit cells with thermal ellipsoids set at the 50% probability level.

Deposition Number 2291650 (for L**2**), contains the supplementary crystallographic data for this paper. These data are provided free of charge by the joint Cambridge Crystallographic Data Centre and Fachinformationszentrum Karlsruhe Structures service.

S3 Ion Mobility Mass Spectrometry

S3.1. Ion mobility mass spectrometry for CCS determination

Solutions containing **Eu-1**$(OTf)_{18}$ ($[Eu^{3+}]$ = 450 µM) and NaI (250 µM) in acetonitrile were used for all IM-MS experiments. Samples were ionised using a nano-ESI source, and sprayed from borosilicate glass capillaries pulled on Flaming/Brown P-2000 laser puller. A platinum wire was inserted in the capillary to add potential to the sample. Waters SYNAPT XS High Resolution Mass Spectrometer equipped with a travelling wave (TW) ion mobility cell was used for ion mobility experiments in nitrogen drift gas. To determine the corresponding collisional cross-section values ($^{TW}CCS_{N2}$) of the Eu-1 ions, Agilent Tuning mix**[5]** was used for calibration via published calibration procedures.[6] Literature CCS_{He} values,[7] with helium as drift gas, were also used to obtain $^{TW}CCS_{N2\rightarrow He}$ values of the **Eu-1** ions, and insulin**[8]** as well as melittin**[8]** were additionally used as calibrants (**Figure S45**) for that purpose. All samples were measured using the parameters listed in **Table S9**. The CCS of **Eu-1** ions are averaged from three measurements on different days.

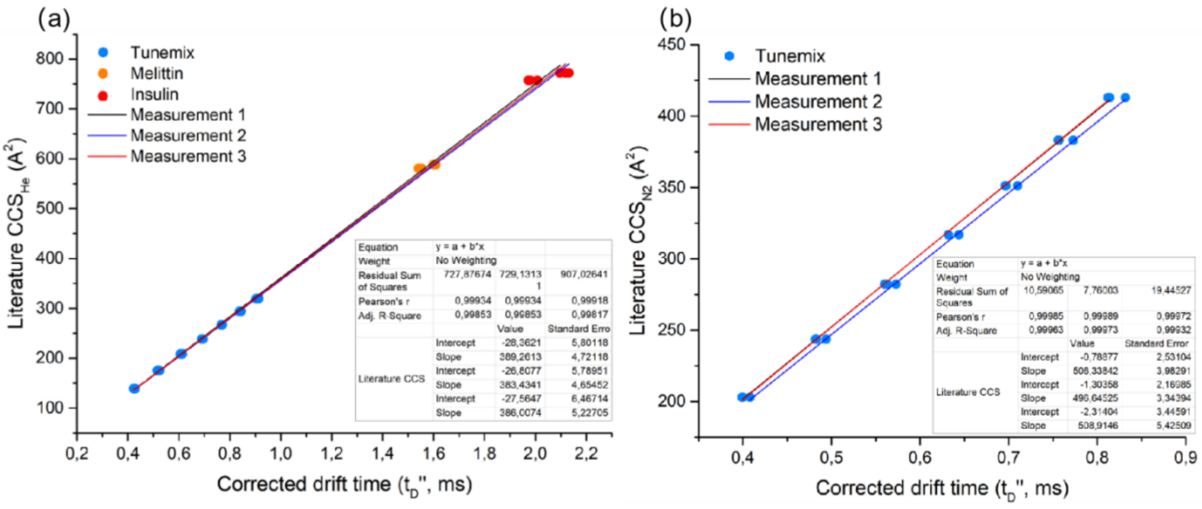

Figure S45. Calibration curve between corrected drift time and calibrant literature values[5b, 7-8] for the calculation of (a) $^{TW}CCSN_2{\rightarrow}He$ and (b) $^{TW}CCSN_2$.

The $^{TW}CCS_{N2\rightarrow He}$ of **Eu-1** ions were calculated alongside the $^{TW}CCS_{N2}$ values for comparison with the hydrodynamic radius obtained from DOSY NMR.[9]

Table S9. SYNAPT XS parameters for calibrants and cage sample measurement.

Voltage (V)		
Trap collision energy	4	
Transfer collision energy	2	
Sampling cone	60	
Capillary	1400-1600	
Gas flow (mL/min)		
Source	0	
Trap	4	
Helium cell	180	
IMS	90	
Travelling wave		
	Wave velocity (m/s)	Wave height (V)
Trap	311	4
IMS	850	40
Transfer	175	4
Travelling wave DC (V)		
Trap DC	Entrance	3
	Bias	45
	Trap DC	0
	Exit	0
IMS DC	Entrance	20
	Helium cell DC	50
	Helium exit	-20
	Bias	3
	Exit	0
Transfer DC	Entrance	5

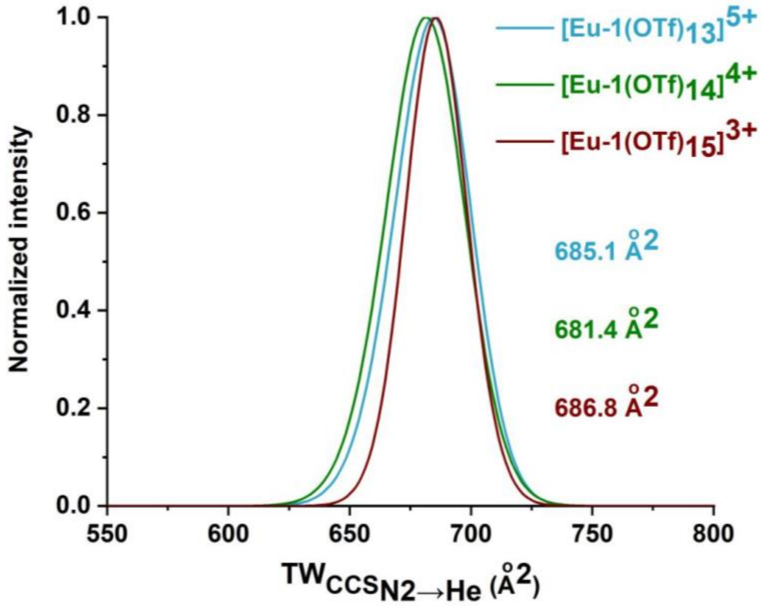

Figure 46. $^{TW}CCS_{N2\rightarrow He}$ distributions for three cations of **Eu-1**. $^{TW}CCS_{N2\rightarrow He}$ values suggest a diameter of 2.96, 2.94 and 2.96 nm for the +3, +4 and +5 ions, respectively. For each ion, one $^{TW}CCS_{N2\rightarrow He}$ distribution from one data set was fitted to a Gaussian distribution.

Table S10. $^{TW}CCS_{N2\rightarrow He}$, $^{TW}CCS_{N2}$ and CCS deduced diameter[10] (D) of **Eu-1** ions. The CCS values are averaged from three measurements recorded on different days.

**Eu-1** cations	$^{TW}CCS_{N2\rightarrow He}$ (Å^2)	$^{TW}CCS_{N2}$ (Å^2)	D($^{TW}CCS_{N2\rightarrow He}$) (nm)	D($^{TW}CCS_{N2}$) (nm)
$[Eu\text{-}1(OTf)_{13}]^{5+}$	685.1 ± 6.7	888.3 ± 9.2	2.96	3.36
$[Eu\text{-}1(OTf)_{14}]^{4+}$	681.4 ± 3.3	870.9 ± 5.5	2.94	3.32
$[Eu\text{-}1(OTf)_{15}]^{3+}$	686.8 ± 4.6	860.8 ± 4.7	2.96	3.32

S3.2. Collision induced dissociation (CID) study

In order to test the stability of **Eu-1**, activated IM-MS studies were performed on the ion [**Eu-1**$(OTf)_{13}]^{5+}$ (m/z = 1234). This ion does not overlap with aggregates or other ions and is therefore suitable for CID studies. [**Eu-1**$(OTf)_{13}]^{5+}$ was accelerated with a series of user-defined voltages (0-18 eV) in order to collide with argon resulting in fragmentation. As shown in **Figure S47**, cage debris was observed even without collisional activation due to spontaneous disassembly. With the increment of collision energy (CE), it is obvious that larger fragments showed decreasing intensity and dissociated to smaller ions (m/z<1000), but the [Eu**L1**$(OTf)_2]^+$ and [Eu_2**L1**$(OTf)_5]^+$ peaks at m/z 1004 and 1602 are always the two most abundant fragments regardless of collision energy.

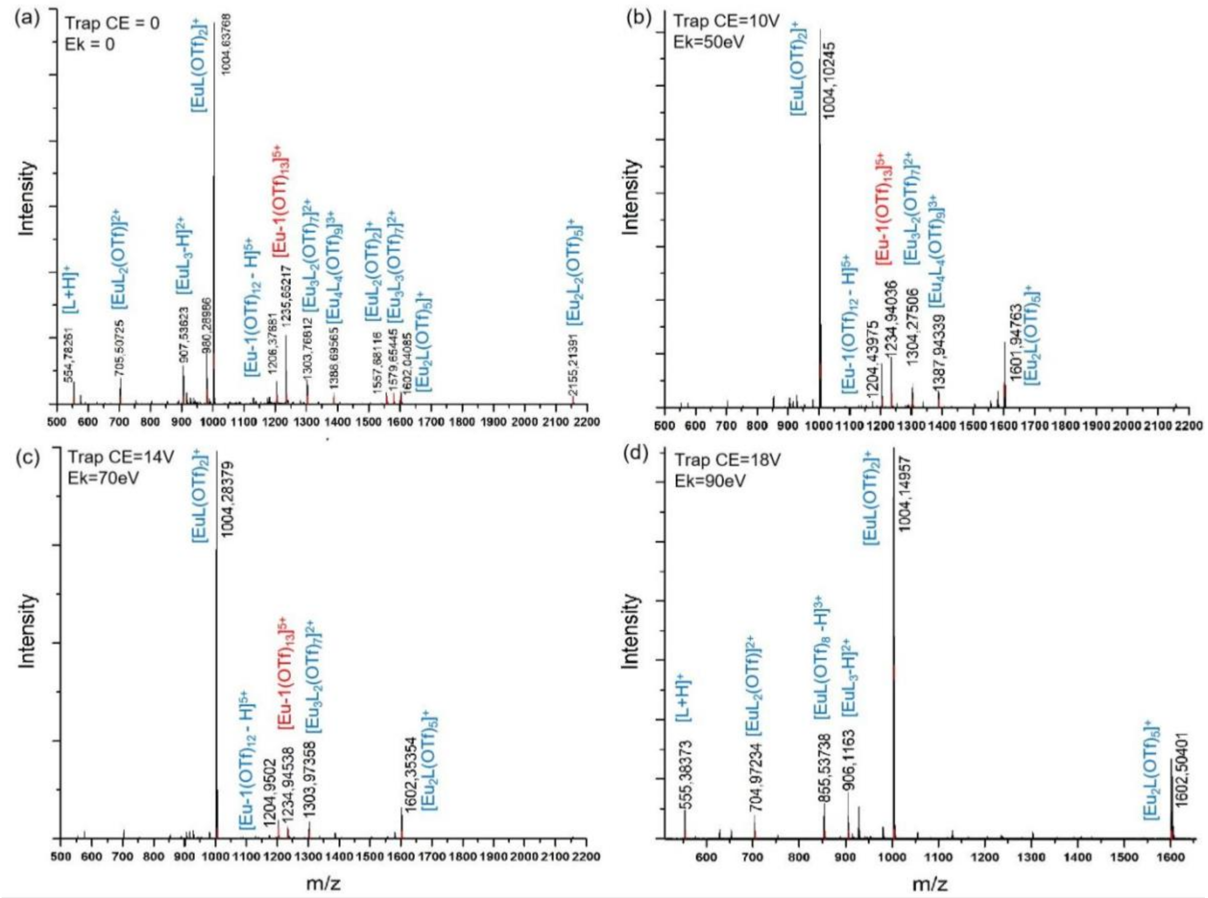

Figure S47. Activated tandem mass spectra of [**Eu-1**$(OTf)_{13}]^{5+}$ with various trap collision voltages: (a) 0 V, (b) 10 V, (c) 14 V, and (d) 18 V. Ek refers to the kinetic energy that the accelerated precursor ion (highlighted in red) experiences, which depends on the charge z.

Three mechanisms are proposed for the cage dissociation. Firstly, the **Eu-1** cage can disassemble through a homolytic process (**Figure S48a**), generating $[Eu_3$**L1**$_3(OTf)_6]^{3+}$ and $[Eu_3$**L1**$_3(OTf)_7]^{2+}$. The trimer $[Eu_3$**L1**$_3(OTf)_6]^{3+}$ fragments then to three [Eu**L1**$(OTf)_2]^+$ ions. Under harsh conditions (70-90 eV), the ion $[Eu_3$**L1**$_3(OTf)_7]^{2+}$ peaks fragment further to [Eu**L1**$(OTf)_2]^+$ and $[Eu_2$**L1**$_2(OTf)_5]^+$, or $[Eu_2$**L1**$(OTf)_5]^+$ and [Eu**L1**$_2(OTf)_2]^+$. The fragments with two ligands subsequently lose one and become the two major ions at m/z 1004 and 1602. Similarly, the cage can be separated to Eu_2**L1**$_2$ and Eu_4**L1**$_4$ species through heterolytic fragmentation (**Figure S48b**). At higher collision energy, $[Eu_4$**L1**$_4(OTf)_9]^{3+}$ also goes through homolysis and heterolysis, and its fragments will be further dissociated as described before. Almost every fragmentation route ends up with a [Eu**L1**$(OTf)_2]^+$ and $[Eu_2$**L1**$(OTf)_5]^+$ fragment, which explains their high abundance in all MSMS spectra. In addition, it is observed that the cage ion may lose a triflic acid (CF_3SO_3H) to form [**Eu-1**$(OTf)_{12}$ - H$]^{5+}$, which can fragment

similarly (**Figure S48c**). This pathway generates smaller ions (m/z < 1000) at higher collisional activation (70-90eV).

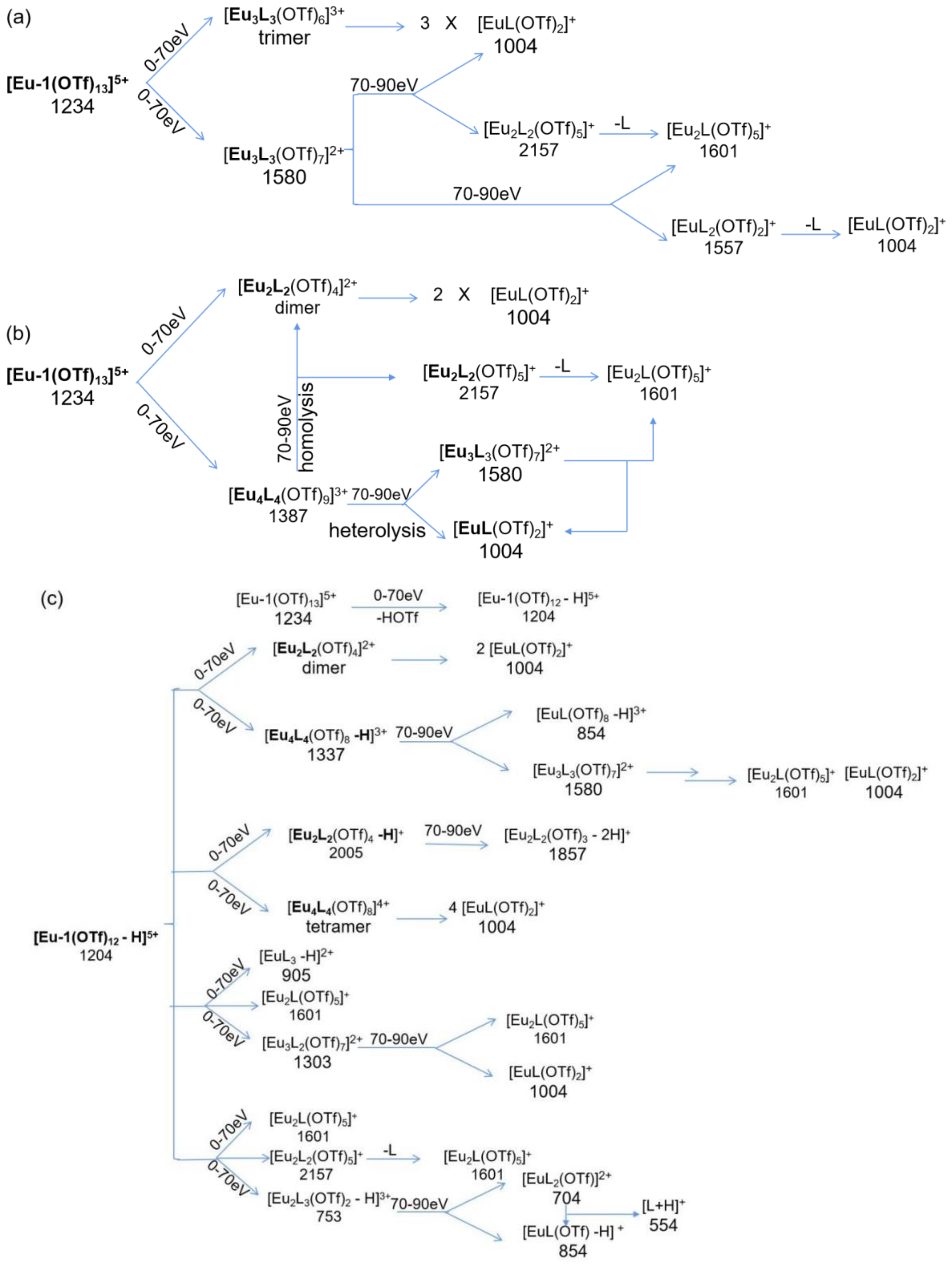

Figure S48. Pathways of **Eu-1** cage ion dissociation: (a) homolytic fragmentation, (b) heterolytic fragmentation, and (c) the initial loss of one triflic acid.

The arrival time distributions (ATDs) of [**Eu-1**$(OTf)_{13}]^{5+}$ and two main fragments were studied under different collisional energies (**Figure S49**). With increasing trap CE, the intensity of the precursor reduced, while its fragments increased. However, the ATDs remain narrow and gaussian-shaped, showing that the cage is rigid and that collisional activation does not significantly change its conformation. This is in contrast to proteins which are prone to collisional unfolding, therefore broadened distributions at higher arrival times are usually observed at higher collision energies.[11]

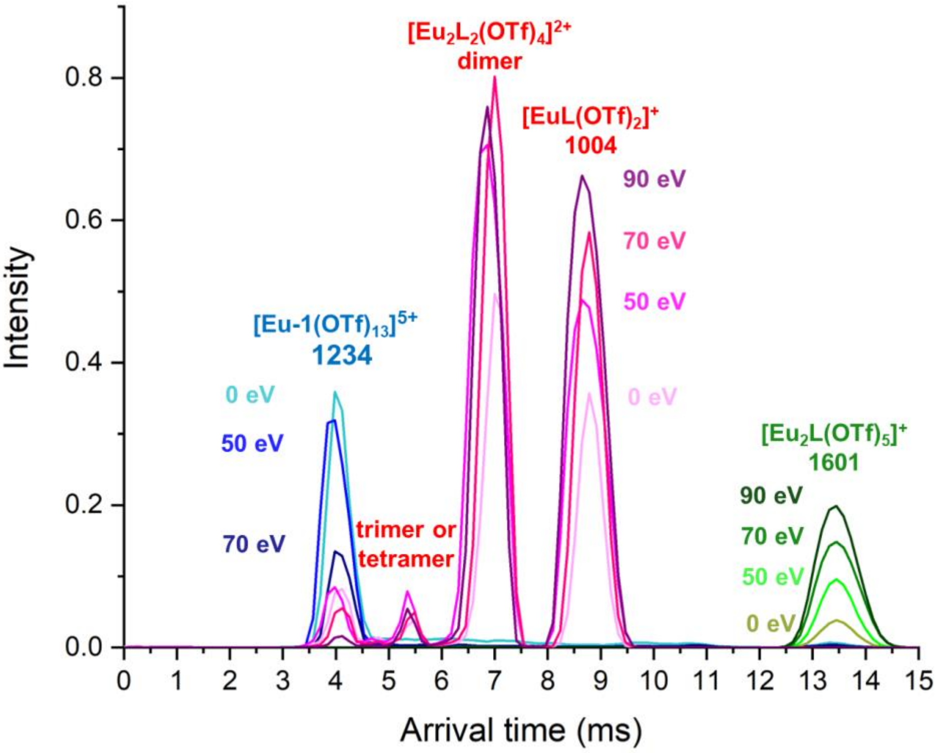

Figure S49. Arrival time distributions of [**Eu-1**$(OTf)_{13}]^{5+}$(blue), [Eu**L1**$(OTf)_2]^+$ and its aggregates (pink) and [Eu_2**L1**$(OTf)_5]^+$ (green) under different collisional energy. The distributions of [Eu**L1**$(OTf)_2]^+$ and [**Eu-1**$(OTf)_{13}]^{5+}$ partially overlap due to the dissociation of the cage ion after passing through the IM cell, and hence its m/z was detected at the same values as the fragment.

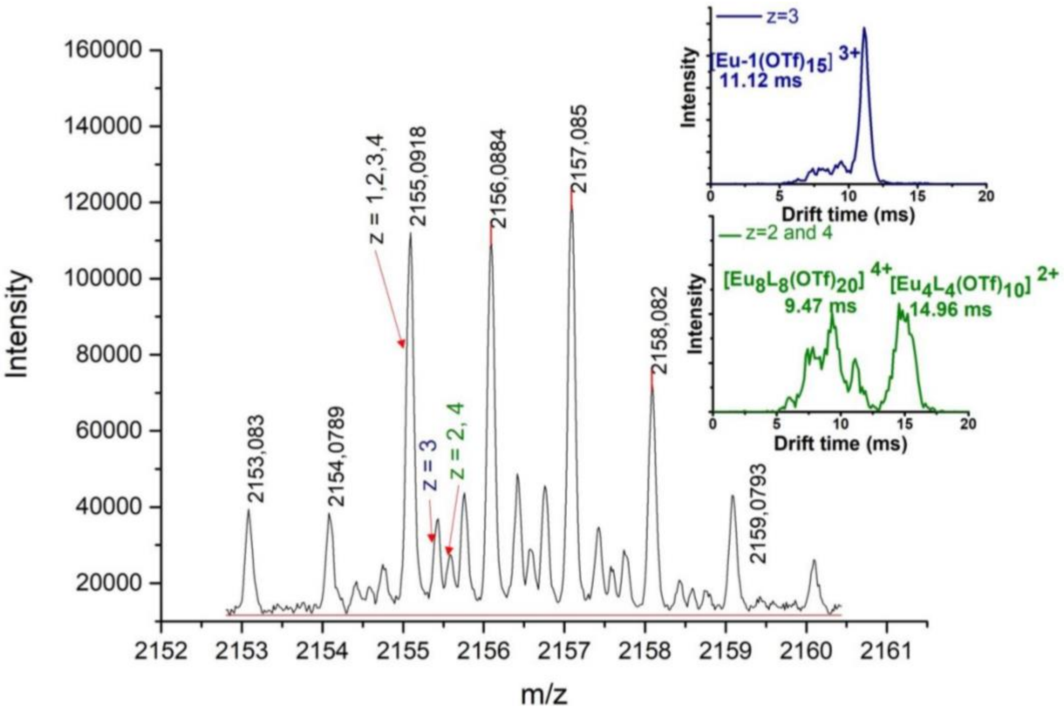

Figure S50. MS spectrum of [**Eu-1**(OTf)$_{15}$]$^{3+}$ overlapping with [Eu$_4$**L1**$_4$(OTf)$_{10}$]$^{2+}$and [Eu$_8$**L1**$_8$(OTf)$_{20}$]$^{4+}$. Insets showing drift time of [**Eu-1**(OTf)$_{15}$]$^{3+}$ (blue) and [Eu$_4$**L1**$_4$(OTf)$_{10}$]$^{2+}$and [Eu$_8$**L1**$_8$(OTf)$_{20}$]$^{4+}$ ions (green).

S4 Proposed structure of Eu-1

S4.1. Modelling of Eu-1

A 3-dimensional model of [**Eu-1**$(OTf)_{14}]^{4+}$ was generated using the Avogadro software.[12] The central amide moiety in **L1** was modelled in both the *cis* and *trans* configurations and its structure is improved using the "Auto Optimization" function with UFF force field and four steps per update.

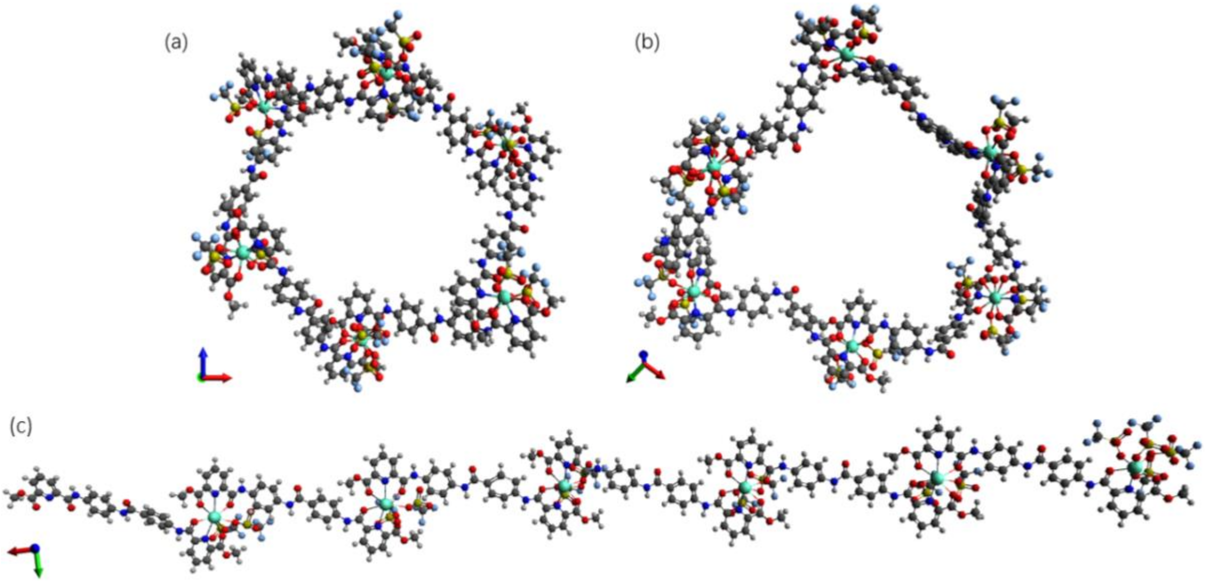

Figure S51. 3D models of [**Eu-1**$(OTf)_{14}]^{4+}$ in (a) circular helicate structure with *trans* amide ligands (b) circular helicate structure with *cis* amide ligands and (c) linear complex with *trans* amide ligands.

Table S11. Ln···Ln distances measure from models of [**Eu-1**$(OTf)_{14}]^{4+}$.

	Average Gd···Gd Distance (nm)		
Model	$Ln_1 \cdots Ln_2$	$Ln_1 \cdots Ln_3$	$Ln_1 \cdots Ln_4$
Circular helicate - *trans*	1.8 ± 0.07	3.1 ± 0.2	3.5 ± 0.4
Circular helicate - *cis*	1.6 ± 0.1	2.8 ± 0.3	3.2 ± 0.2
Linear	1.9 ± 0.05	4.0 ± 0.05	6.0 ± 0.04

Table S12. Diameter measurements for **Eu-1** complexes.

	Diameter (nm)
DOSY	3.04
IM-MS – $^{TW}CCS_{N2\rightarrow He}$	2.95
IM-MS - $^{TW}CCS_{N2}$	3.33
trans amide model	4.01
cis amide model	3.83

S4.2. Potential head to tail isomer formation with unsymmetric ligand L2

The proposed structure of the Eu-1 species is a circular helicate. With an unsymmetric ligand eight different isomers are possible depending on the number and relative orientation of the ligands with respect to each other (H>T or T>H). Without further information the presence and ratio of each isomer within Eu-1 cannot be determined.

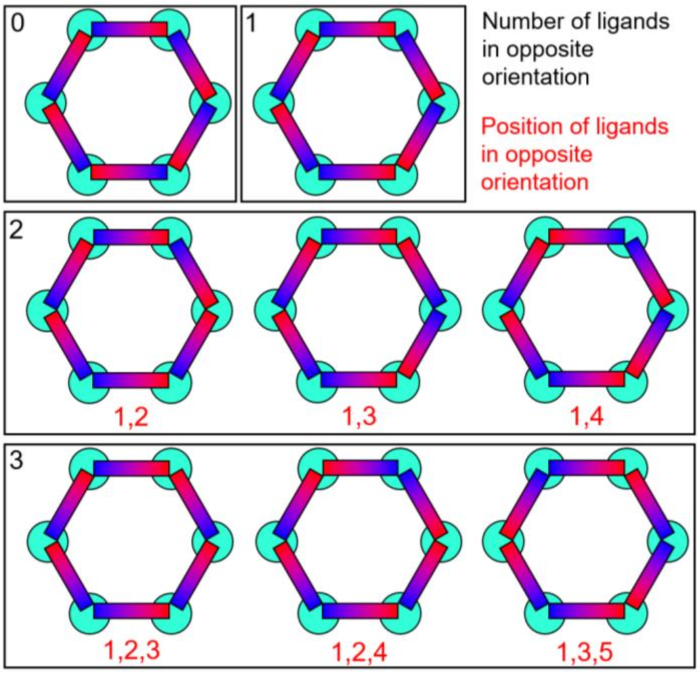

Figure S52. Diagram illustrating the eight possible isomers of hexanuclear circular helicates consisting of unsymmetric ditopic ligands.

S5 EPR Measurements

EPR measurements on **Gd-1** were carried out at Q-band (ca. 34 GHz) at 5 K on a Bruker ELEXSYS 580 FT spectrometer using the EN5107D2 resonator. Low temperature measurements were collected using a Cryogenic cryogen free variable temperature cryostat. All pulses were amplified via a pulsed travelling wave tube (TWT) amplifier. For all measurements, a sample of **Gd-1** was prepared to a final concentration of 200 μM in CD_3CN: d_8-toluene/7:3 in a 1.6 mm O.D quartz tube to a fill height of 1 cm, the sample was then stored at -80°C before it was flash frozen in liquid N_2 and inserted into the cooled resonator.

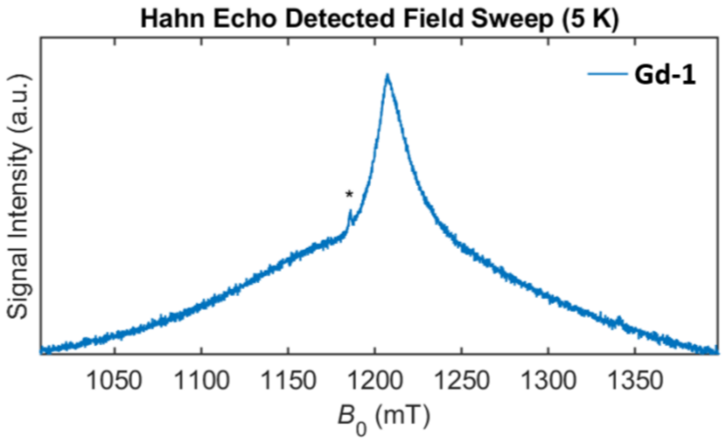

Figure S53. Hahn Echo Detected Field Sweep (EDFS) of **Gd-1** at 5 K using 8/16 ns rectangular pulses at 33.62 GHz (blue). The asterisk denotes a feature corresponding to a small impurity of the resonator background.

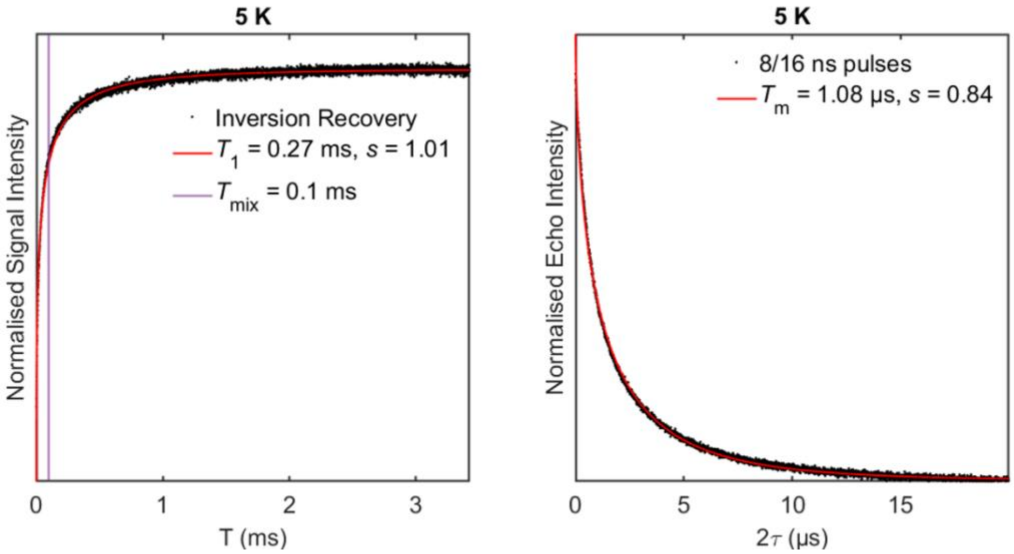

Figure S54. Experimental longitudinal relaxation time (T_1) of **Gd-1** measured at the maximum of the field swept spectrum at 5 K (1207.2 mT, 33.62 GHz) fit to a stretched exponential with stretching factor, *s*, with the value of T_{mix} used in the RIDME experiment shown, ca. 0.4 x T_1 = 0.1 ms (purple line) (left). Experimental phase memory time (T_m) of **Gd-1** measured at the maximum of the field swept spectrum at 5 K (1207.2 mT, 33.62 GHz) fit to a stretched exponential with stretching factor, *s* (right).

A large distribution in zero-field splitting (ZFS) parameters can dampen dipolar oscillations particularly for shorter distances,[13] and given the limited frequency offset between the observer and pump pulses achievable within the bandwidth of the resonator, resulted in Double

Electron-Electron Resonance (DEER) measurements that had low signal-to-noise ratios (SNR) and no obvious dipolar oscillations. To extract inter-spin distance information from the complex, Relaxation Induced Dipolar Modulation Enhancement (RIDME) experiments were carried out on **Gd-1**. Although overtone frequencies (i.e. those which occur at $\Delta m_S \omega_{DD}$, where $\Delta m_S > 1$, and ω_{DD} is the dipolar interaction frequency) in high-spin RIDME measurements can result in the appearance of spurious shorter distances in the distribution, the real distance peaks are likely to dominate the distance distribution, with overtones contributing to a broadening of the distributions, particularly at the shorter edges, as has been seen previously for high-spin RIDME experiments in Gd(III) complexes.[14]

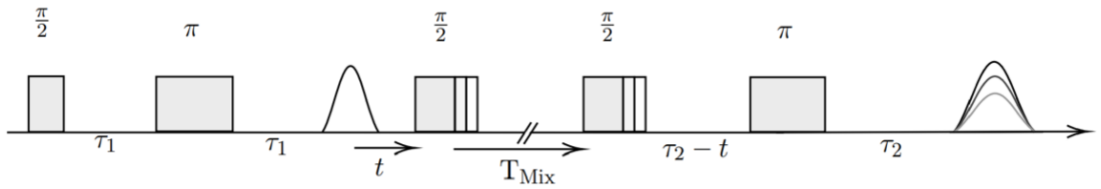

Figure S55. Dead-time free five pulse Relaxation-Induced Dipolar Modulation Enhancement (RIDME) pulse sequence.

RIDME experiments were performed observing at the maximum of the EDFS spectrum of **Gd-1** at 5 K, corresponding to the central transition (1207.2 mT, 33.62 GHz). An 8-step phase cycle was applied to remove unwanted echo coherence pathways, and deuterium Electron-Spin Echo Envelope Modulation (ESEEM) was partially removed from the trace by τ-averaging (8 increments of 16 ns steps). The RIDME trace was analysed using DEERNet's neural network analysis routine,[15] implemented in DeerAnalysis2022,[16] automating the processing of the experimental time trace by applying a background correction, fitting the data and generating a distance distribution for the inter-spin distances with a 95% confidence interval as shown in the main text (Figure 3 c,d). The details of the automated processing are shown in **Table S13**. Analysis including the multi-spin option in DEERAnalysis2022 gave negligible differences in the final fit and distance distribution, while the automated neural network was unable to analyse the trace when the RIDME background option was turned on.

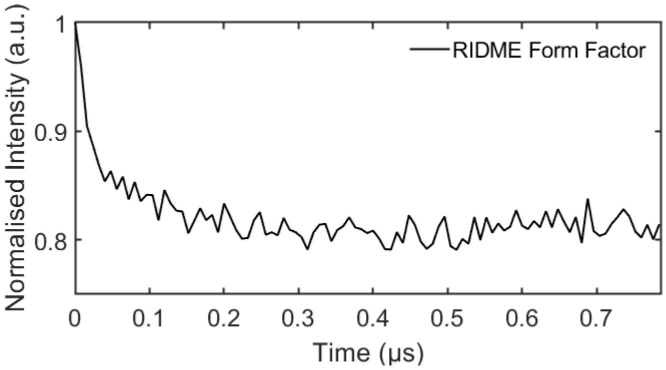

Figure S56. RIDME Form Factor for **Gd-1** after subtraction of the neural network determined background from the experimental time trace.

Table S13. Parameters determined from the automated neural network fitting routine in DEERAnalysis2022.

	Without multi-spin suppression
Mod. depth (%)	18.9
Signal-to-noise ratio w.r.t mod. depth	26.1
Number of networks	32
Background dimension	3

S6 References

[1] G. Canard, S. Koeller, G. Bernardinelli, C. Piguet, *J. Am. Chem. Soc.* **2008**, *130*, 1025-1040.

[2] M. Czerny, A. F. Turner, *Zeitschrift für Physik* **1930**, *61*, 792-797.

[3] aA. Jerschow, N. Müller, *J. Magn. Reson.* **1996**, *123*, 222-225; bA. Jerschow, N. Müller, *Journal of Magnetic Resonance* **1997**, *125*, 372-375.

[4] L. Castañar, G. D. Poggetto, A. A. Colbourne, G. A. Morris, M. Nilsson, *Magn. Reson. Chem.* **2018**, *56*, 546-558.

[5] aA. Marchand, S. Livet, F. Rosu, V. Gabelica, *Analytical Chemistry* **2017**, *89*, 12674-12681; bS. M. Stow, T. J. Causon, X. Zheng, R. T. Kurulugama, T. Mairinger, J. C. May, E. E. Rennie, E. S. Baker, R. D. Smith, J. A. McLean, S. Hann, J. C. Fjeldsted, *Analytical Chemistry* **2017**, *89*, 9048-9055.

[6] B. T. Ruotolo, J. L. P. Benesch, A. M. Sandercock, S.-J. Hyung, C. V. Robinson, *Nat. Protoc.* **2008**, *3*, 1139-1152.

[7] A. Marchand, S. Livet, F. Rosu, V. Gabelica, *Anal. Chem.* **2017**, *89*, 12674-12681.

[8] *Collision Cross Section Database*, *Bush Laboratory, accessed on 2022-04-20*.

[9] P. Bonakdarzadeh, F. Topić, E. Kalenius, S. Bhowmik, S. Sato, M. Groessl, R. Knochenmuss, K. Rissanen, *Inorg. Chem.* **2015**, *54*, 6055-6061.

[10] aA. Kiesilä, L. Kivijärvi, N. K. Beyeh, J. O. Moilanen, M. Groessl, T. Rothe, S. Götz, F. Topić, K. Rissanen, A. Lützen, E. Kalenius, *Angew. Chem. Int. Ed.* **2017**, *56*, 10942-10946; bR. Beveridge, S. Covill, K. J. Pacholarz, J. M. D. Kalapothakis, C. E. MacPhee, P. E. Barran, *Anal. Chem.* **2014**, *86*, 10979-10991.

[11] S. M. Dixit, D. A. Polasky, B. T. Ruotolo, *Curr. Opin. Chem. Biol.* **2018**, *42*, 93-100.

[12] aM. D. Hanwell, D. E. Curtis, D. C. Lonie, T. Vandermeersch, E. Zurek, G. R. Hutchison, *J. Cheminform.* **2012**, *4*, 17; b*Avogadro: an open-source molecular builder and visualization tool.*, Version 1.2.0, avogadro.cc/.

[13] aM. Gordon-Grossman, I. Kaminker, Y. Gofman, Y. Shaic, D. Goldfarb, *Phys. Chem. Chem. Phys.* **2011**, *13*, 10771-10780; bH. EL Mkami, R. I. Hunter, P. A. S. Cruickshank, M. J. Taylor, J. E. Lovett, A. Feintuch, M. Qi, A. Godt, G. M. Smith, *Magn. Reson.* **2020**, *1*, 301-313.

[14] K. Keller, V. Mertens, M. Qi, A. I. Nalepa, A. Godt, A. Savitsky, G. Jeschke, M. Yulikov, *Phys. Chem. Chem. Phys.* **2017**, *19*, 17856-17876.

[15] S. G. Worswick, J. A. Spencer, G. Jeschke, I. Kuprov, *Sci. Adv.* **2018**, *4*, eaat5218.

[16] L. Fábregas Ibáñez, G. Jeschke, S. Stoll, *Magn. Reson.* **2020**, *1*, 209-224.
